# Optimizing Control Strategies for the Cotton Whitefly *Bemisia tabaci*: Insights from Individual-Based Modeling

**DOI:** 10.1021/acs.est.5c13117

**Published:** 2026-01-20

**Authors:** Andre Gergs, Angelika Weinhold, Elena Hettmann, Mariana Durigan, Lokeshkumar Kadu, Jocelyn Kratchmer, Christian Marienhagen

**Affiliations:** † 1569 Bayer AG, Alfred-Nobel Strasse 50, 40789 Monheim, Germany; ‡ Bayer SA − Brazil, Avenida Doutor Roberto Moreira, 5005 Recanto dos Pássaros, Paulínia, SP 13148-914, Brazil; § Bayer CropScience Limited, Bayer House, Central Avenue, Hiranandani Estate, Thane 400607, Maharashtra, India

**Keywords:** population dynamics, toxicokinetic-toxicodynamic model, dynamic energy budget (DEB) theory, agent-based model, whitefly control, ketoenol insecticide, crop protection

## Abstract

The whitefly, *Bemisia tabaci*, significantly threatens agricultural productivity through crop damage and virus transmission. This study developed and parametrized a dynamic energy budget theory-based toxicokinetic-toxicodynamic (TKTD) model to assess the mortality of immature whitefly stages and the impacts of spidoxamat exposure on fecundity and fertility in adults. The TKTD model was integrated into an individual-based model (IBM) to predict population dynamics and efficacy under field conditions, validated with field trial data from different locations in India, Pakistan, and Brazil. The integrated model identified optimal application strategies across varying pest pressure and temperature regimes. The IBM simulation results indicate that a second application can substantially enhance population control, particularly when timed between 7 and 14 days postinitial treatment, depending on ambient temperature and population structure. This timing is influenced by the efficacy half-life and the developmental duration of immature stages, emphasizing the importance of precise application strategies in managing *B. tabaci* populations effectively. The findings underscore the importance of integrating empirical data with modeling for a mechanistic understanding of effects and the development of effective pest management strategies in the face of evolving agricultural challenges.

## Graphical abstract

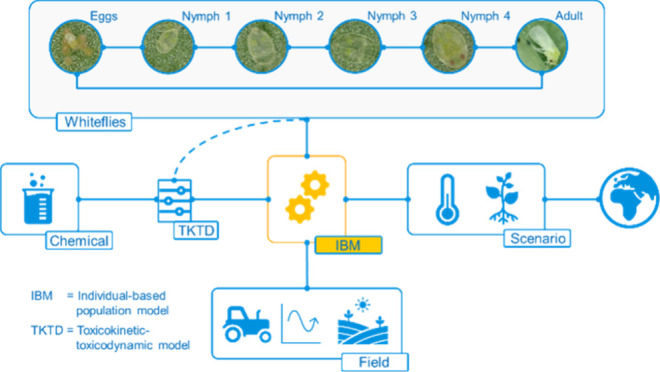

## Introduction

Populations of the whitefly*Bemisia tabaci* Gennadius (Homoptera: Aleyrodidae) can cause detrimental effects on crop health and yield.*B. tabaci* belongs to a species complex that feeds on a wide variety of host plants and is recognized for its capacity to transmit numerous plant virus species, intensifying the difficulties encountered by farmers due to its extensive effects on various cropping systems.^1,2^ Chemical control strategies play a significant role in managing these pests. Selective insecticides, including neonicotinoids and insect growth regulators, can effectively reduce whitefly populations.^3,4^ However, the emergence of insecticide resistance necessitates careful monitoring and rotation of chemical classes to maintain efficacy.^5^ Additionally, precise applications, considering the species' life cycle, enhance overall effectiveness and sustainability in managing whitefly infestations.

*B. tabaci* has a life cycle consisting of four basic stages (see e.g. the review by Walker and colleagues^6^): Adult females lay eggs on leaf undersides, which hatch into nymphs that feed on plant sap and undergo three molts before the pseudopupa stage. Immature life stages are functionally immobile and closely associated with the surface of their host plant, except for early first instar crawlers. Nevertheless, the dispersal potential of the crawler stage is limited, and both host and leaf selection and long-distance dispersal are influenced by adults. The life cycle can be completed within 3 weeks, enabling rapid population growth depending on factors such as temperature, humidity, and host plant availability, which significantly influence population dynamics.^7,8^ Numerous laboratory studies have assessed basic demographic quantities, such as generation time, net reproductive rate, and intrinsic rates of increase, or alike, across various host plants and temperatures, indicating a well-documented potential for rapid population growth, e.g., refs 9–11 . Understanding and predicting these dynamics is vital for effective pest management.^12^

Phenological models have been developed for analyzing life table data and predicting varying population growth potentials under different temperature regimes.^8,9,11,13^ These models allow simulating developmental rates, life stage transitions, oviposition, and/or survival as a function of temperature in a process-based manner. However, phenological models and other empirical approaches examine dependencies separated by life stages, typically overlooking the physiological processes within individual organisms. Dynamic Energy Budget (DEB) theory, by contrast, offers a conceptual and quantitative framework to address an organism’s life cycle as a whole.^14,15^ DEB models allow one to analyze organism performance in dynamic environments, e.g., in relation to temperature and food availability, taking energy and mass balances into account. They have been created for a wide range of species,^16^ including insects, e.g., refs 17–20 . When integrated with a toxicokinetic-toxicodynamic (TKTD) module, DEB models generally enable the simulation of chemical effects on life history traits.^21,22^ TKTD models explicitly take the processes leading to effects such as increased mortality,^23^ reduced fertility and fecundity,^24^ or developmental delay^25^ into account, by mechanistically linking chemical exposure to apparent toxicity.^26^ TKTD models have been employed to study, e.g., effects upon time variable exposure,^27,28^ temperature dependent effects,^29–31^ and life stages sensitivity.^32^

While phenological and DEB models generally capture life table data, including key population components like birth and mortality rates at the individual level, population models incorporate additional factors such as immigration, emigration, and biological interactions.^7,12^ Individual-based population models (IBMs) are particularly esteemed for their capacity to translate individual-level effects into higher-level biological responses.^33^ This class of models simulates population dynamics based on the properties of the individual, accounting for performance under varying environmental conditions and biological interactions. Since their introduction over a decade ago, IBMs grounded in DEB theory, referred to as DEB-IBMs,^34^ have been developed and applied, e.g., in studies of biotic interactions such as competition and predation,^35–37^ as well as chemical effects.^32,38,39^

In this study, we developed a DEB-IBM for *B. tabaci* to investigate optimal control strategies for the species in cotton, *Gossypium hirsutum* L., using the insecticide Spidoxamat as a case study. Spidoxamat, a spirocyclic tetramic acid derivative (ketoenol), is the active ingredient in Plenexos, which is the proposed brand name of a new insecticide family. Ketoenols target acetyl-CoA carboxylase, a crucial enzyme in lipid biosynthesis,^40,41^ and have been shown to disrupt organism development, as well as female fecundity and fertility.^40,42^ After uptake by plants, depending on their physicochemical properties, some ketoenols like spirotetramat are translocated via xylem and phloem in a two-way systemic manner, i.e., transported in two directions within the plant,^43^ making them particularly effective against sucking pests like whiteflies and tetranychid mites.^44,45^

The aim of this study was to develop and parametrize a TKTD model to assess the mortality of immature whitefly stages, as well as the effects of spidoxamat exposure on adult fecundity and fertility. This TKTD model was then integrated into a newly developed DEB-IBM for *B. tabaci* to predict population dynamics and spidoxamat efficacy under field conditions. The parametrized DEB-IBM was validated using field trial data from India, Pakistan, and Brazil, and was ultimately applied to identify optimal spidoxamat application strategies for varying pest pressure scenarios and temperature regimes.

## Material and Methods

### Insect Rearing for Greenhouse Trials

Greenhouse trials were carried out using the *B. tabaci* strain SUD-S, originally collected in Sudan and frequently used as an insecticide susceptible reference strain, e.g., ref 46, facilitating consistent results in efficacy assessments. The whitefly populations were maintained on cotton (*G. hirsutum*) under controlled conditions at 25 ± 1 °C, 60% relative humidity, and a photoperiod of 16 h light and 8 h dark.

### Greenhouse Trials

A set of three greenhouse trials was carried out at 23 ± 2 °C and ambient photoperiod (∼L10:D14 h at the time of trial conductance) and replicated three times to assess mortality, fertility, and fecundity effects for different application rates of spidoxamat and aged spray residue. Three-week-old cotton plants with two true leaves were used for the trials. One true leaf was removed from the plant, and the remaining one was infested with whiteflies, as detailed below. For treatments, formulated Spidoxamat WG 4.8. was used to the targeted application rate in terms of grams per hectare in tap water, with the spray solution containing 0.1% rapeseed oil methyl ester (RME EW 500).

#### Trial 1

The remaining cotton leaf was infested with five female adults (6–10 days old) using clip cages on leaf undersides. After 24 h, the adults were removed, and the number of eggs laid was counted. Six to 7 days postinfestation, the cotton leaves received a translaminar application of spidoxamat, i.e., application of the formulation on the upper side of the leaf while whiteflies are located on leaf undersides. The formulation was diluted to concentrations of 24, 6, 3, and 1.5 g of spidoxamat per hectare in tap water and applied as described above. Mortality was assessed on clipped leaf areas 5, 7, and 17 days after treatment.

#### Trial 2

Female fecundity and fertility of eggs laid were quantified following spidoxamat application of 3, 6, 12, and 24 g a.i./ha. The methodology for assessing fecundity and fertility was adapted from ref 47. Briefly, after application, five female adults were caged on treated plants (leaf undersides) on the day of application and at 1-, 2-, and 3-days post-treatment. After 24 h, the adults were removed, and fecundity was assessed by counting the number of eggs laid. Fertility was determined by monitoring hatched nymphs during a period of 8 to 12 days.

#### Trial 3

Fecundity and fertility were quantified to assess the effects of aged residues. Therefore, cotton plants were initially treated with Spidoxamat at a rate of 24 g a.i./ha. Subtrials were done using treated plants at intervals of 1, 7, 9, 14, and 21 days post-treatment. Again, five female adults were caged on leaf undersides on days 0, 1, 2, and 3 of each subtrial, and removed after 24 h for subsequent assessments as described above.

### Cuticle Penetration Test

To explore the impact of the different water volumes used in the field trials, cuticle penetration was quantified for the Spidoxamat WG 4.8 formulation with RME as described above, testing 6 levels of water volumes: 10, 20, 30, 50, 100, and 300 L/ha. The cuticle penetration test was performed with apple cuticles following the methodology described in ref 48. Briefly, a droplet of the spray solution was applied on the outer side of the cuticle, and penetration was subsequently quantified seven times during a period of 24 h. The droplet size was adapted to the concentration of the spidoxamat to ensure a comparable amount of the active substance on the cuticle, independent of the applied water volume. The pH in the spidoxamat spray solution was measured and averaged out at 7.65 (ranged 7.54–7.76). The test was done at a constant relative humidity of 60% and at 25 °C with a rise after 12 h to 35 °C to observe if any significant temperature difference may occur. Spidoxamat concentrations in the test vessels were quantified via HPLC with single quadrupole MS in six to ten replicates per applied water volume.

### Field Trials

The biological efficacy of spidoxamat against *B. tabaci* in cotton was evaluated by nine different field trials from different locations in Brazil, India, and Pakistan each comprising an untreated control and three different treatments, each replicated three to four times. The field studies were conducted at different application rates, ranging from 12–48 g a.i./ha and different application intervals, following Good Agricultural Practices to ensure reliable and safe outcomes. For an overview of the field trials, see Table S2.1 in the Supporting Information (SI). Crops were treated at varying pest infestation levels using standard local application techniques. A backpack sprayer equipped with Turbo TeeJet or hollow cone nozzles was employed at a pressure of 2–3 bar to apply the different rates of spidoxamat treatment. A total water volume of 150–500 L per hectare was used for each treatment to ensure adequate coverage. Temperature data was obtained from nearby weather stations.

### Model Description

The IBM simulates the dynamics of whitefly populations by representing each individual and its interactions within the environment. The model operates at three hierarchical levels: ecosystem, population, and individual. The ecosystem level encompasses environmental variables, such as host plant leaf area, ambient temperature, and chemical exposure concentration, with the spatial scale currently limited to a single plant, assuming homogeneous infestation in a cotton field and ad libitum food availability. At the population level, the model monitors population abundances through a single state variable, the population size, which reflects the number of whiteflies. Changes in population size result from individual births and deaths, and adult immigration and emigration. Each whitefly is represented by a DEB model integrated with a TKTD module, as detailed below. IBM operates in discrete time steps, updating state variables daily. Daily processes include adjusting rate constants for temperature, calculating toxicokinetics and toxicodynamics based on exposure concentration, and assimilating energy for growth, maintenance, and reproduction. Daily mortality probabilities are calculated, and new viable eggs are added to the population count. Emergence of individual traits and population dynamics arises from the metabolic organization. Stochasticity is integrated through probabilistic mortality, migration rates, and initial population composition. Observations include total whitefly counts and developmental stage distributions. Model simulations are initialized based on specific field conditions, including temperature trajectories and population composition. Each individual is randomly assigned a gender and body size, with intermediate states calculated accordingly. Immigration and emigration rates can be specified, and the daily mean temperature serves as the primary input, with exposure concentrations calculated based on application rates and timings. While plant size is not directly an input, it can be used for processing model outputs related to population densities.

Individual whiteflies are represented by a DEB model. The standard DEB model categorizes life into three stages: embryo, juvenile, and adult. The embryo relies on stored maternal energy for growth and maintenance, while the juvenile stage begins feeding to support growth, maturation, and maintenance. In adulthood, energy is primarily allocated for reproduction rather than maturation. Each individual is described using four state variables: reserve, which acts as an energy buffer; structure, energy invested in maturation of juveniles; and energy invested in reproduction in adults. A key aspect of DEB theory is the *k*-rule,^15^ which dictates that a fixed fraction (*k*) of the mobilized energy is allocated for somatic maintenance and growth, while the remaining fraction (1 – *k*) is directed toward maturity maintenance and reproduction. Energy flow within the organism is governed by a scaled functional response with assimilation transforming food into reserves. The mobilization of energy from reserves supports various metabolic processes, including growth, maintenance, and reproduction. In most insects, nymphal growth is rapid and typically exponential, which is captured by the assumption of metabolic acceleration from birth until puberty by the ‘abp’ variant of the standard DEB model.^16^ In this model, birth coincides with hatching and puberty aligns with emergence, whereafter growth ceases during the adult stage. The individual survival probability of an individual is calculated based on a background hazard in nymphs and a hazard due to aging in adults.

For the TKTD model, we utilize a reduced variant in which toxicokinetics and damage dynamics are integrated into a single compartment. The damage may represent either an internal concentration or a form of damage, with the dominant rate constant reflecting either process. Damage is scaled with exposure concentration, having units of the chemical application rate. Given that spray applications in broad-acre crops are usually conducted from above, and considering that the immobile stages of whiteflies are primarily found on the undersides of leaves, we assume that their direct exposure to the spray is negligible. Instead, we focus on translaminar exposure, where chemical uptake and damage occur solely through feeding. Nonfeeding stages, such as eggs and pseudopupae, as well as the third nymphal stage are assumed unexposed. All modeled developmental stages, including adults, can repair the damage. Both sublethal and lethal effects are incorporated, with damage influencing the hazard rate and primary DEB parameters via linear-with-threshold relationships. Chemical stress on metabolic processes is imposed via different physiological modes of action (pMoAs), for an overview see ref 22. Fecundity effects in adults are simulated as ‘increased reproduction costs’, while fertility effects are represented by the pMoA of ‘hazard to embryos’, since we did not observe any effects on body size. Note that, in the IBM, we simulate only the number of viable offspring based on the ‘hazard to embryos’. To model translaminar exposure of whiteflies, we employ a one-compartment model analogous to the scaled damage model mentioned above, to simulate cuticle penetration as a function of water volume used for applications (see SI1 for details). This scaled internal concentration in the plant is assumed to decrease over time due to metabolization and dilution from plant growth, characterized by an exposure decay rate constant.

All model rate constants are corrected for ambient temperature using an Arrhenius function with lower and upper boundaries. The temperature correction is applied to both whiteflies and host plants using distinct parameter sets. Note that for host plant dynamics, the Arrhenius function with upper boundaries was utilized only. A more detailed model description, equations, and parameters are provided in SI1.

### Model Implementation, Parametrization, and Application

The IBM is implemented in Delphi 12 (Embarcadero Technologies Inc. 2024) based on discretized forms of the differential equations provided in SI1. The DEB model for *B. tabaci* was previously published in the add-my-pet collection,^16^ and the code, parameter estimates, as well as underlying data are available online.^49^ Note that the DEB model parametrization is based on literature data for *B. tabaci* Biotype B and various but comparable food sources; for a comparison of life history traits and host plants, see ref 8. The DEB model code in MATLAB (MathWorks Inc. 2019) was extended by the TKTD model equations (see Table S1.1) and parametrized using DEBtool^50^ based on the results from the greenhouse trials described above.

The IBM was validated using population dynamics data from untreated cotton fields, as reported by Naranjo and Ellsworth,^7^ and the nine field trials with Spidoxamt applications described earlier. Mean daily temperatures and calculated exposure scenarios for each of the three treatment groups in the nine field trials served as model inputs (Figure S2.5). Note that the initial application rate is corrected for the water volume used in the respective field trial. To replicate the findings of Naranjo and Ellsworth,^7^ the timing of adult immigration was aligned with their observations (see captions Figures S2.4–S2.6). For the nine field trials involving spidoxamat treatments, initial settings were adjusted to match the dynamics of nymph counts in the untreated controls (Table S2.2). The observed efficacy was then compared to subsequent model predictions, with no additional calibrations performed.

Finally, we performed scenario analyses to systematically explore the efficacy of repeated spidoxamat applications in relation to ambient temperature, application interval, and population factors such as initial stage distribution and adult immigration rates.

The Delphi code for the IBM implementation, executable code in R,^51^ survival and reproduction data, as well as the R code for producing TKTD figures, are provided in SI3.

## Results

TKTD model parameters were estimated simultaneously using data from the three greenhouse trials, with the resulting parameter values listed in Table S1.2. We assumed that lethal effects and sublethal stress on DEB parameters share the same dominant rate and threshold but differ in effect strengths. Additionally, we posited that the pMoA of ‘increased reproduction costs’ and ‘hazard to embryo’ represent fecundity and fertility effects, respectively, and share identical parameter values. The parametrized model, based on these assumptions, closely matched observed survival rates of nymphs as well as fecundity and fertility effects in adults (Figures 1 and S2.1). The quantification of fecundity and fertility effects from the aged residue trial allowed us to estimate a decay rate of *k* = 0.042 d^–1^ (at reference temperature, Table S1.2), corresponding to an effect half-life of approximately 5.5 days at an ambient temperature in the greenhouse at 23 °C. Note that we also assumed an exposure delay of 3.7 days to accurately capture the survival data (see implementation of the survival model in SI3), accounting for the time between spidoxamat application and the onset of exposure through feeding, which includes egg hatching and crawler settlement. Likewise, we estimated delays of 0.6 days for fecundity effects and 0.3 day for fertility effects in Trial 3 (see the respective implementations in SI3), likely reflecting the settlement of adults in this setup.

**Figure 1:**
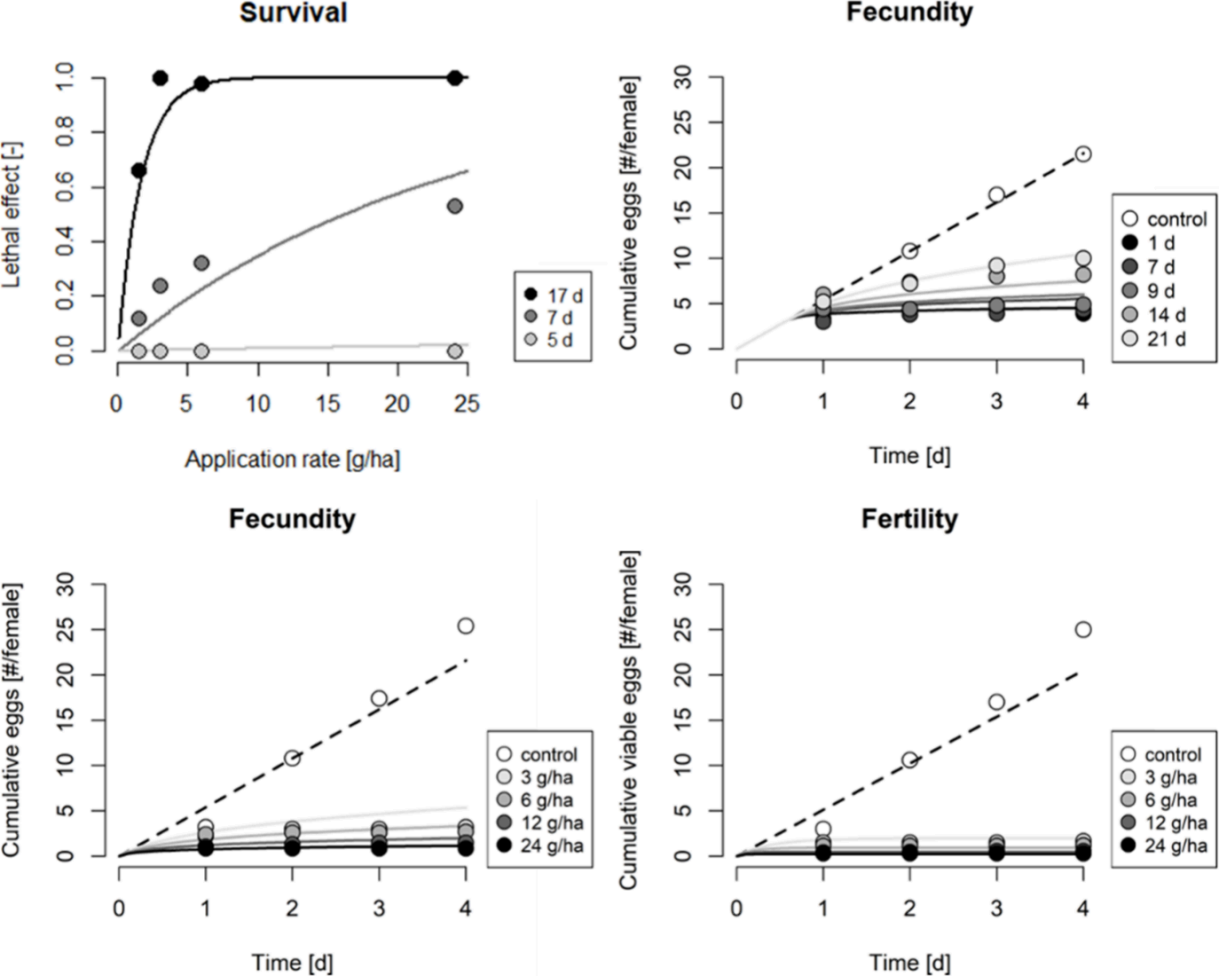
Parametrization of the TKTD model for lethal effects, fecundity, and fertility. Lines and dots represent the model and empirical data, respectively. Effects on survival in whitefly nymphs as a function of spidoxamat application rates were assessed 5, 7, and 17 days after the treatment of eggs (Trial 1, top left). Fecundity and fertility in adult whiteflies as a function of time were quantified in two separate experiments: for different applications rates (Trial 2, bottom), and on plants and aged for 1, 7, 9, 14, and 21 days after an initial spidoxamat exposure at 24 g/h (Trial 3, top right). For the latter experiment, the cumulative number of eggs per female in each experimental subsets were normalized by their respective control. For the fraction of viable eggs quantified on aged plants, see Figure S2.1.

After application, adjuvanted spidoxamat quickly penetrated the tested cuticles, reaching saturation levels within 12 h (Figure S2.2). The subsequent increase in temperature during the experiment hardly enhanced penetration. Additionally, spidoxamat penetration decreased with lower water volumes used for application. In our model, we assumed a linear relationship between maximum penetration and water volume, which describes the data well. As we utilize a scaled model for exposure and effects (see model description), we correct for water volume relative to a maximum of 300 L at saturation when generating the exposure scenario for the IBM, which operates in daily steps. This approach disregards the actual penetration with a maximum of 31%, which is a test-specific value disregarded in the scaled model. Temperature is another factor influencing exposure in our model. We assumed that the decay rate and growth rate of the plant scale with temperature in the same manner; higher growth rates correspond to shorter effective half-lives. Growth rate parameters and temperature correction functions for cotton (Table S1.2) were inferred using data published previously,^52^ indicating a maximum growth rate in terms of leaf area at 28.5 °C (Figure S2.3). Consequently, the effect half-life may decrease to approximately 1.5 days, depending on the temperature. The exposure calculations across the temperature scenarios used in the field trial simulation are presented in Figure S2.7.

For the initial validation step, we compared IBM predictions with population data from untreated cotton fields reported by Naranjo and Ellsworth.^7^ The authors provided detailed demographic information about egg, nymph, and adult densities during cotton growth seasons in Arizona from 1997 to 1999. Weather data for the study are available online, and the temperature trajectories were used as input for our model. The timing of adult immigration in the simulations was aligned with the original observations.^7^ To compare our model output with the reported whitefly densities, we simulated cotton plant growth in terms of leaf area and height, i.e., the number of mainstem nodes (see equations and parameters in SI2). For each time step, we divided the total number of simulated whiteflies per stage by the calculated cotton leaf area. To account for observed emigration, we assumed a 0.95 fraction of emigrating adults once the simulated host plant height exceeded 22 mainstem nodes, considering seedling size initially (see SI1 for details), which described the original observation of adult emigration by Naranjo and Ellsworth^7^ during the three consecutive years well (Figures S2.4–S2.6). In the original study, egg and nymph counts were based on sampled leaf discs (3.88 cm^2^), which we incorporated into our calculations. For adults, counts per leaf were provided, but as the actual leaf area was unknown and likely varied over time, we assumed a default area 20 times larger than the disc for adult densities in our simulations. This approach yielded adequate predictions for population densities observed over the three years (Figures S2.4–S2.6). For 1997, the model closely matched egg-laying dynamics, although deviations in nymph counts became apparent later in the simulated period (Figure S2.4). In 1998, the authors noted higher-than-usual immature mortality in early August, which we accounted for by doubling the background hazard rate during that period, resulting in immature stage counts that did not reach 1997 levels (Figure S2.5). Adult immigration into the observation plots was reported to occur later in 1999 compared to previous years, causing modeled and observed populations to also fall short of 1997 densities (Figure S2.6).

For the ultimate validation step, we compared the model performance against data from nine different field trials involving spidoxamat conducted in Brazil, India, and Pakistan. The trials varied in spidoxamat application rates, the number of applications, application intervals (Table S2.1), ambient temperatures (Figure S2.7), and pest pressure. For each trial simulation, initial model settings were adjusted to match nymph counts in the untreated control plots (Table S2.2). The efficacy of the various treatments was then predicted without further model adjustments and compared with the mean efficacy observed in the trials (Figure 2). Results from all trials are detailed in the SI (Figures S2.8–S2.16). Steadily increasing nymph counts during the trial period, particularly noted in the Pakistan and India trials (Figures S2.12–S2.16), were likely due to ongoing adult migration, as indicated by the settings required to capture these dynamics (Table S2.1). Conversely, fluctuating or decreasing nymph counts in the control plots were best represented by periodically ceasing adult immigration.

**Figure 2:**
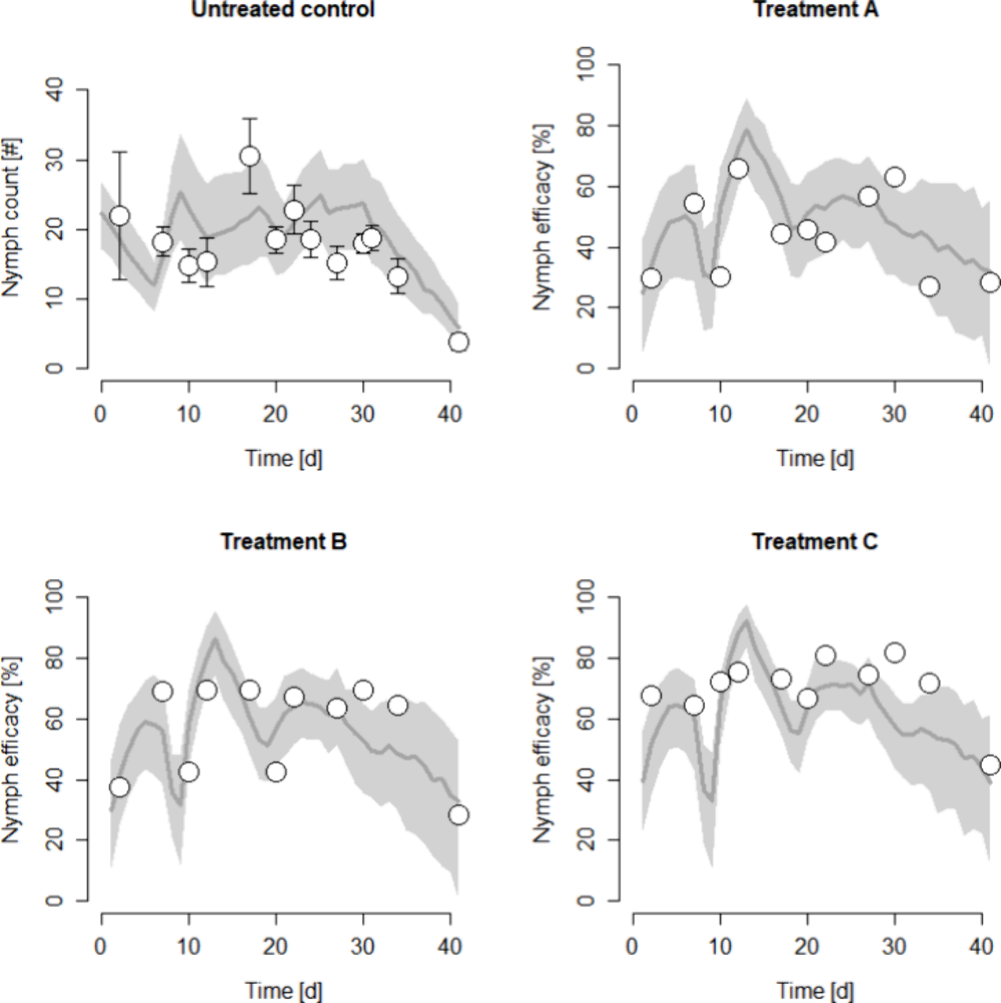
Model validation with the field data. Nymph count and efficacy quantified in Field Trial 2 (for details, see Table S2.1). Dots are measured data (means and standard deviation for counts, and efficacy based on mean counts) while the lines and gray shades represent the median and 95% prediction intervals of 100 Monte Carlo simulations. See SI2 for the complete set of model validations. Treatments were as follow: (A) 3 × 12 g a.i.//ha, (B) 16.8 g a.i.//ha and (C) 24 g a.i.//ha with spidoxamat applied three times, initially and subsequent at intervals of 10 days. The initial model settings are set to meet the nymph count data in the untreated control. Efficacy for the different treatments is subsequently predicted without any further model adjustments.

The observed maximum efficacy on nymphs in percent, defined as one minus the ratio of the number of nymphs in the treatment group to the number of nymphs in the control group times 100, across treatment groups in the trials ranged from 55 to 95%. Single applications of spidoxamat typically peaked in efficacy about 1 week post-treatment, followed by a decline over the next two to 3 weeks (Figures S2.12–S2.16). A second application generally increased the maximum efficacy observed in treatment groups and extended its duration compared to single applications. However, varying intervals between the first and second applications resulted in a drop in nymph efficacy shortly before the second application, particularly with intervals of 10 and 14 days, while these longer intervals maintained efficacy for extended periods later in the trials. A third application also prolonged efficacy, but the data did not allow for a comparison with the two applications due to shorter study durations for the latter treatments.

The model described these efficacy patterns overall well, particularly peak efficacy per treatment group, rates of efficacy decline following single applications, and efficacy drops between applications (Figures S2.8–S2.12). Some deviations between observed data and model predictions were, however, noted. For instance, the onset of effects after the first treatment was slower than that predicted in Trials 3 and 8 (Figures S2.10 and S2.13), while other trials were predicted accurately. Additionally, nymph efficacy in some 7-day interval treatment groups was maintained longer than the model predicted, which anticipated a more rapid decline (Figures S2.13, S2.15, and S2.16). The predicted efficacy was also somewhat higher than that observed in the 7-day interval treatment groups of Trials 5 and 6 (Figures S2.12 and S2.13). Overall, 88% of the observed efficacy data fell within a 20% deviation of the values predicted by IBM, demonstrating good model performance (Figure S2.17).

Since no additional calibration was performed to rather accurately predict efficacies, the differences observed in field trial outcomes can largely be attributed to varying temperature regimes, initial states of the whitefly population, and the migration of mature whiteflies, which served as inputs to IBM. We systematically examined the influence of these factors through scenario analysis within the IBM framework. Figure 3 illustrates the effects of one and two applications of Spidoxamat with varying application timings. In these simulations, we assumed an initially synchronized population of second instar nymphs and two egg-laying adults and an ambient temperature of 30 °C, resulting in an estimated effect half-life of approximately 1.5 days (see above). A single application of 12 g a.i.//ha reduced the population size by a factor of about four, primarily due to the lethal effects on early-stage nymphs and the reduced number of viable eggs laid by adults (Figure 3B). A second simulated application (on day 6) only marginally enhanced the efficacy of the first, as the population remained dominated by eggs, which are not sensitive to spidoxamat exposure (Figure 3C). In contrast, a later application on day 12 effectively targeted nymphs after they hatch from the eggs, resulting in a pronounced reduction in the population (Figure 3D).

**Figure 3:**
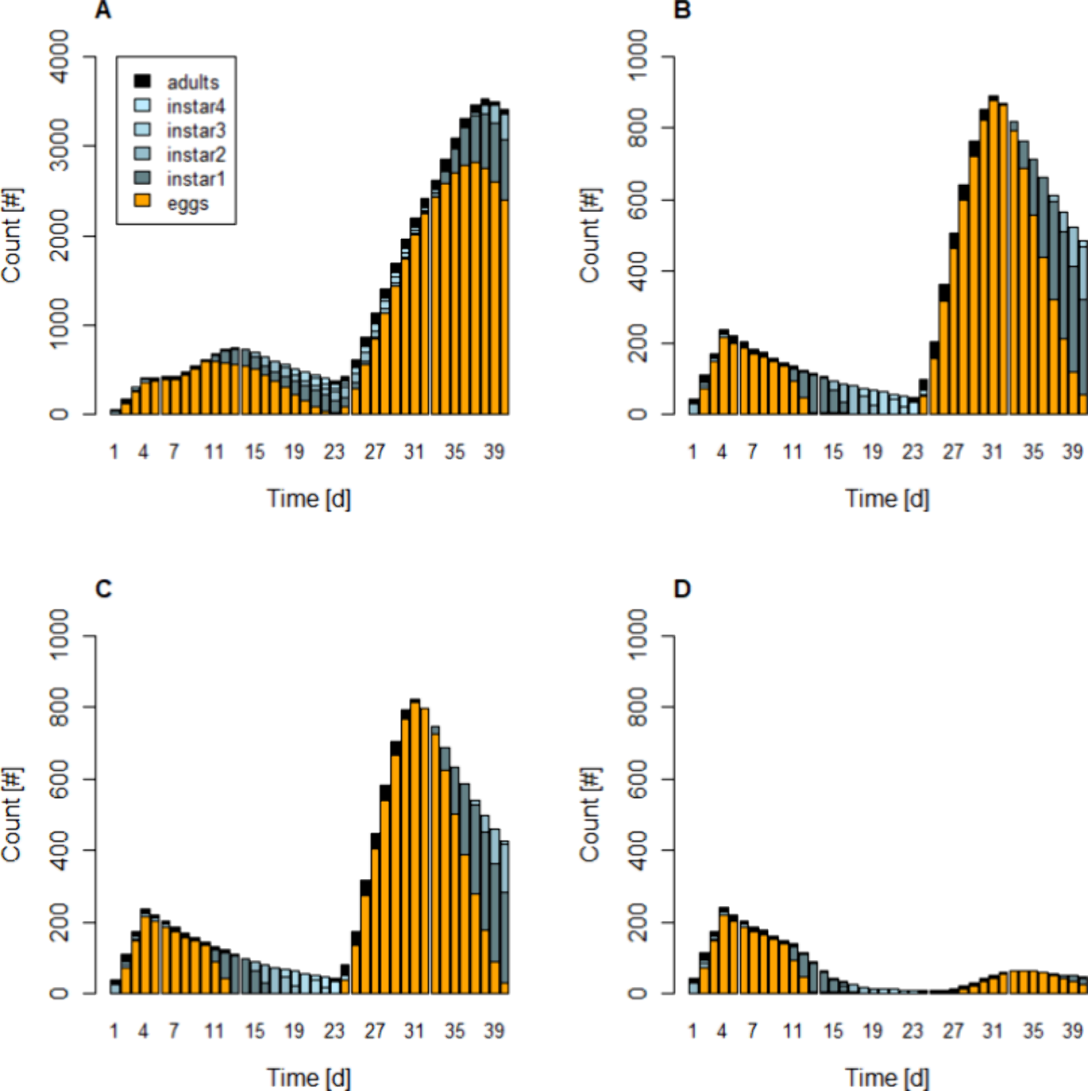
Simulated population demography for untreated control (A) and treated (B–D) whitefly populations at an ambient temperature of 30 °C. Different colors refer to immature and mature stages of *B. tabaci*. For the treatments, three different scenarios were simulated: (B) a single initial application of 12 g a.i.//ha spidoxamat, (C) two applications of 12 g a.i.//ha, initially and on day 6, and (D) two applications of 12 g a.i.//ha, initially and on day 12. For the initial population, we assumed a synchronized population of initially 100 2nd instar nymphs and two egg laying adults. Note the different scalings of the *y*-axis in control and treatments.

To explore the effect of temperature on different application intervals and the resulting efficacies in the next step, we utilized three distinct population settings. These settings resulted in medium to high whitefly abundance in untreated controls, but with varying population dynamics. The synchronized initial population, with adult migration excluded, exhibited fluctuating abundance due to the relatively uniform development of immature stages, background mortality reducing population size, and the subsequent egg-laying by emerging adults (Figures S2.18 and S2.19). In contrast, more heterogeneous initial populations combined with continuous immigration of untreated adults led to more consistent population increases (Figures S2.20–S2.23). The different population settings resulted in variations in predicted efficacy for various temperatures and application intervals, as illustrated in Figure 4, for spidoxamat application rates of 12 g a.i.//ha, with efficacy evaluated 14 days after the second application. Simulation results for the third scenario can be found in Figure S2.24. In the model simulations for the synchronized population scenario at temperatures below 28 °C, high efficacies were achieved regardless of the timing of the second application when assessing the entire population, including eggs, nymphs, and adults (Figure 4 A). However, when evaluating nymphs only, efficacy was higher for second applications later than 7 days after the initial treatment (Figure 4 B). At higher temperatures, a corridor for the second application between days 10 and 18 results in the highest efficacy, moving to later timings beyond 32 °C (Figure 3A, B). This pattern is a result of the interplay of temperature dependencies in both the host plant (Figure S2.3), which influences exposure, and whiteflies (Figure S2.26).

**Figure 4:**
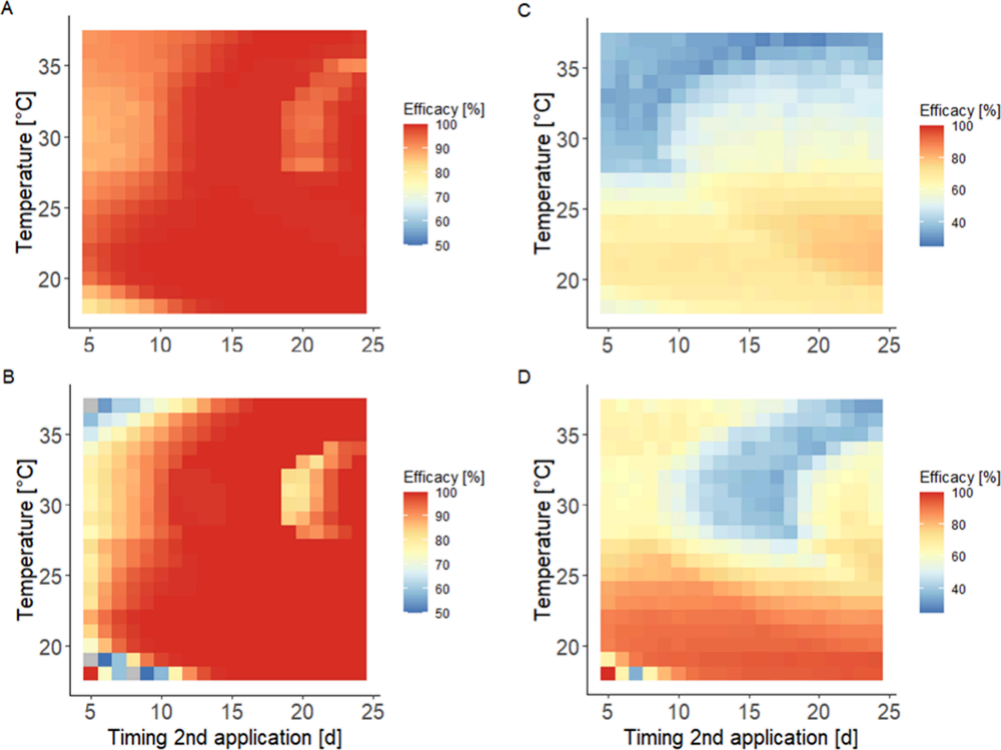
Predicted efficacy (color shades) for the **total population** (top, A and C, including mature and immature stages) and **nymphs** only (bottom, B and D) as a function of ambient temperature and timing of a second treatment and two different scenarios. Here, simulated efficacy was evaluated 14 days after the respective second application. Efficacy predictions are based on the median of 100 Monte Carlo simulations for two applications of 12 g a.i.//ha. For the two scenarios we assumed: (A,B) a **synchronized population** of initially 100 individual 2nd instar nymphs and two egg laying adults but no subsequent adult migration, and (C,D) a **more heterogeneous population** of initially 100 nymphs (structural length of 0.043 ± 0.01 cm) and a continuous low immigration rate (at 2 adults per day) and a default emigration rate of 50%. See Figures S2.16–S2.19 for exemplified simulation results for population trajectories. Note that the color scale differs between the left- and right-hand panel.

With the heterogeneity of the initial population and immigration rates of untreated adults being increased, the simulated efficacy of the 12 g a.i.//ha applications generally decreased (Figure 4), although the overall temperature dependency pattern remained consistent when evaluating the total whitefly population. Interestingly, when focusing solely on nymph efficacy, the previously described pattern was inverted: the corridor for the second application (days 10 to 18) resulted in lower efficacy. This can be attributed to the simultaneous presence of egg-laying females (sensitive to spidoxamat) and larger, less sensitive nymph stages during this period (Figure S2.21). A similar pattern, with further reduced efficacy, was observed with increased heterogeneity and migration rates (Figure S2.24). In these cases, higher application rates may achieve better efficacy (Figure S2.25), not necessarily increasing the effect at application but rather prolonging the exposure period above the threshold for effects.

## Discussion

In this study, we developed and successfully parametrized a TKTD model to evaluate the mortality of immature, spidoxamat-susceptible whitefly (*B. tabaci*) stages and the effects of spidoxamat exposure on fecundity and fertility in female adults. This TKTD model was then integrated into a DEB-IBM to predict population dynamics and efficacy under field conditions and validated with data from field trials conducted in India, Pakistan, and Brazil. The integrated model was utilized to identify optimal spidoxamat application strategies across various pest pressure scenarios and temperature regimes. Our findings indicate that, depending on ambient temperature and pest pressure, a second (backup) application can significantly enhance population control. The simulations suggested that the optimal timing for this second application is between 7 and 14 days post-initial application, influenced by the efficacy half-life and the developmental time of immature stages as a function of temperature.

The TKTD model effectively captured both the lethal effects on immature stages and the impact on adult reproduction despite applying the same dominant rate and threshold for lethal and sublethal effects and the apparent differences in the onset of the respective effects. The shared parameters, along with the slightly delayed onset of activity of the chemical, are overall in line with the lipid biosynthesis inhibiting mode of action of ketoenols.^53^ The differences in the onset of the lethal and sublethal effects, however, can to some extent be attributed to the experimental setup. In our assessments of larval mortality, the deposited eggs were treated 1 day prior to the predicted hatching of the nymphs. In addition, the translaminar exposure of nymphs begins only once the crawler stage has settled and commenced feeding. The duration of the crawler stage has been reported to vary between a few hours^54^ and somewhat longer.^55^ This variability can influence the timing of exposure and the subsequent lethality observed. Additionally, the approximately four-day development time of the first nymphal stage at 24 °C^11^ may further explain the delayed observation of lethal effects, as ketoenol-treated immature stages of insects and mites typically die when molting.^40^ In contrast, the rapid onset of reproductive effects suggests that adult whiteflies were quickly exposed to the chemical. The subsequent reduction in lipids in exposed adults likely led to decreased fecundity due to the species’ inherently high reproductive rate.^11^ It is also important to note that while lethal and sublethal effects differ in their estimated effect strengths, these distinctions become relevant only when the damage level exceeds the threshold required for an effect. Thus, the variability in the onset of these effects may not solely be explained by differences in the effect strength parameter but rather by the complex interactions of exposure timing, developmental stages, and physiological responses to the chemical treatment.

In our study, we did not observe any significant impact on body size or developmental delays in *B. tabaci* upon exposure to spidoxamat. Consequently, we focused on pMoAs that directly influence reproduction (see also ref 22). The fitted model utilizing the pMoA of ‘increased reproduction costs’ effectively described the observed reduction in the number of eggs laid by exposed adults. Typically, it is recommended to select a single pMoA to characterize sublethal effects based on the goodness of fit.^22^ However, in our analysis, we implemented a second pMoA, termed ‘hazard to embryos’, to simulate the subsequent decline in the number of fertile eggs produced. A similar assumption was previously adopted to model reduced reproduction and the production of empty cocoons in exposed earthworms.^24^ Note that in our data set, fecundity refers to the total number of eggs laid, regardless of their viability. We subsequently also assessed the fertility of these eggs and compared the number of viable offspring to the control group, which accounts for both the reduced number of eggs and the reduced viability in the treatment groups. Consequently, the pMoA ‘hazard to embryos’ encompasses both effects. In the IBM simulations, our focus is on the net reproduction and, thus, solely on the number of viable offspring added to the population count, eliminating the need to simulate the impact on the “cost of reproduction.” However, we utilized both pMoAs to leverage all available data for robust model parametrization.

While the DEB model allows simulation of individual growth and reproduction, other factors such as immigration, emigration, and biological interactions also play critical roles in regulating population growth and natural mortality.^7,12^ In our IBM implementation, we incorporated a background mortality rate for immature stages to account for various mortality mechanisms, including parasitism, predation, and dislodgement, as discussed by Naranjo and colleagues.^4,7^ IBMs typically include some form of density-dependent population regulation, such as competition or crowding effects, which can additionally increase mortality in a size- or stage-specific manner and/or reduce reproduction rates as a function of population density; refs 35,36,38 . We, however, chose to ignore density-dependence as our focus was on the control of early infestations of whitefly populations and the availability of host plants, allowing for a logarithmic population development.^56^ Generally, assessing density-dependent mortality factors in the field can be challenging, depending on the spatial scale of observations. Although Naranjo and Ellsworth^7^ were unable to demonstrate density dependence at the plant level for any mortality factors in their life tables for *B. tabaci*, they noted that the absence of evidence does not preclude its possible existence, as some factors may suppress populations for extended periods.

Our simulation results are consistent with the findings of Naranjo and Ellsworth,^7^ emphasizing the importance of immigration and emigration rates in regulating population dynamics in cotton. Typically, large numbers of adult whiteflies immigrate into cotton crops at the end of the cohost season and subsequently begin to emigrate as host quality declines in early fall.^12,56^ In contrast, the assumption of continuous or periodic immigration and emigration of adult whiteflies in our model simulations was essential for accurately capturing nymph counts in the untreated controls of the field trials. The frequent migration of adults assumed for those trials is likely attributable to the small plot sizes compared to full-scale agricultural fields, as well as the presence of untreated plots serving as source populations.

The integration of the TKTD model with the DEB-IBM in this study allows for a more nuanced understanding of the consequences of spidoxamat applications. Furthermore, the modeling approach can help identify optimal application rates and intervals for a given whitefly infestation that minimize environmental impact and delay the evolution of insecticide resistance, particularly when following the ‘Mode of Action (MoA) Treatment Windows’ approach.^5^ The basic aim of this approach is to avoid treating consecutive generations of whiteflies with insecticides of the same MoA. Multiple applications with insecticides of the same MoA are allowed within a generation ‘window’,^5^ which is in accordance with the simulations here, suggesting that the optimal timing for a second application is between 7 and 14 days post-initial application, depending on the ambient temperature and population structure. The findings of this study contribute significantly to the understanding and management of *B. tabaci* and underscore the potential of mechanistic models for exploring effective control strategies. Our modeling approach demonstrated that the timing and frequency of applications are critical for maximizing the efficacy, particularly under varying pest pressure scenarios and temperature regimes. This information is crucial for growers aiming to optimize pest management, while minimizing crop loss and ensuring sustainable production practices. The findings may be valid for *B. tabaci* biotype B populations on different crops only if life history traits of the species (for analysis, see ref 8) and spidoxamat bioavailability are comparable across host plants. Note, however, that the above considerations and model predictions are limited to whitefly populations fully susceptible to ketoenols (be reminded that TKTD parametrization was done based on data with the SUD-S strain) and are unlikely to hold true in situations where resistance alleles are present at varying frequencies.

The greenhouse trials conducted in this study serve as a critical bridge to extrapolating findings to field conditions. By validating the DEB-IBM against field trial data from diverse geographical locations, we enhance the reliability of our predictions and demonstrate the practical applicability of the model. This extrapolation is vital for ensuring that laboratory results translate effectively to real-world scenarios, thereby increasing the confidence of stakeholders in adopting modeled recommendations. Moreover, the insights gained from greenhouse experiments can inform future research directions, guiding the refinement of models and methodologies to address emerging challenges in pest management.

One of the key advantages of the DEB-IBM approach is its capacity to evaluate chemical effects and product recommendations beyond the traditional field trial data. The model allows for scenario analyses that consider a range of variables, including environmental conditions and pest population dynamics, which may not be fully captured in field studies. Also, the modular structure of the DEB-TKTD-based IBM allows extension to other species and chemicals without major changes in the model structure. This capability enables stakeholders to make more informed decisions regarding pest management practices, ensuring that recommendations are robust and adaptable to varying conditions.

In conclusion, the findings of this study highlight the multifaceted value of integrating individual-based modeling with empirical research in pest management. The presented modeling approach provides a comprehensive framework for understanding the intricate dynamics of *B. tabaci* populations. By simulation of individual-level interactions and responses to environmental variables, the model elucidates the factors driving population growth and decline. This understanding is crucial for developing targeted interventions that address the specific life stages and behaviors of pests, ultimately leading to more effective management strategies. By continuing to refine and apply such modeling approaches, we can enhance our understanding of pest dynamics and improve the efficacy of management strategies in an ever-evolving agricultural landscape.

## Supplementary Material







## Notes

Model description, including equations, and parameter estimates (PDF)
Additional results regarding TKTD parametrization and IBM validation, Overview on field trials and respective model settings, additional IBM predictions (PDF)
R code for executing TKTD models, including survival and reproduction data, IBM implementation in Delphi, IBM executable code in R (ZIP)
