# Supplementary material for: Optimizing Control Strategies for the Cotton Whitefly Bemisia tabaci: Insights from Individual-Based Modeling
Source: Environ Sci Technol. 2026 Jan 20;60(4):3036–45. doi: 10.1021/acs.est.5c13117 (PMC12874526; doi:10.1021/acs.est.5c13117)
Supplement: Supplementary file 1 [file es5c13117_si_001.pdf]

Supporting Information 1 to:

Optimizing control strategies for the cotton whitefly *Bemisia tabaci*: Insights from individual-based modeling

Andre Gergs[1*], Angelika Weinhold[1], Elena Hettmann[1], Mariana Durigan[2], Lokeshkumar Kadu[3], Jocelyn Kratchmer[1], Christian Marienhagen[1]

[1]Bayer AG, Alfred-Nobel Strasse 50, 40789 Monheim, Germany

[2] Bayer SA – Brasil, Avenida Doutor Roberto Moreira, 5005 Recanto dos Pássaros, 13148-914 Paulínia-SP, Brasil

[3] Bayer CropScience Limited, Bayer House, Central Avenue, Hiranandani Estate, Thane- 400 607. Maharashtra. India

*Corresponding author: andre.gergs@bayer.com

Supporting Information contains:

Number of pages: 9

Number of Tables: 2

Number of Figures: none

Model description

This documentation of the individual-based model (IBM) is structured according to the ODD (Overview, Design concepts, Details) protocol as outlined by Grimm et al. [1,2].

1. Purpose

The primary objective of this model is to analyze and predict the population dynamics of *Bemisia tabaci*, with a specific focus on optimizing pest management strategies.

2. Entities, State Variables, and Scales

The model operates at three hierarchical levels, corresponding to three distinct entities: ecosystem, population, and individual.

Ecosystem Level. This highest level represents a simplified ecosystem encompassing environmental variables such as host plant leaf area, ambient temperature, and chemical exposure concentration, which serve as input variables. The spatial scale covers a single plant, for now assuming homogeneous infestation of a cotton field. Food availability is assumed to be ad libitum; thus, the model does not currently account for plant damage or its effects on food quality and quantity.

Population Level. The population entity functions as a bookkeeping mechanism, monitoring population abundances through a single state variable, the population size. This population consists of numerous whitefly individuals, whose life cycles are modeled in detail. Changes in population size arise from individual birth and death events, as well as immigration and emigration of adults.

Individual Level. Each whitefly is represented through a dynamic energy budget (DEB) model[3], specifically utilizing the abp variant of the standard DEB model [e.g. 4]. Whitefly individuals are characterized by four primary state variables: The structural length influences overall energy turnover and maintenance costs. The reproduction buffer represents energy available for reproduction, which is converted into eggs during reproductive events. The damage stage connects chemical exposure concentrations to toxicodynamics, affecting survival and posing stress on life history traits. The vital state is based on the individual's survival probability. This state determines whether an individual is alive or dead, i.e. its inclusion in the population count. In addition to these primary state variables, several intermediate outputs are essential for updating the primary states. In standard DEB models and typified variations thereof, maturity regulates individual development stages. Since we assume a constant functional response of f=1, for simplicity, we do not explicitly simulate the reserve in the individual based model. Instead, we calculate the scaled catabolic flux directly from the scaled functional response and use fixed values for the structural length to determine stage transitions (see submodel section for further details).

3. Process Overview and Scheduling

The model operates in discrete time steps, updating state variables and intermediate outputs daily. The daily processes are executed in the following sequence: Rate constants are adjusted for ambient temperature. Toxicokinetics and toxicodynamics are computed based on exposure concentration. Food availability is considered constant; assimilation (energy uptake) occurs, converting assimilates into the reserve. A fraction of the reserve (κ) is allocated for somatic maintenance and growth, while the remaining ($1 - \kappa$) supports maturity maintenance and maturation. In mature whiteflies, net energy, after maintenance costs payment, is directed to the reproduction buffer, which in turn is converted into eggs. Upon reaching length at birth, embryos hatching from eggs and begin feeding. Each day, individual whiteflies face a probability of death, influenced by natural causes and toxicity-related damage. At the

end of each simulated day, dead individuals are removed, and newly laid eggs are added to the population count. Additionally, daily immigration and emigration rates, as specified in model settings, adjust the population size accordingly.

4. Design concepts

Emergence: Individual life history traits, along with population dynamics and structure, emerge from the metabolic organization of the individuals.

Adaptation: The model does not incorporate any adaptive processes.

Sensing: Individuals are sensing the ambient temperature and the exposure concentration.

Interactions: The model does not account for any interactions among individuals, including sexual reproduction which is not directly simulated.

Stochasticity: Two sources of stochasticity are integrated into the model. First, mortality is treated as a probabilistic process. For each individual, a survival probability is calculated based on the hazard rate during larval development, aging, and toxicokinetics-toxicodynamics. An individual dies if its survival probability exceeds a randomly generated number (drawn from a uniform distribution between 0 and 1) assigned at birth. The second source of stochasticity pertains to the initial composition of the population (refer to the initialization section) as well as immigration and emigration.

Observation: Throughout the simulation period, the total number of whiteflies and their abundance across various developmental stages—namely eggs, larval stages (N1-N4), and adults—are monitored. For Monte-Carlo simulations, the median and 95% prediction intervals are calculated for total abundance and abundance within each developmental stage, based on daily recorded values for each simulation run. Population counts can be expressed as total abundance (per plant) or as density, i.e., number of individuals per unit leaf area.

5. Initialization

The initialization of model simulations is predicated on the specific field conditions that they aim to replicate: The temperature trajectory (as detailed in the input data section) as well as the population size and the composition of the population at the start of the simulation needs to be specified.

For population initialization, each individual is randomly assigned a gender, assuming a 50/50 ratio of females to males. Additionally, individual body sizes are assigned based on the environmental scenario being simulated. Body sizes can be drawn from either a uniform distribution (ranging from egg to adult size) or a normal distribution, with the median set to the size of the major instar present in the population. The standard deviation for body size must also be specified in the model settings. Subsequently, for each individual, intermediate states such as instar, cumulative hazard, and the reproduction buffer (applicable to adults only) are calculated based on the assigned body size. This calculation assumes a scaled functional response of ($f = 1$), indicative of *ad libitum* food availability.

Depending on the environmental scenario under consideration, periods of adult immigration and emigration may be specified. For immigration, the rate at which adults are added to the population per day must be specified; adult individuals will be initialized as described above. For emigration, a percentage of adults will be randomly removed from the population, with a default value set at 50% per day, although this can be adjusted as necessary. In simulations extending over several month, adult emigration is considered if the plant reaches a certain size at the end of the growing season. As a threshold value for end-of-season emigration, we used a default of 22 mainstem nodes, reflecting a stage

where plant growth, in terms of internode length and leaf area, may have diminished compared to earlier growth stages [5, 6].

To simulate plant growth as a function of temperature (details available in the submodel section), the initial number of mainstem nodes or the initial leaf area must be specified, depending on the environmental scenario being modeled. For scenarios involving chemical exposure, the application rate(s) and timing of the application(s) need to be defined.

6. Input data

The daily mean temperature serves as the primary input data for the model, with the duration of the temperature trajectory dictating the length of the simulation. In scenarios involving chemical exposure, the daily exposure concentration is used as an input, calculated based on the specified application rates and timings, as well as the temperature-dependent exposure decline detailed in the submodel section below. While plant size is not directly utilized as an input in the model, it is employed for processing the model output when population densities need to be expressed per unit of leaf area.

7. Submodels

The submodels of the IBM include the DEB model for life history traits in *B. tabaci*, toxicokinetics and toxicodynamics, a model for host plant growth and chemical exposure dynamics, as well as a temperature correction function. Model equations (Eqns.) are listed in Table S1.1 while an overview on model parameters is provided in Table S1.2.

Reserve dynamics, growth and reproduction.

The standard Dynamic Energy Budget (DEB) model is classified within a category of typified models known as s-models. For a detailed description of the standard DEB model, see e.g. Jager et al. (2023). However, in most insects, growth during the nymphal stage is characterized by rapid and typically exponential development. In DEB theory, this growth pattern is referred to as a V1-morph. Those that exhibit metabolic acceleration during part of their life cycle are categorized as a-models. Within this classification, the “abp” subtype of models features metabolic acceleration from birth until puberty, after which growth halts [7]. In this context, birth coincides with hatching, while puberty aligns with emergence. The adult stage is characterized by a complete cessation of growth.

In the abp model, V1-morphy pertains solely to the relationship between surface area and structural volume; thus, changes in shape influence only the specific maximum assimilation rate $\{\dot{p}_{Am}\}$ and energy conductance $\dot{v}$. These parameters are adjusted by an acceleration factor, which, for this model, is based on the structural lengths at birth (L_b) and puberty (L_p), see Eqn. 1. The specific maximum assimilation rate and energy conductance before acceleration are denoted p_{Am0} and υ_0 respectively. Since we assume a constant food environment the mobilization flux S_c of assimilates is directly calculated from the scaled functional response, here f=1 for *ad libitum* conditions (Eqn. 2).

A fraction of the mobilization flux is used for growth. The change in structural length is assumed to until reaching the size at puberty L_p, as formulated in Eqn. 3. However, the growth of immature insects, in terms of the physical length, typically occurs through a series of distinct developmental stages, i.e. larval instars. Dyar’s Law posits that the ratio of lengths at two consecutive molts remains constant, which allows to define stage transitions based on stress parameters for each of the instars (s_1-s_3). The size of a nymph after molt L_2 can be calculated from the size of the current instar L_1 as $L_2 = s_1^{½} L_1$, and so on. Another part of the mobilization flux is used for maturation (Eqn. 4), which is calculated only for completeness, but remains unused as we work with length triggers for stage transitions. A key assumption within DEB theory is the κ-rule, which states that a fraction κ of mobilized energy is

allocated for somatic maintenance and growth, while the remaining fraction ($1 - \kappa$) is allocated for maturity maintenance (see above) and reproduction following puberty. However, in the case of abp models, where growth ceases after puberty, questions arise regarding energy allocation during the adult stage. The assumption is thus that the κ-rule does not operate in adults. After reaching puberty, which coincides with the imago stage of the white flies the reproduction rate follows from Eqn. 5. The individual survival probability is calculated based on a background hazard rate (Eqn. 6) and TKTD formulation for chemical effects (see TKTD section below, Eqn. 11) in nymphs and hazard due to aging in adults (Eqns. 7 & 8). The aging module introduces two additional parameters: the Weibull aging acceleration (h_a) and the Gompertz stress coefficient (s_G). Note, that some equations require the calculation of the compound parameter g (Eqn. 9)

TKTD for survival and stress on reproduction

We focus on the reduced TKTD model, where toxicokinetics and damage dynamics are integrated into a single compartment (Eqn. 10). A full TKTD module would utilize separate compartments, which typically necessitates the measurement of body residues for calibration. It is crucial to emphasize that the single damage compartment in the reduced module does not inherently correspond to an internal concentration of the toxicant. Depending on the relative rates of toxicokinetics and damage dynamics, this compartment may either reflect an internal concentration or signify a form of damage. Accordingly, the dominant rate constant k_d represents either a toxicokinetic or a toxicodynamic process. Also note that the damage is scaled with the exposure concentration and has, thus, the unit of the application rate of the chemical.

In this model, we limit our consideration to translaminar exposure, whereby chemical uptake and damage accumulation occur exclusively through feeding. Non-feeding instars, specifically the egg and pseudo-pupa stages, are assumed to be unexposed to the chemical. Observations indicated minimal effects in the large nymph stage preceding the pseudo-pupa, leading us to exclude this stage from exposure in the IBM implementation too. Nonetheless, all modelled developmental stages including adults can repair damage.

Both sub-lethal and lethal effects are incorporated into the model, with the assumption that both are influenced by the same damage level by default. For lethal effects, damage influences the hazard rate, adhering to a linear-with-threshold relationship (Eqn. 11). For sub-lethal effects, damage impacts one or more primary parameters of the energy budget model, via a stress function F_s (Eqn. 12), also following a linear-with-threshold relationship. We assume that lethal and sub-lethal effects share the same threshold parameter z but differ in their effect strength b_s and b_b respectively.

Fecundity effects in adults are simulated as increased reproduction costs (Eqn.13) and fertility effects are represented by the physiological mode of action of hazard to embryo (Eqn. 14). For further information on the concept see e.g. Jager et al.[8]. Note, that in the IBM we only simulate the number of viable offspring based on the formulation for hazard to embryos.

Host plant growth and chemical exposure

We employ a simplified model for plant growth, wherein the increase in leaf area A and the number of mainstem nodes N are determined by their respective growth rates, r_A and r_N, as specified in Eqns. 15 and 16. This linear growth assumption is deemed reasonable for the majority of the cotton growth season (Reddy et al. 2002). Note, however, that the continuous growth in terms of mainstem nodes is only a rough approximation and lacks realisms as only full mainstem nodes are produced.

For modeling the translaminar exposure of whiteflies, we utilize a one-compartment model analogous to the aforementioned TKTD model for whiteflies. In this model, the internal concentration is scaled

with the external application rate and plant uptake occurs instantaneously. Note, however, that we simulated cuticle penetration of the chemical to quantify the role of water volume during field applications (Eqn. 17). For IBM simulations, we therefore calculated the penetration after 24 h for a given water volume relative to a maximum of 300L. This fraction is then multiplied with the actual application rate to derive the initial scaled internal concentration for the simulation. The scaled internal concentration subsequently decreases over time due to metabolic processes and dilution effects resulting from plant growth. Rather than explicitly simulating the underlying mechanisms, we adopt a one-parameter model characterized by the rate constant k, which represents the dominant process. The exposure concentration C is then calculated based on Eqn. 18.

Temperature correction

The Arrhenius function (Eqns. 19-22) is employed to account for the effects of temperature on metabolic processes. The simplest formulation of this function utilizes a single parameter, the Arrhenius temperature T_A. This function introduces a temperature correction factor that quantifies how a biological rate at a given temperature T compares to that at a reference temperature T_{ref} (Kooijman, 2010). For temperatures approaching the thermal limits of a species, the Arrhenius function must be extended to consider the reduction of metabolic rates at both low T_L and high T_H extremes of the thermal tolerance range. This extension incorporates species-specific Arrhenius temperatures (T_{AL} and T_{AH}), which govern the rate of decline at these boundaries. The temperature correction is employed for both whiteflies and host plant, with different parameter sets for the two species. Note however, that only the upper thermal limit was considered for the host plant dynamics, due to lack of temperature data for the lower extreme. Furthermore, we assumed a lower T_H value for the temperature correction of the hazard rate of juvenile whiteflies as suggested by data.

All model rate constants are adjusted for temperature using the derived temperature correction factor, ensuring that the effects of temperature on metabolic and other processes are represented within the model.

8. Implementation

The model is implemented in Delphi 12 (Embarcadero Technologies Inc., 2024) and is based on discretized forms of the differential equations listed in Table S1.1.

Table S1.1: Model equations for life history traits in white flies, toxicokinetics and toxicodynamics, chemical exposure, temperature correction and cotton growth.

Equations	*No.*
Acceleration, mobilization flux, growth and reproduction	
$s_M = \max(L_b\,, L)\; L_p^{-1}\;;\; \dot{v} \rightarrow \;\dot{v}\; s_M\;;\; \{\dot{p}_{Am}\} \rightarrow \{\dot{p}_{Am}\}\; s_M$	*Eqn. 1*
$S_c = \; L^2\, g\, f\, \left(1 + \frac{[\dot{p}_M]}{[E_G]}\; L\;\; \dot{v}\right)\; (g+f)^{-1}$	*Eqn. 2*
$\frac{d}{dt} L = \; \frac{1}{3}\; L^{-2} \left(\frac{\dot{v}}{g}\;\; S_c - \; \frac{[\dot{p}_M]}{[E_G]}\; L^3\right)\;\; for\; L < \; L_p$	*Eqn. 3*
$\frac{d}{dt} E_H = \{\dot{p}_{Am}\}\; (1-\kappa)\; S_c - \; \dot{k}_J\;\; E_H \qquad for\; L < \; L_p$	*Eqn. 4*
$\frac{d}{dt} R = \kappa_R \big(f\{\dot{p}_{Am}\} L_p^2 - \; [\dot{p}_M]\; L_p^3 - \; \dot{k}_J\; E_H^p \;\; \big)\; E_0^{-1}\;\; for\; L = \; L_p$	*Eqn. 5*
Hazard during nymph development (L < L_p)	
$\frac{d}{dt} H = \; -\dot{h}_b$	*Eqn. 6*
Hazard due to aging (L = L_p)	
$h_w^3 = \; 6\; \ddot{h}_a \dot{v}\; L_p^{-1}\;\; ;\;\; h_G = \; s_G \dot{v}\; L_p^{-1}$	*Eqn. 7*
$S(t) = exp\left(\frac{6\; h_w^3}{h_G^3}\; (1 - \exp(h_G) + h_G + \frac{h_G^2}{2})\right)$	*Eqn. 8*
Compound parameters	
$g = \frac{[E_G]}{\kappa\, E_m}\;\;\; with\; E_m = \frac{\{\dot{p}_{Am}\}}{\dot{v}}$	
Toxicokinetics and toxicodynamics	
$\frac{d}{dt} D = \; k_d (C - D)$	*Eqn. 10*
$\frac{d}{dt} H = \; b_w\; max(D - z, 0)$	*Eqn. 11*
$F_s = \; b_b\; max(D - z, 0)$	*Eqn. 12*
$\kappa_R \rightarrow \; \kappa_R (1 + F_s)^{-1}$	*Eqn. 13*
$\kappa_R \rightarrow \; \kappa_R\; exp(-F_s)$	*Eqn. 14*
Host plant growth and chemical exposure	
$\frac{d}{dt} N = r_N$	*Eqn. 15*
$\frac{d}{dt} A = r_A$	*Eqn. 16*
$\frac{d}{dt} C = \; -k_e\; (\kappa_{iw}\; f_V\; V\; C_e - C)$	*Eqn. 17*
$\frac{d}{dt} C = \; -k\; C$	*Eqn. 18*

Table S1.1: Model equations continued

Equations	*No.*
Temperature correction	
$s_A = \exp\left(\frac{T_A}{T_{ref}} - \frac{T_A}{T}\right)$	*Eqn. 19*
$s_L = \left(1 + \exp\left(\frac{T_{AL}}{T_{ref}} - \frac{T_{AL}}{T_L}\right)\right)\left(1 + \exp\left(\frac{T_{AL}}{T} - \frac{T_{AL}}{T_L}\right)\right)^{-1}$	*Eqn. 20*
$s_H = \left(1 + \exp\left(\frac{T_{AH}}{T_H} - \frac{T_{AH}}{T_{ref}}\right)\right)\left(1 + \exp\left(\frac{T_{AH}}{T_H} - \frac{T_{AH}}{T}\right)\right)^{-1}$	*Eqn. 21*
$F_T = s_A\, s_L \;\; for\, T \le T_{ref}\; ; \;\; F_T = s_A\, s_H \;\; for\, T > T_{ref}$	*Eqn. 22*

Table S1.2: Model parameters at reference temperature T_{ref} = 20°C. DEB parameters were available online [9].

Symbol	Value	Unit	Description
DEB parameters and implied properties for B. tabacci			
$\{\dot{p}_{Am}\}$	12.829	J/d.cm^2	Spec max assimilation rate
$\dot{v}$	0.002947	cm/d	Energy conductance
κ	0.9999	-	Allocation fraction to soma
κ_R	0.95		Reproduction efficiency
$[\dot{p}_M]$	40.53	J/d.cm^3	Vol-spec somatic maintenance
$\dot{k}_J$	0.02	1/d	Maturity maintenance rate coefficient
$[E_G]$	4423	J/cm^3	Spec cost for structure
E_H^b	1.912e-07	J	Maturity at birth
E_H^p	6.826e-06	J	Maturity at puberty
$\ddot{h}_a$	0.1968	1/d^2	Weibull aging acceleration
$\dot{h}_b$	0.01963	1/d	Juvenile hazard rate
s_G	0.0001	-	Gompertz stress coefficient
T_A	5805	K	Arrhenius temperature
T_{AH}	30000	K	Arrhenius temperature at upper boundary
T_{AL}	17330	K	Arrhenius temperature at lower boundary
T_H	308.1	K	Upper boundary temperature
T_{Hs}	301.4	K	Upper boundary temperature juvenile hazard rate
T_L	293.1	K	Lower boundary temperature
s_1	2.548	-	Stress at instar 1
s_2	1.58	-	Stress at instar 2
s_3	1.853	-	Stress at instar 3
E_0	0.00377	J	Initial reserve
L_b	0.00751	cm	Structural length at birth
L_p	0.0246	cm	Structural length at puberty

Table S1.2: continued. Model parameters at reference temperature T_{ref} = 20°C.

symbol	value	units	description
Toxicokinetic-toxicodynamics			
k_d	0.085	1/d	Dominant rate constant
z	0.004	g/ha	Threshold for effect
b_w	0.25	ha/g/d	Effect strength lethal
b_b	9.48	ha/g/d	Effect strength sublethal
Host plant and exposure			
r_N	0.26	1/d	Growth rate mainstem nodes
r_A	2.04	1/d	Growth rate mainstem leaf area
k	0.042	1/d	Exposure decay rate
k_e	0.198	1/d	Penetration rate
κ_{iw}	0.276	-	relative partition coefficient for penetration
f_V	1.43	1/L	Water volume correction factor
T_A	3.336e+04	K	Arrhenius temperature
T_{AH}	4.392e+04	K	Arrhenius temperature at upper boundary
T_H	301.6	K	Upper boundary temperature

References

1. Grimm, V.; Berger, U.; Bastiansen, F.; Eliassen, S.; Ginot, V.; et al. A Standard Protocol for Describing Individual-Based and Agent-Based Models. *Ecol. Model.* **2006**, *198*, 115–126.
2. Grimm, V.; Berger, U.; DeAngelis, D. L.; Polhill, J. G.; Giske, J.; et al. The ODD Protocol: A Review and First Update. *Ecol. Model.* **2010**, *221*, 2760–2768.
3. Kooijman, S. A. L. M. *Dynamic Energy Budget Theory for Metabolic Organisation*, 3rd ed.; Cambridge University Press: Cambridge, UK, **2010**.
4. Klagkou, E.; Gergs, A.; Baden, C. U.; Lika, K. Dynamic Energy Budget Approach for Modeling Growth and Reproduction of Neotropical Stink Bugs. *Ecol. Model.* **2024**, *493*, 110740.
5. Reddy, K. R.; Boone, M. L.; Hodges, H. F.; McKinion, J. M. Simulating Cotton Plant Height and Leaf Area Development. In *Proceedings of the Beltwide Cotton Conference* **1996**, 1, 532–538.
6. Reddy, K. R.; Kakani, V.; McKinion, J. M.; Baker, D. Applications of a Cotton Simulation Model, GOSSYM, for Crop Management, Economic, and Policy Decisions. In *Agricultural System Models in Field Research and Technology Transfer*; Ahuja, L. R., Ma, L., Howell, T. A., Eds.; CRC Press, LLC: Boca Raton, FL, USA, **2002**, 33–54.
7. Marques, G.; Augustine, S.; Lika, K.; Pecquerie, L.; Domingos, T.; Kooijman, S. A. L. M. The AmP Project: Comparing Species on the Basis of Dynamic Energy Budget Parameters. *PLoS Comput. Biol.* **2018**, *14*, e1006100.
8. Jager, T.; Goussen, B.; Gergs, A. Using the Standard DEB Animal Model for Toxicokinetic-Toxicodynamic Analysis. *Ecol. Model.* **2023**, *475*, 110187.
9. Gergs, A. AmP *Bemisia tabaci*, Version 2024/03/19. **2024**. Available online at: https://www.bio.vu.nl/thb/deb/deblab/add_my_pet/entries_web/Bemisia_tabaci/Bemisia_tabaci_res.html
