# Supplementary material for: Optimizing Control Strategies for the Cotton Whitefly Bemisia tabaci: Insights from Individual-Based Modeling
Source: Environ Sci Technol. 2026 Jan 20;60(4):3036–45. doi: 10.1021/acs.est.5c13117 (PMC12874526; doi:10.1021/acs.est.5c13117)
Supplement: Supplementary file 2 [file es5c13117_si_002.pdf]

Supporting Information 2 to:

Optimizing control strategies for the cotton whitefly *Bemisia tabaci*: Insights from individual-based modeling

Andre Gergs[1*], Angelika Weinhold[1], Elena Hettmann[1], Mariana Durigan[2], Lokeshkumar Kadu[3], Jocelyn Kratchmer[1], Christian Marienhagen[1]

[1]Bayer AG, Alfred-Nobel Strasse 50, 40789 Monheim, Germany

[2] Bayer SA – Brasil, Avenida Doutor Roberto Moreira, 5005 Recanto dos Pássaros, 13148-914 Paulínia-SP, Brasil

[3] Bayer CropScience Limited, Bayer House, Central Avenue, Hiranandani Estate, Thane- 400 607. Maharashtra. India

*Corresponding author: andre.gergs@bayer.com

Supporting Information contains:

Number of pages: 24

Number of Tables: 2

Number of Figures: 26

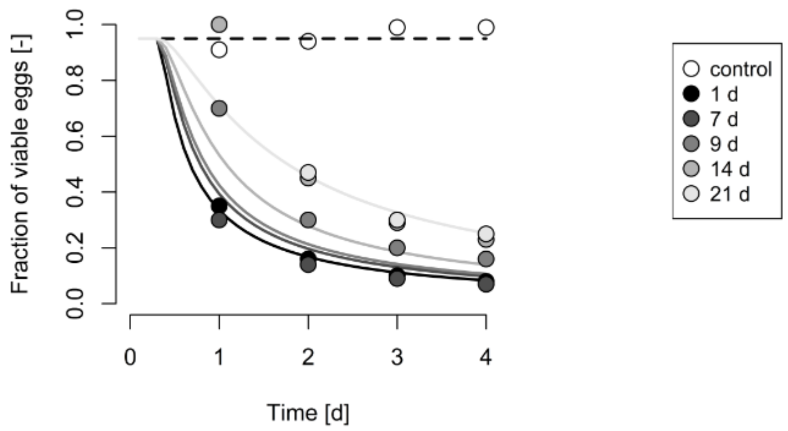

Figure S2.1: Fraction of viable eggs in adult whiteflies as function of time quantified on plants aged for 1, 7, 9, 14 and 21 days after initial spidoxamat exposure at 24 g/ha. Dots and lines represent data and model fit respectively. Code and data is available from Supplementary Information 3.

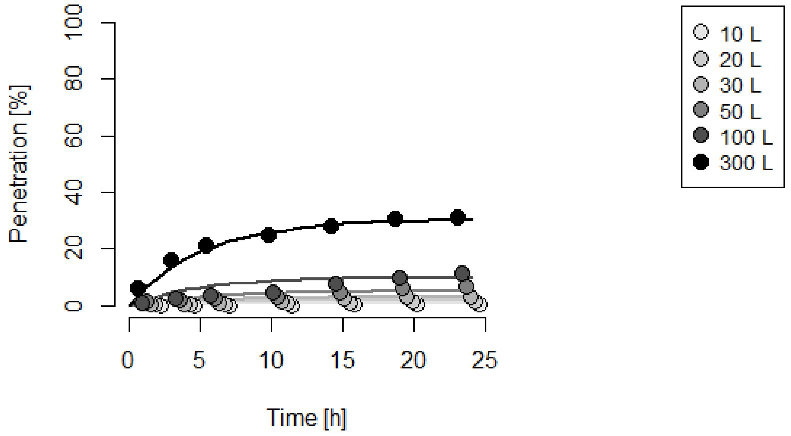

Figure S2.2: Cuticle penetration as percentage of applied spidoxamat for different water volumes in terms of L/ha.

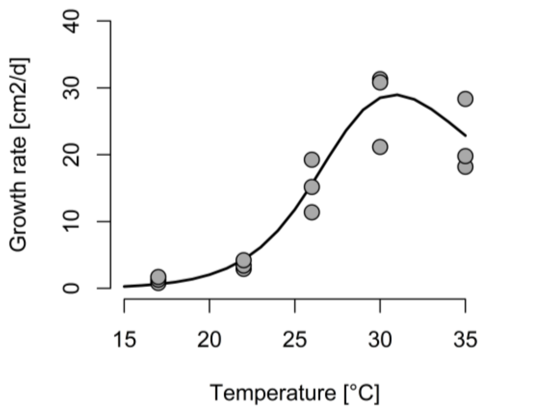

Figure S2.3: Growth rate in terms of cotton leaf area as function of temperature. Dots and line represent measured data and model fit respectively. Data was derived from the dissertation by Brand [1].

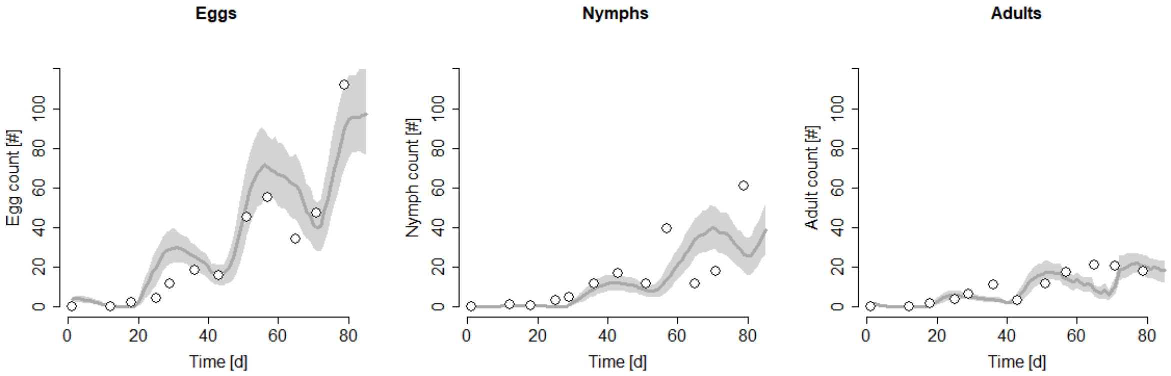

Figure S2.4: Model validation for population dynamics of immature and adult stages of *Bemisia tabaci* in cotton. USA. Data (dots) from **1997**, Maricopa, Arizona was extracted form Naranjo and Ellsworth (Fig. 2) [2]. The timing of adult immigration for the simulations was set in accordance with the observation[2] (Fig. 4). We additionally assumed fraction of emigrating adults 0.95 once the simulated host plant height exceeded 22 mainstem nodes (seedling size was assumed as initial value), which is used as a start of emigration in the model settings. Solid line and shade represent the median and 95% prediction intervals of 100 Monte-Carlo simulation runs. As indicated in the original publication, weather data for the study is available online from https://ag.arizona.edu/azmet/06.htm. In the original study, egg and nymph counts relate to sampled leaf discs, which was mimicked in our simulations. Adult counts per leaf were provided by the authors [2]. As the actual leaf area was unknown (and may vary over time) we assumed a 20 x larger area by default for adult counts in our simulations.

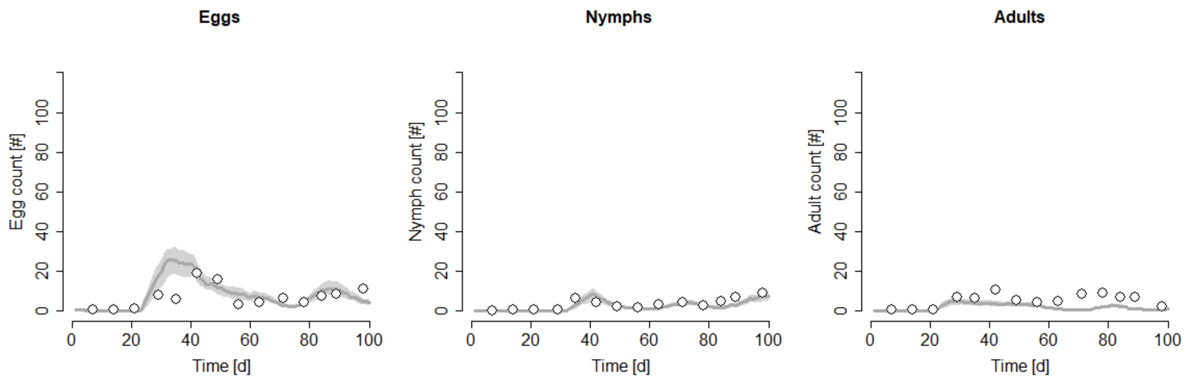

Figure S2.5: Model validation for population dynamics of immature and adult stages of *Bemisia tabaci* in cotton. Data (dots) from **1998**, Maricopa, Arizona was extracted from Naranjo and Ellsworth (Fig. 2)[2]. The timing of adult immigration for the simulations was set in accordance with the observation[2] (Fig. 4). We additionally assumed fraction of emigrating adults 0.95 once the simulated host plant height exceeded 22 mainstem nodes (seedling size was assumed as initial value), which is used as a start of emigration in the model settings. The authors observed higher than usual immature mortality in early August 1998. To account for this observation, we increased the background hazard rate by a factor 2 between day 40 and day 60 of the simulation. Solid line and shade represent the median and 95% prediction intervals of 100 Monte-Carlo simulation runs. As indicated in the original publication, weather data for the study is available online from https://ag.arizona.edu/azmet/06.htm. In the original study, egg and nymph counts relate to sampled leaf discs, which was mimicked in our simulations. Adult counts per leaf were provided by the authors[2]. As the actual leaf area was unknown (and may vary over time) we assumed a 20 x larger area by default for adult counts in our simulations.

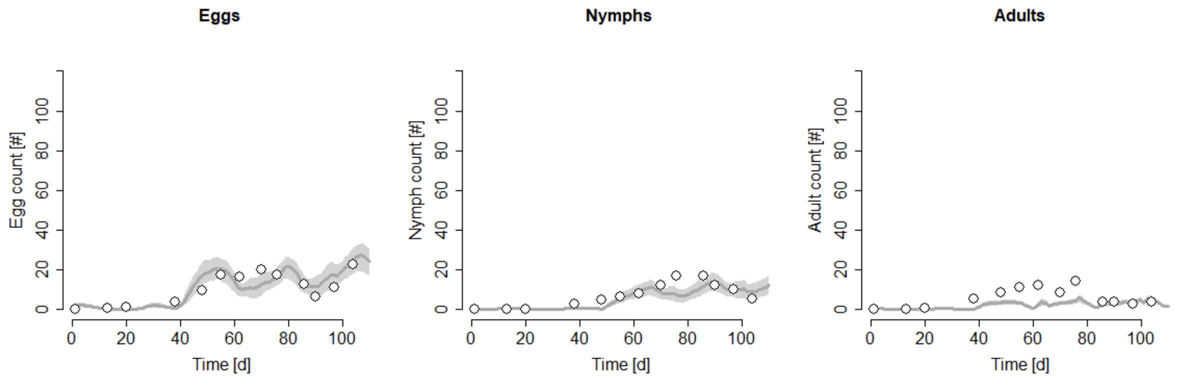

Figure S2.6: Model validation for population dynamics of immature and adult stages of *Bemisia tabaci* in cotton. Data (dots) from **1999**, Maricopa, Arizona was extracted from Naranjo and Ellsworth (Fig. 2)[2]. The timing of adult immigration for the simulations was set in accordance with the observation[2] (Fig. 4). We additionally assumed fraction of emigrating adults 0.95 once the simulated host plant height exceeded 22 mainstem nodes (seedling size was assumed as initial value), which is used as a start of emigration in the model settings. Solid line and shade represent the median and 95% prediction intervals of 100 Monte-Carlo simulation runs. As indicated in the original publication, weather data for the study is available online from https://ag.arizona.edu/azmet/06.htm. In the original study, egg and nymph counts relate to sampled leaf discs, which was mimicked in our simulations. Adult counts per leaf were provided by the authors [2]. As the actual leaf area was unknown (and may vary over time) we assumed a 20 x larger area by default for adult counts in our simulations.

Table S2.1: Overview on field trials, treatment information includes application rate (a.i. g/ha) and timing (days) of the applications.

Trial No.	Location	Start date	Plots [#]	Plot size [m^2]	Sampled leaves [#/plot]	Adjuvant system	Water volume [L]	Treatment A	Treatment B	Treatment C
1	Trindade, Goiás, Brazil	2018/05/7	3	21,6	10	Aureo	n.a.	2 x 18 g/ha day 1, day 8	2 x 24 g/ha day 1, day 8	2 x 48 g/ha day 1, day 8
2	Campo Verde, Mato Grosso, Brazil	23/04/2024	4	30	10	Aureo	150	3 x 12 g/ha day 1, day 10, day 21	3 x 16.8 g/ha day 1, day 10, day 21	3 x 24 g/ha day 1, day 10, day 21
3	Sorriso, Mato Grosso, Brazil	29/04/2024	4	20	10	Aureo	150	3 x 12 g/ha day 1, day 10, day 21	3 x 16.8 g/ha day 1, day 10, day 21	3 x 24 g/ha, day 1, day 10, day 21
4	Barreiras, Bahia, Brazil	29/04/2024	4	20	10	Aureo	150	3 x 12 g/ha day 1, day 10, day 21	3 x 16.8 g/ha day 1, day 10, day 21	3 x 24 g/ha day 1, day 10, day 21
5	Rahim Yar Khan, Punjab, Pakistan	2024/06/26	3	20	10	RME 500 EW	350	1 x 24 g/ha day 1	2 x 24 g/ha day 1, day 8	2 x 24 g/ha day 1, day 15
6	Zareef Shaheed, Punjab, Pakistan	2024/06/26	3	25	10	RME 500 EW	350	1 x 24 g/ha day 1	2 x 24 g/ha day 1, day 8	2 x 24 g/ha day 1, day 15
7	Sahiwal, Punjab, Pakistan	2024/07/26	3	20	10	RME 500 EW	500	1 x 24 g/ha day 1	2 x 24 g/ha day 1, day 8	2 x 24 g/ha day 1, day 15
8	Gehri Devi Nagar, Punjab, India	2024/09/07	3	20	30	RME 500 EW	500	1 x 24 g/ha day 1	2 x 24 g/ha day 1, day 8	2 x 24 g/ha day 1, day 15
9	Hisar, Haryana, India	2024/09/09	3	20	30	RME 500 EW	500	1 x 24 g/ha day 1	2 x 24 g/ha day 1, day 8	2 x 24 g/ha day 1, day 15

Table S2.2: Overview on model settings for the simulations of field trials

**Description**	**Trial 1**	**Trial 2**	**Trial 3**	**Trial 4**	**Trial 5**	**Trial 6**	**Trial 7**	**Trial 8**	**Trial 9**
General settings									
Monte-Carlo runs, #	100	100	100	100	100	100	100	100	100
Simulation time until 1st application, d	1	1	7	1	13	13	10	10	9
Factor population size	4	9	12	10	10	10	25	1	1
Initial population setting									
Stage distribution, 0/1 (uniform/normal)	0	1	1	1	0	1	1	1	1
Mean initial life stage, 0 - 5 (egg to adult)	*NA*	1	0	1	*NA*	5	3	3	3
Standard deviation for structural length, cm	*NA*	0.0085	0.0015	0.01	*NA*	0.02	0.1	0.012	0.01
Initial population size, #	200	450	160	140	4	10	37	120	65
Adult Migration									
Start of immigration, d	0	4	10	0	2	3	5	7	7
End of immigration, d	23	20	24	43	40	35	41	40	39
Immigration rate, #/d	2	4	1	5	2	7	2	15	6
Start of emigration, d	0	0	0	0	0	0	0	5	5
End of emigration, d	23	45	27	43	31	35	41	40	39
Fraction of emigrating adults	0.5	0.97	0.95	0.8	0.5	0.95	0.25	0.57	0.57

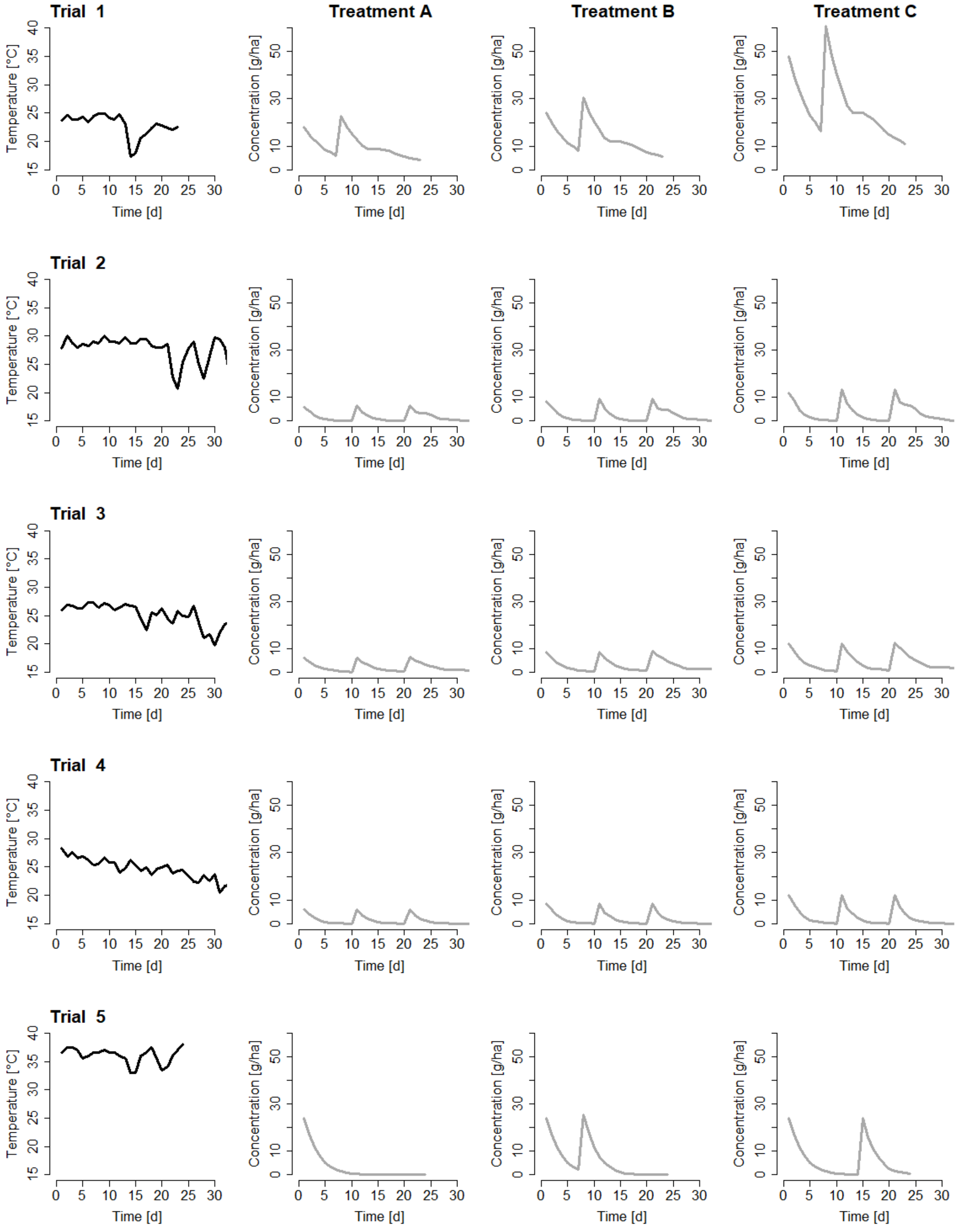

Figure S2.7: Mean measured temperature and calculated exposure scenarios for each of the field trials (for an overview see Table S2.1) as input data for the individual-based population model.

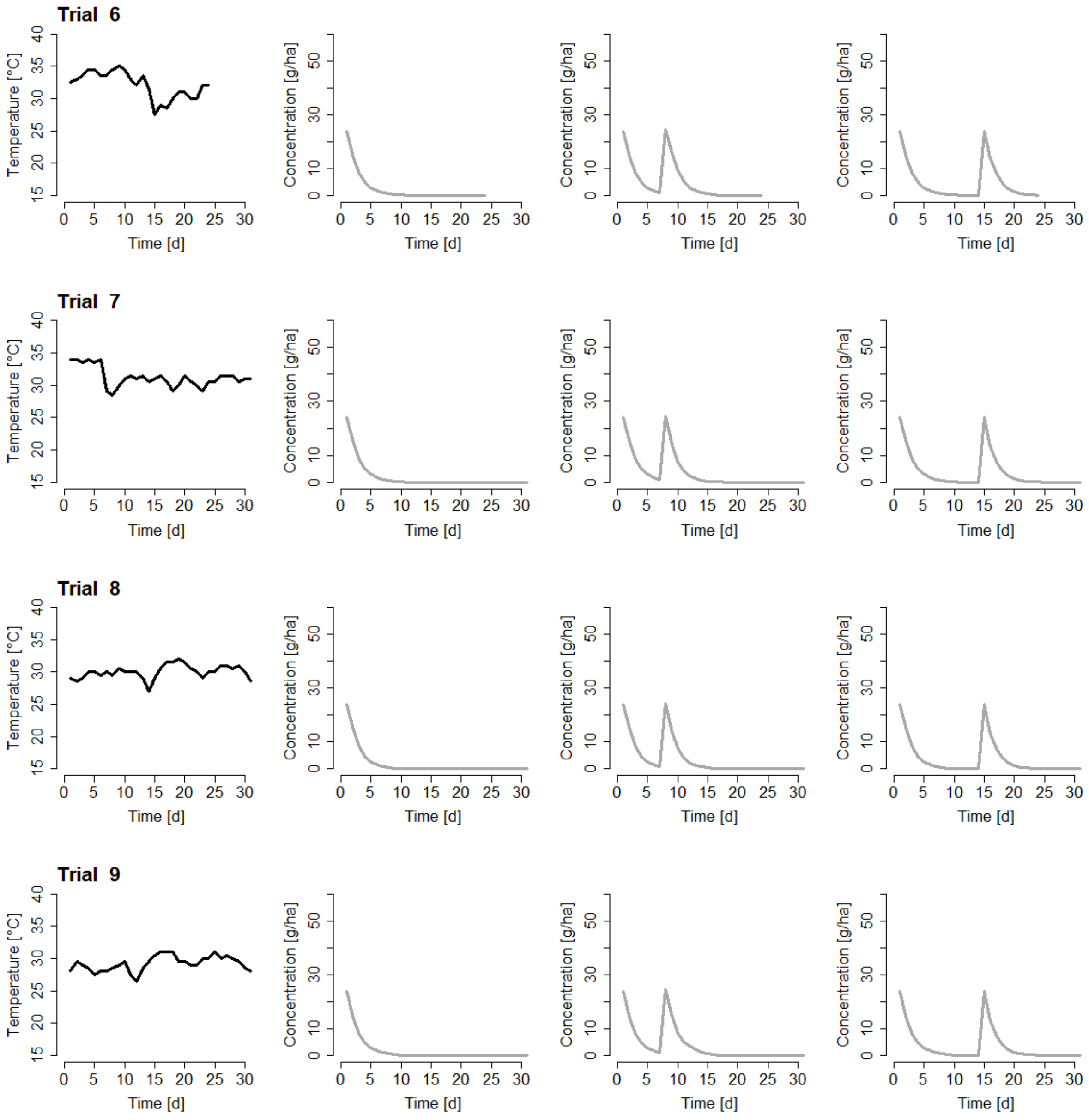

Figure S2.7 continued.

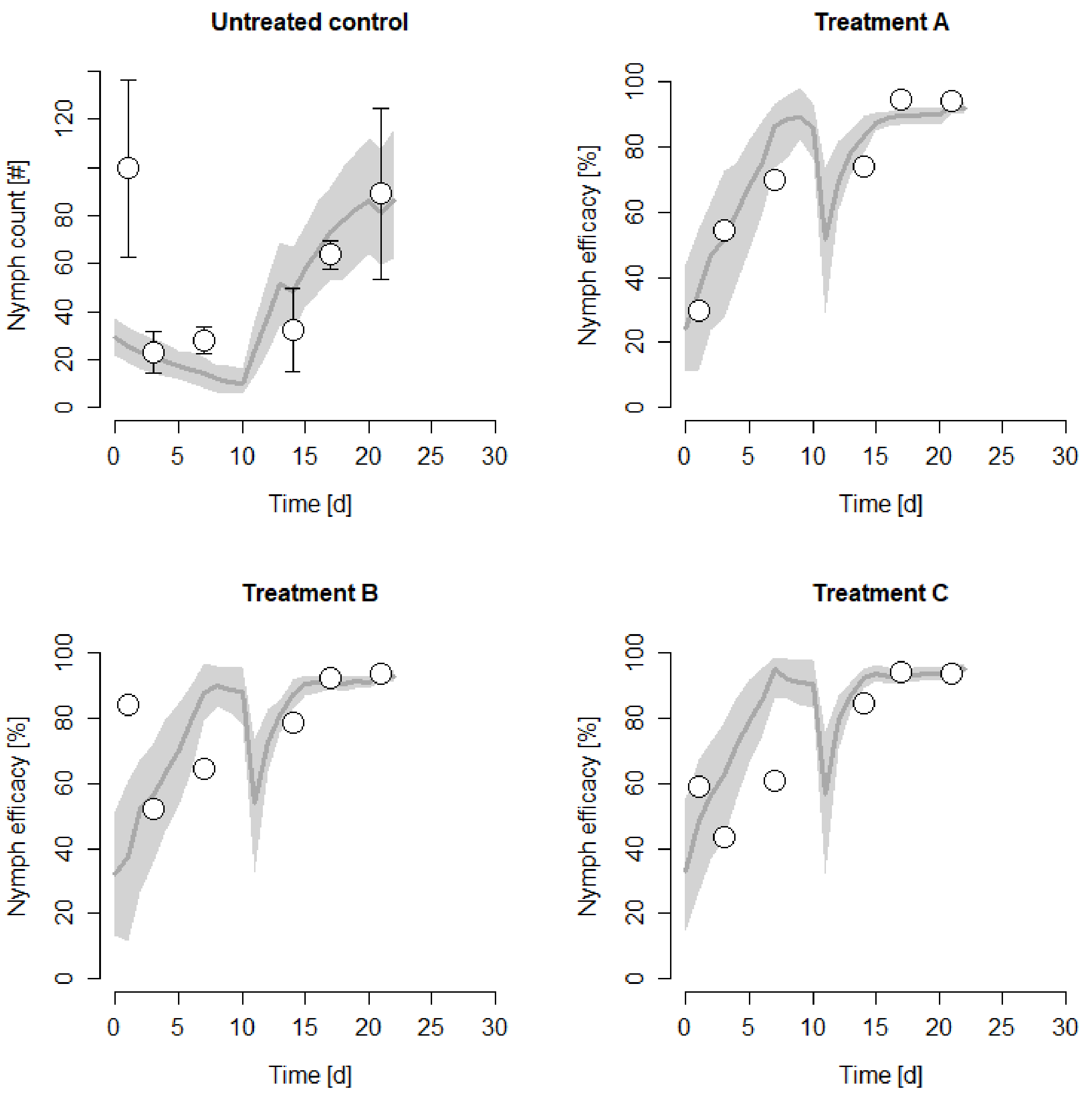

Figure S2.8: Nymph count and efficacy quantified in **Field Trial 1** (for details see Table S2.1). Dots are measured data (means and standard deviation for counts and means for efficacy based on counts) while the lines and grey shades represent the median and 95% prediction intervals of 100 Monte-Carlo simulations.

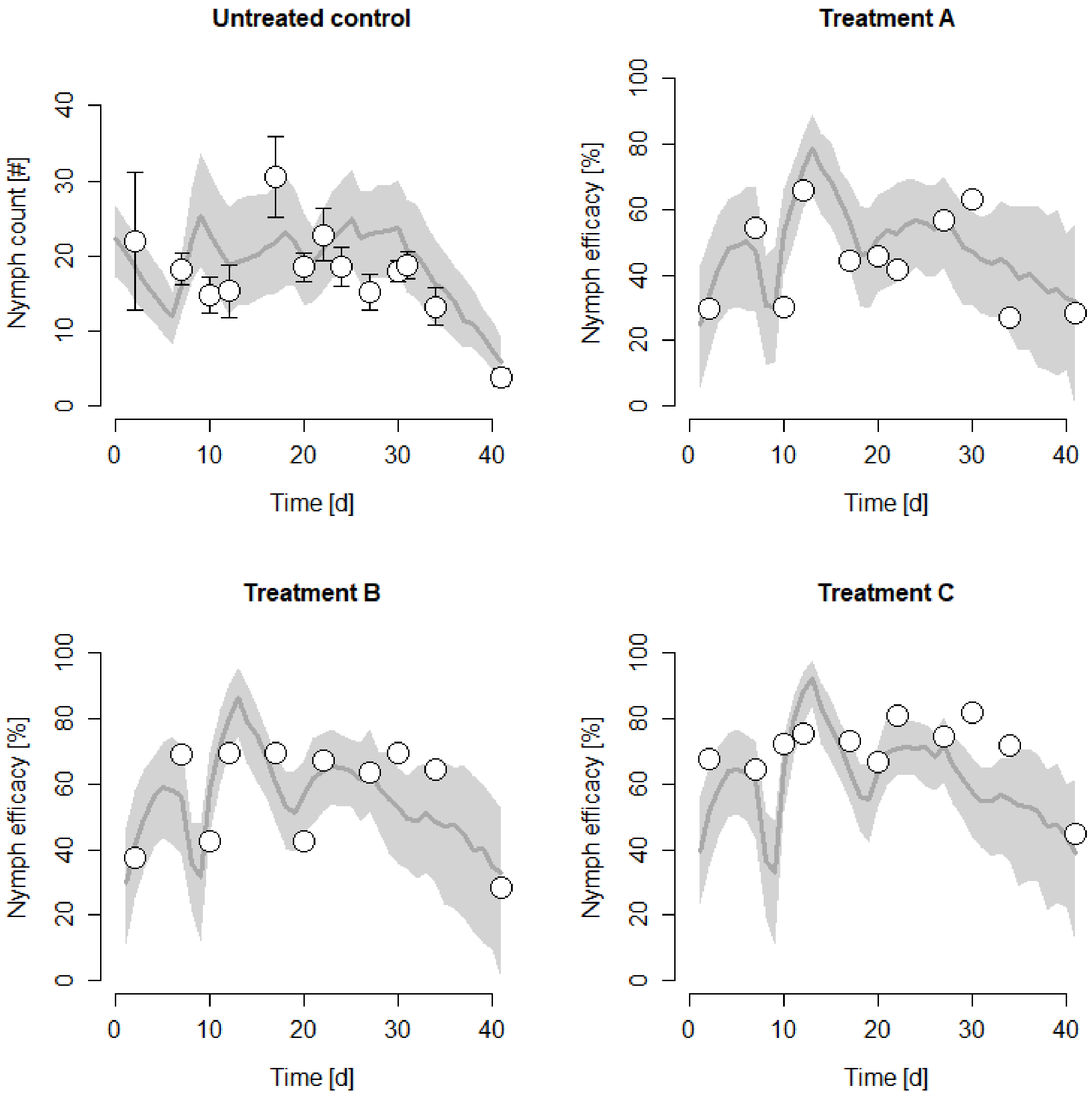

Figure S2.9: Nymph count and efficacy quantified in **Field Trial 2** (for details see Table S2.1). Dots are measured data (means and standard deviation for counts and means for efficacy based on counts) while the lines and grey shades represent the median and 95% prediction intervals of 100 Monte-Carlo simulations. Note, this is the same figure as in the main text, shown here for completeness.

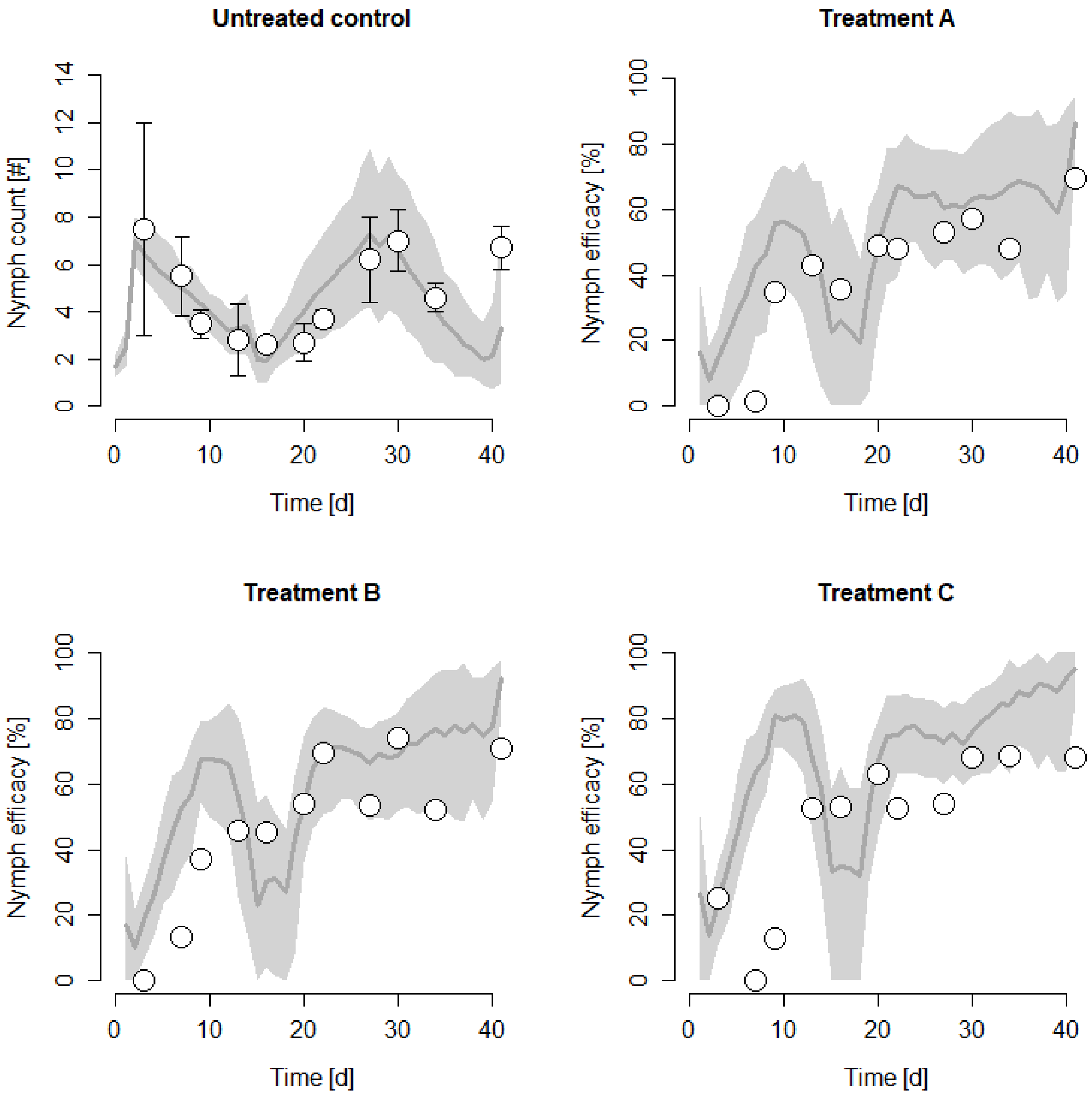

Figure S2.10: Nymph count and efficacy quantified in **Field Trial 3** (for details see Table S2.1). Dots are measured data (means and standard deviation for counts and means for efficacy based on counts) while the lines and grey shades represent the median and 95% prediction intervals of 100 Monte-Carlo simulations.

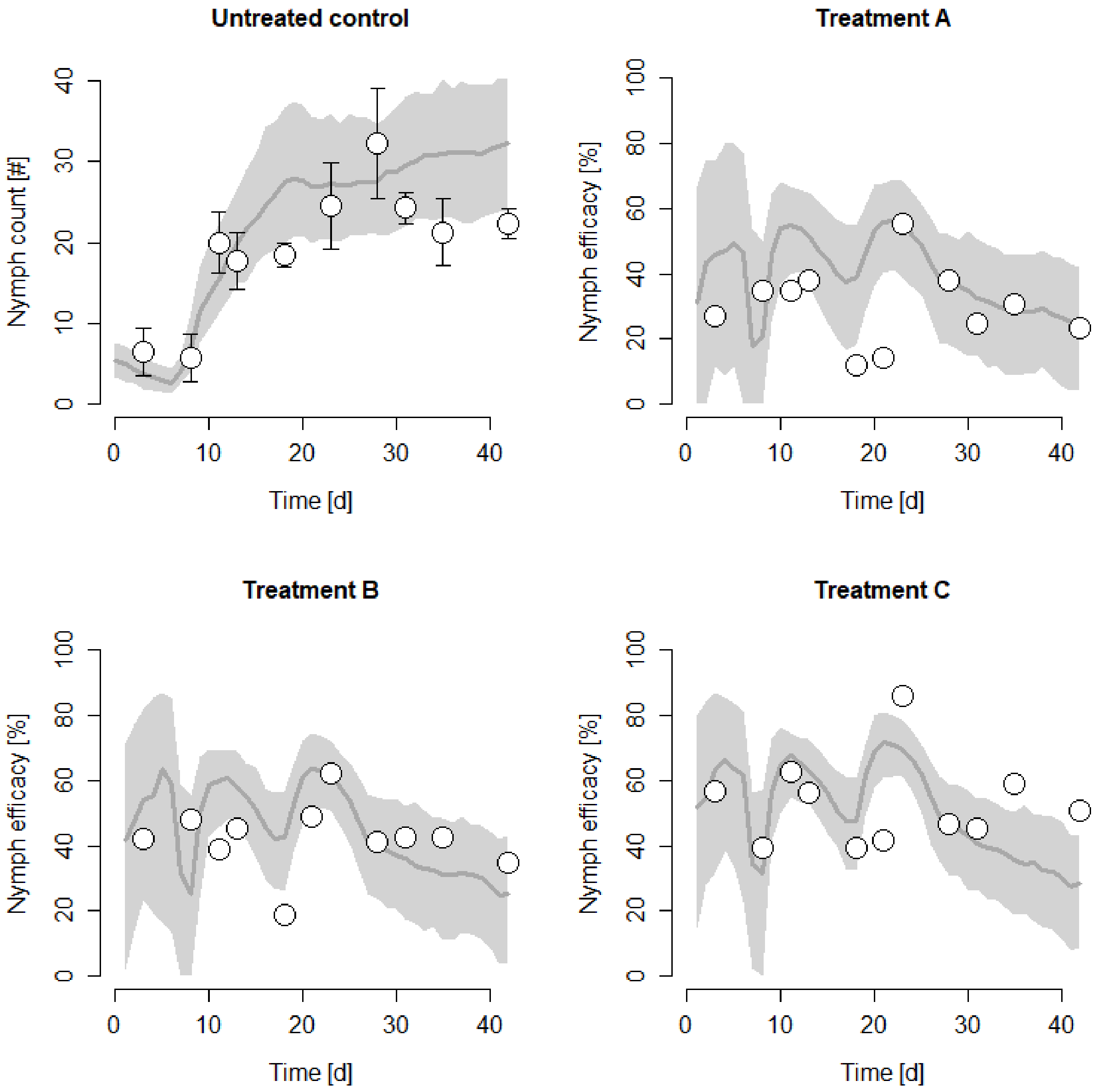

Figure S2.11: Nymph count and efficacy quantified in **Field Trial 4** (for details see Table S2.1). Dots are measured data (means and standard deviation for counts and means for efficacy based on counts) while the lines and grey shades represent the median and 95% prediction intervals of 100 Monte-Carlo simulations.

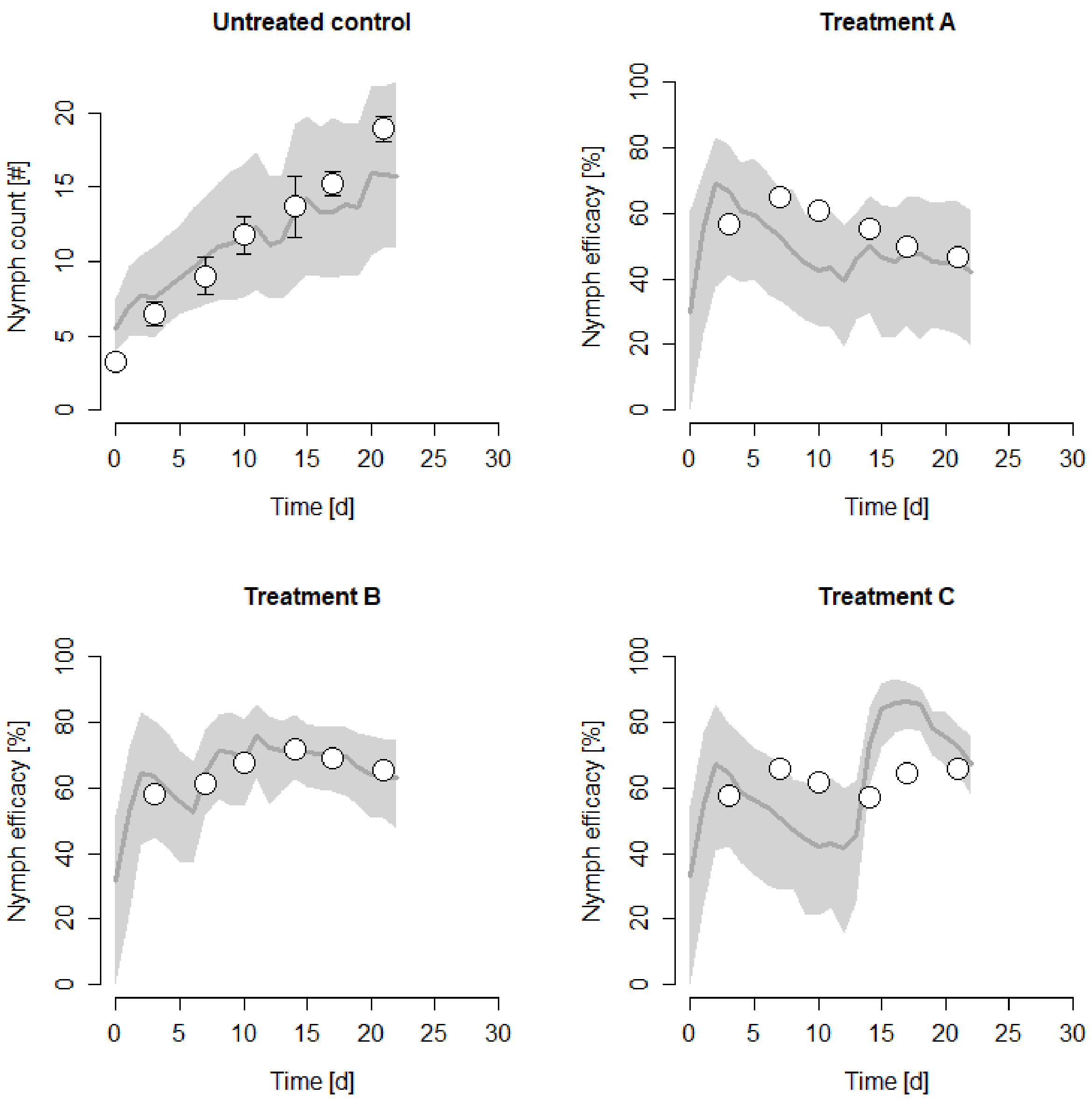

Figure S2.12: Nymph count and efficacy quantified in **Field Trial 5** (for details see Table S2.1). Dots are measured data (means and standard deviation for counts and means for efficacy based on counts) while the lines and grey shades represent the median and 95% prediction intervals of 100 Monte-Carlo simulations.

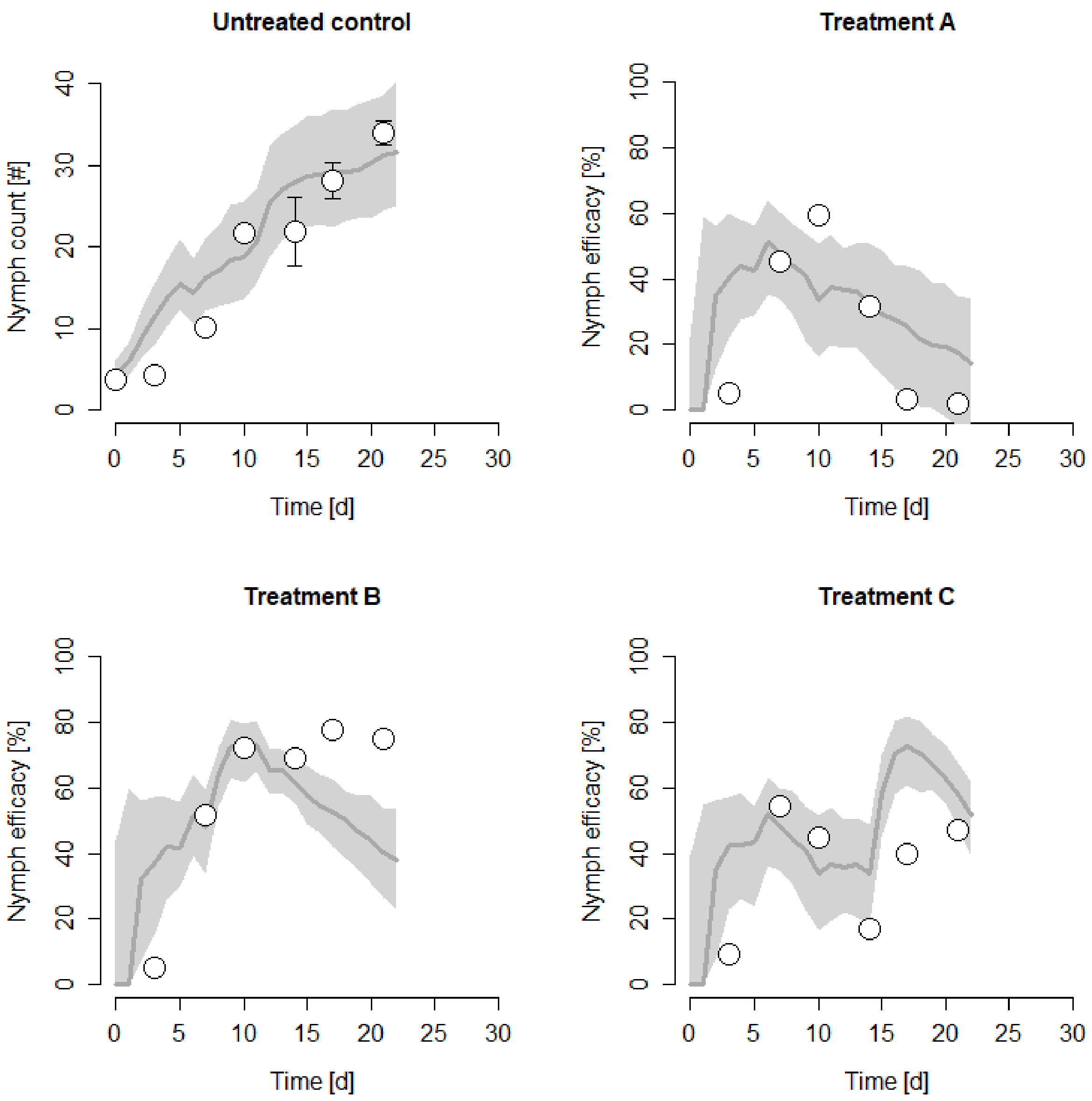

Figure S2.13: Nymph count and efficacy quantified in **Field Trial 6** (for details see Table S2.1). Dots are measured data (means and standard deviation for counts and means for efficacy based on counts) while the lines and grey shades represent the median and 95% prediction intervals of 100 Monte-Carlo simulations.

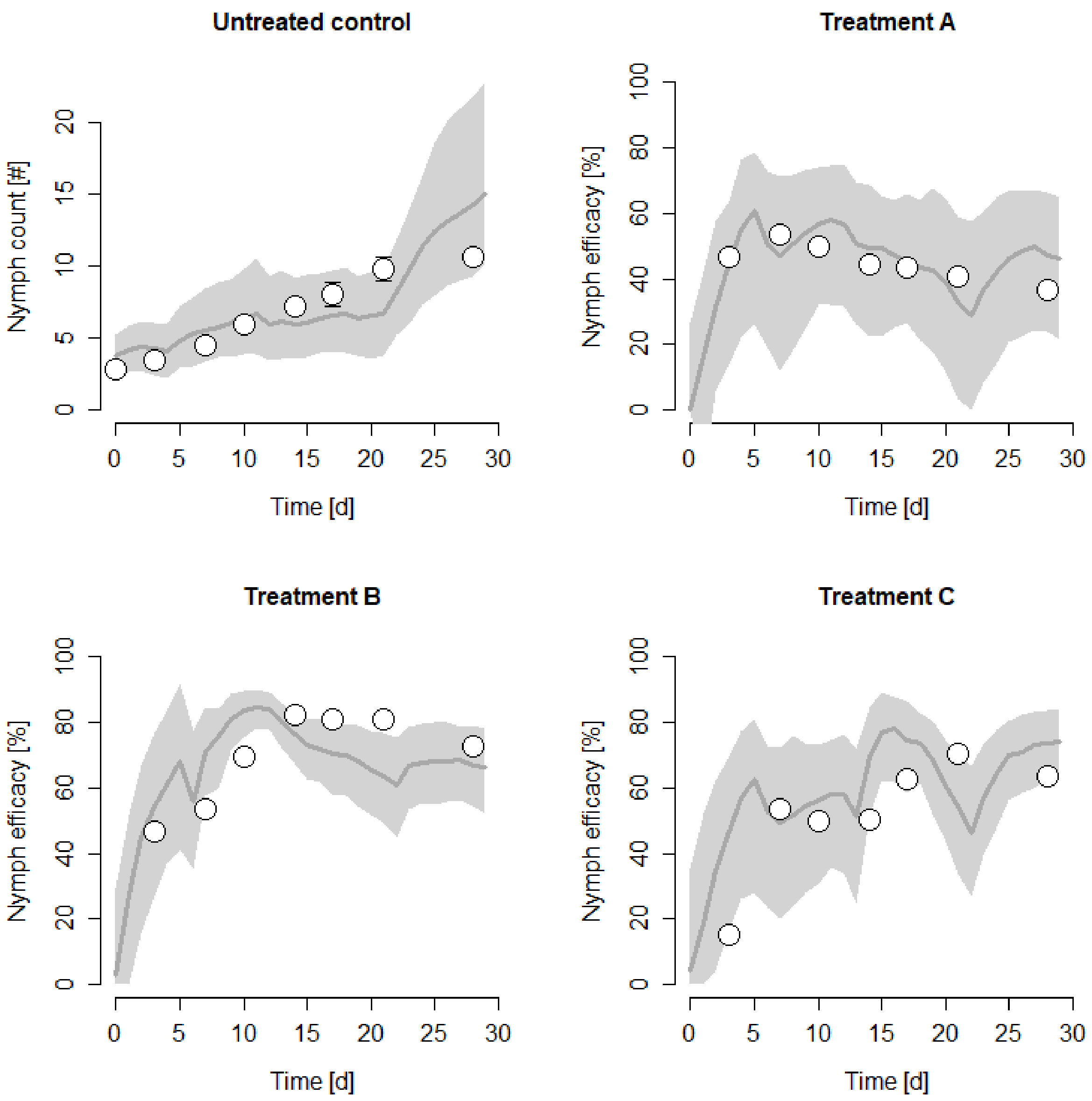

Figure S2.14: Nymph count and efficacy quantified in **Field Trial 7** (for details see Table S2.1). Dots are measured data (means and standard deviation for counts and means for efficacy based on counts) while the lines and grey shades represent the median and 95% prediction intervals of 100 Monte-Carlo simulations.

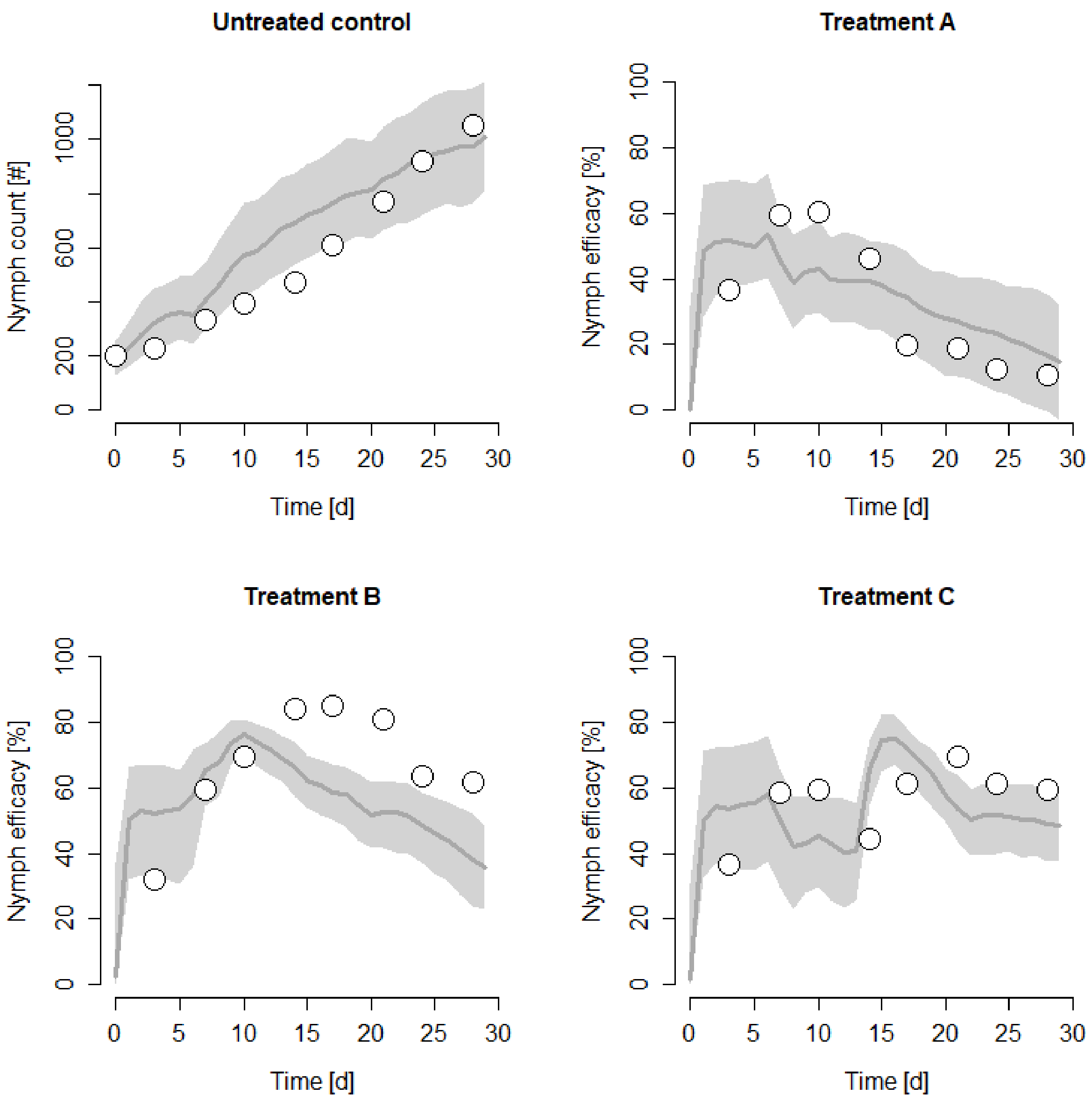

Figure S2.15: Nymph count and efficacy quantified in **Field Trial 8** (for details see Table S2.1). Dots are measured data (means and standard deviation for counts and means for efficacy based on counts) while the lines and grey shades represent the median and 95% prediction intervals of 100 Monte-Carlo simulations.

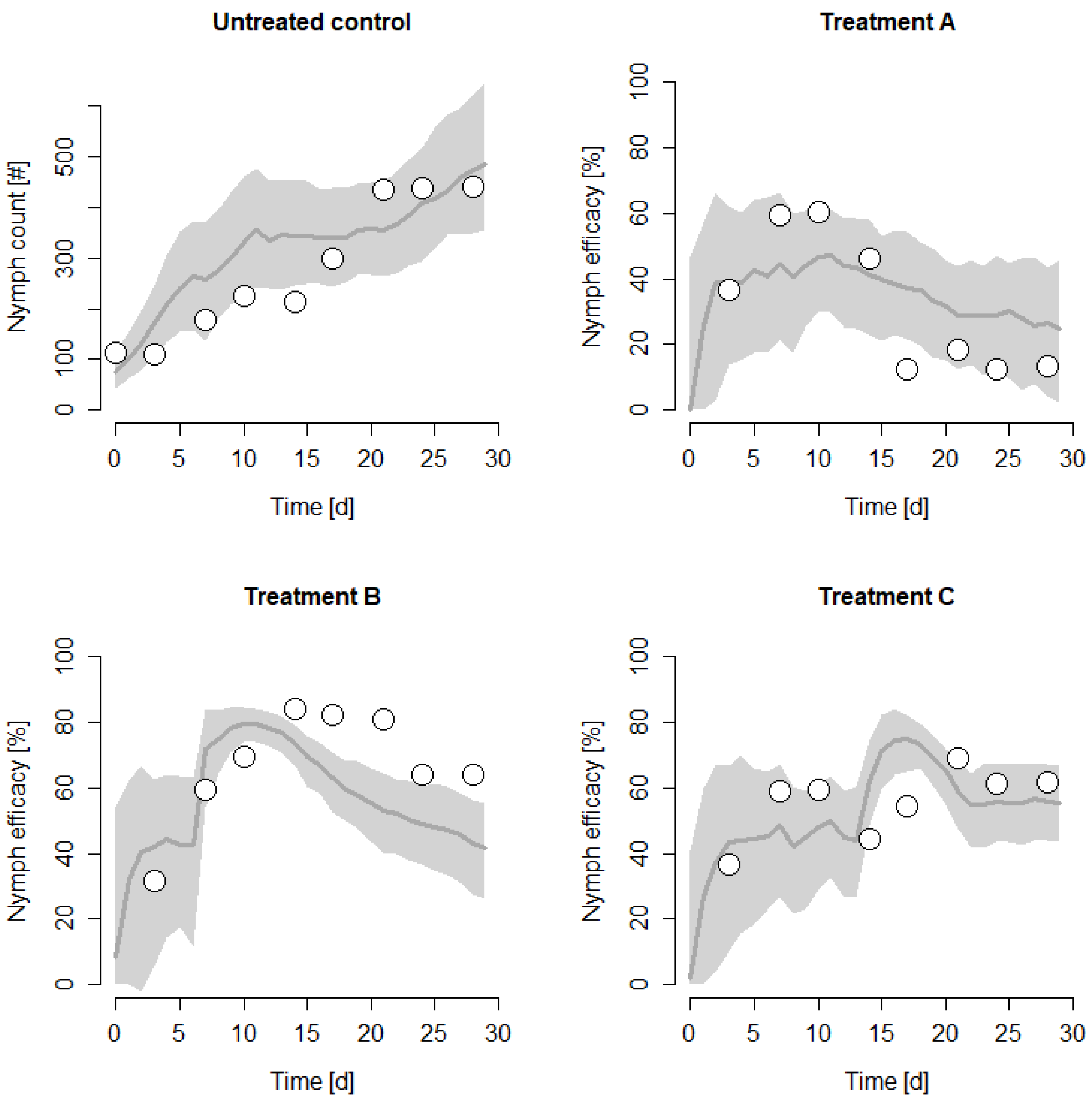

Figure S2.16: Nymph count and efficacy quantified in **Field Trial 9** (for details see Table S2.1). Dots are measured data (means and standard deviation for counts and means for efficacy based on counts) while the lines and grey shades represent the median and 95% prediction intervals of 100 Monte-Carlo simulations.

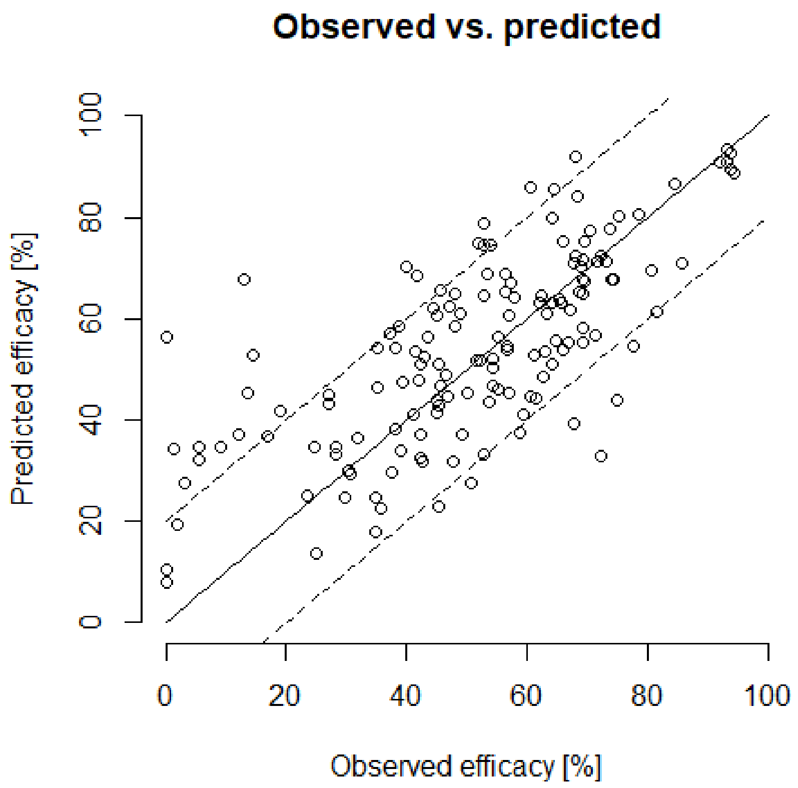

Figure S2.17: Predicted and observed efficacy for nine different field trials.

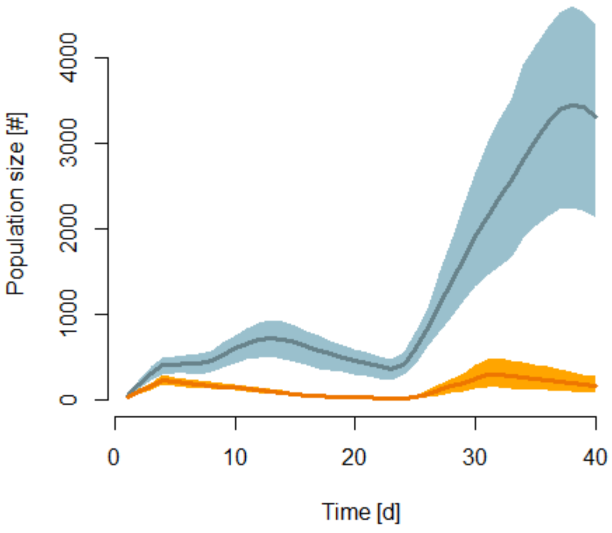

Figure S2.18: Total population size for untreated control (blue) and treated (orange) whitefly populations at an ambient temperature of 30°C. Lines and shades represent the median and 95% prediction intervals of 100 Monte-Carlo simulations. Two applications of 12 g/ha on day 1 and 10 were used to simulate treatment populations. For the initial population we assumed a **synchronized population** of initially 100 2nd instar larvae and two egg laying adults.

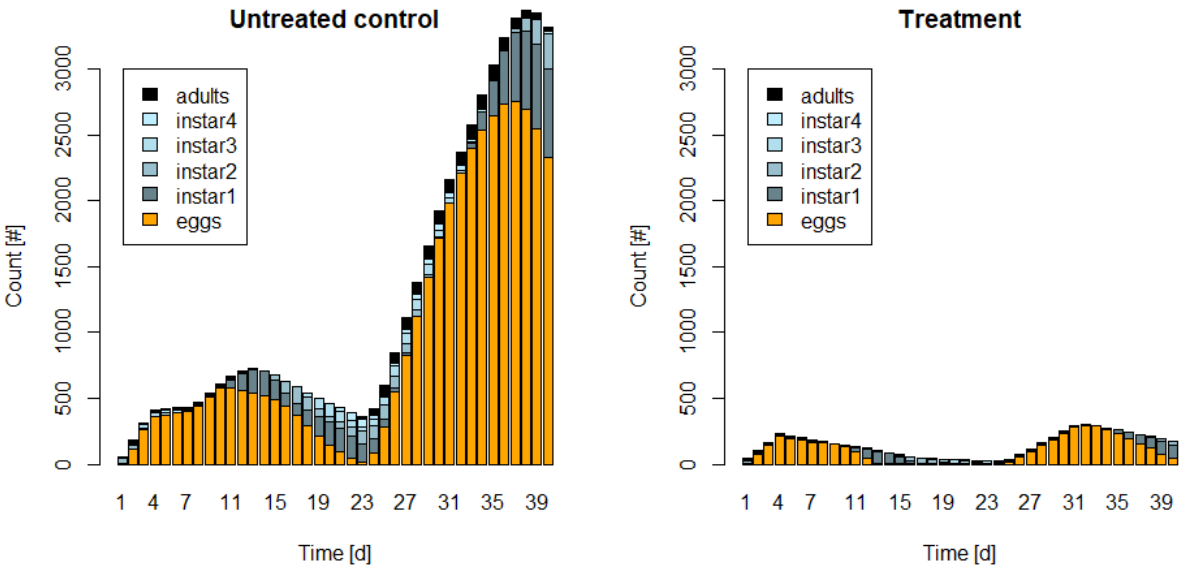

Figure S2.19: Population demography for untreated control and treated whitefly populations at an ambient temperature of 30°C. Different colors refer to immature and mature stages of *B. tabaci*. Model simulations are identical to the ones presented in Figure S2.18.

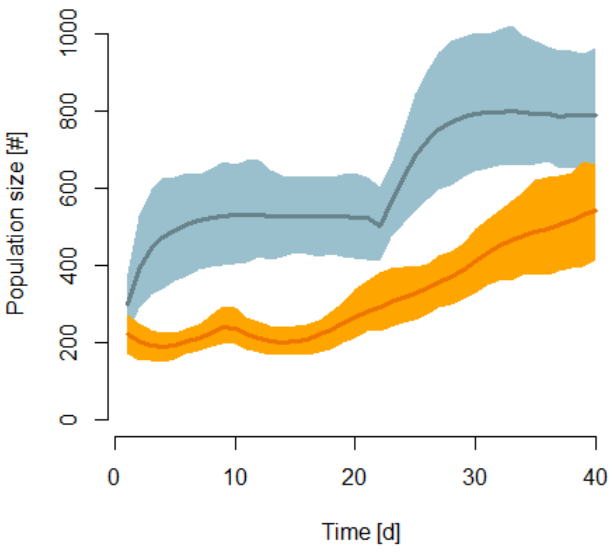

Figure S2.20: Total population size for untreated control (blue) and treated (orange) whitefly populations at an ambient temperature of 30°C. Lines and shades represent the median and 95% prediction intervals of 100 Monte-Carlo simulations. Two applications of 12 g/ha on day 1 and 10 were used to simulate treatment populations. For the initial population we assumed a **more heterogeneous population** of initially 100 nymphs (structural length of 0.0434 ± 0.01 cm) and a continuous **low immigration rate** (at 2 adults per day) and a default emigration rate of 50%. Note that we assume adult immigration from untreated areas and egg laying of these individuals is initially not affected.

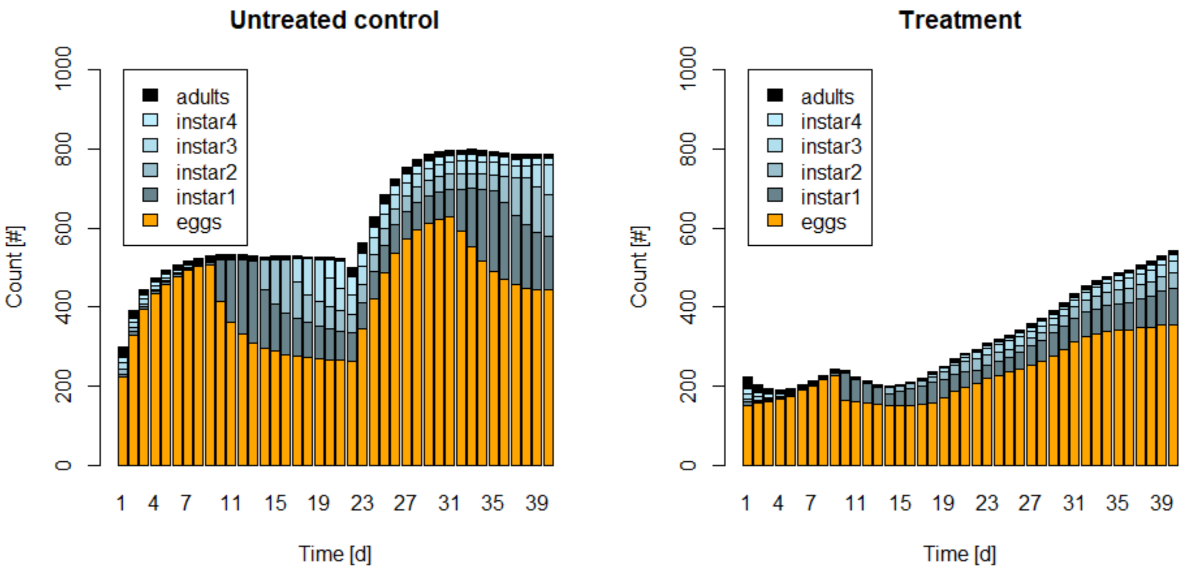

Figure S2.21: Population demography for untreated control and treated whitefly populations at an ambient temperature of 30°C. Different colors refer to immature and mature stages of *B. tabaci*. Model simulations are identical to the ones presented in Figure S2.20.

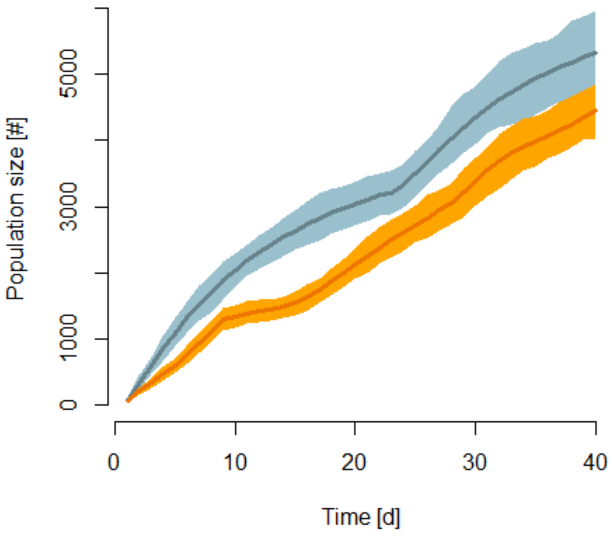

Figure S2.22: Total population size for untreated control (blue) and treated (orange) whitefly populations at an ambient temperature of 30°C. Lines and shades represent the median and 95% prediction intervals of 100 Monte-Carlo simulations. Two applications of 12 g/ha on day 1 and 10 were used to simulate treatment populations. For the initial population we assumed a **heterogeneous population** of initially 50 individuals (uniform distribution from egg to adult) and a continuous **high immigration rate** (at 20 adults per day) and a default emigration rate of 50%. Note that we assume adult immigration from untreated areas and egg laying of these individuals is initially not affected.

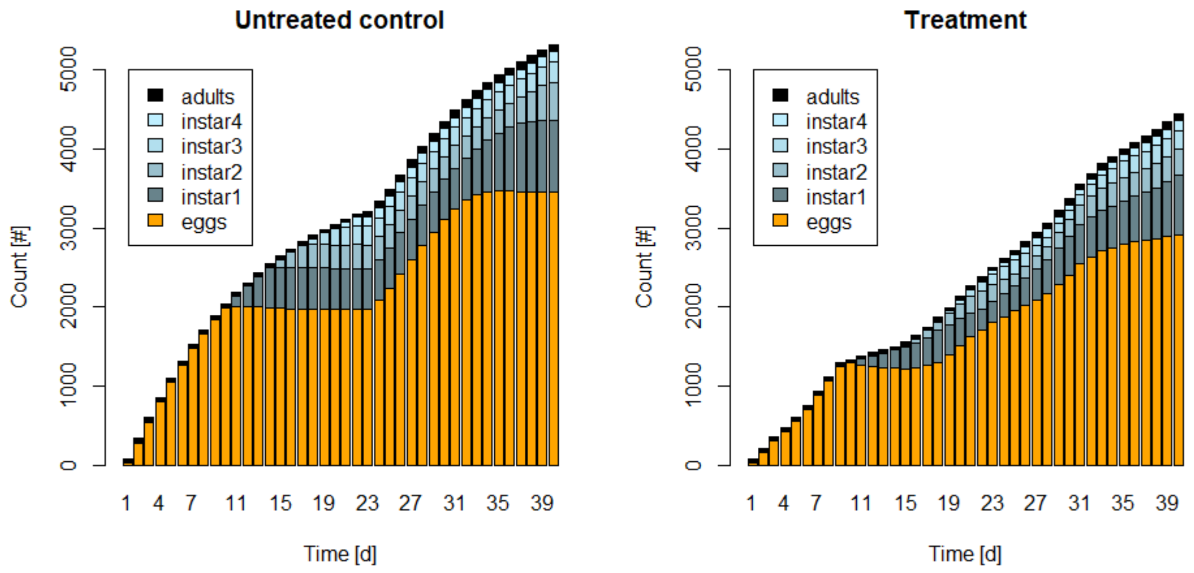

Figure S2.23: Population demography for untreated control and treated whitefly populations at an ambient temperature of 30°C. Different colors refer to immature and mature stages of *B. tabaci*. Model simulations are identical to the ones presented in Figure S2.22.

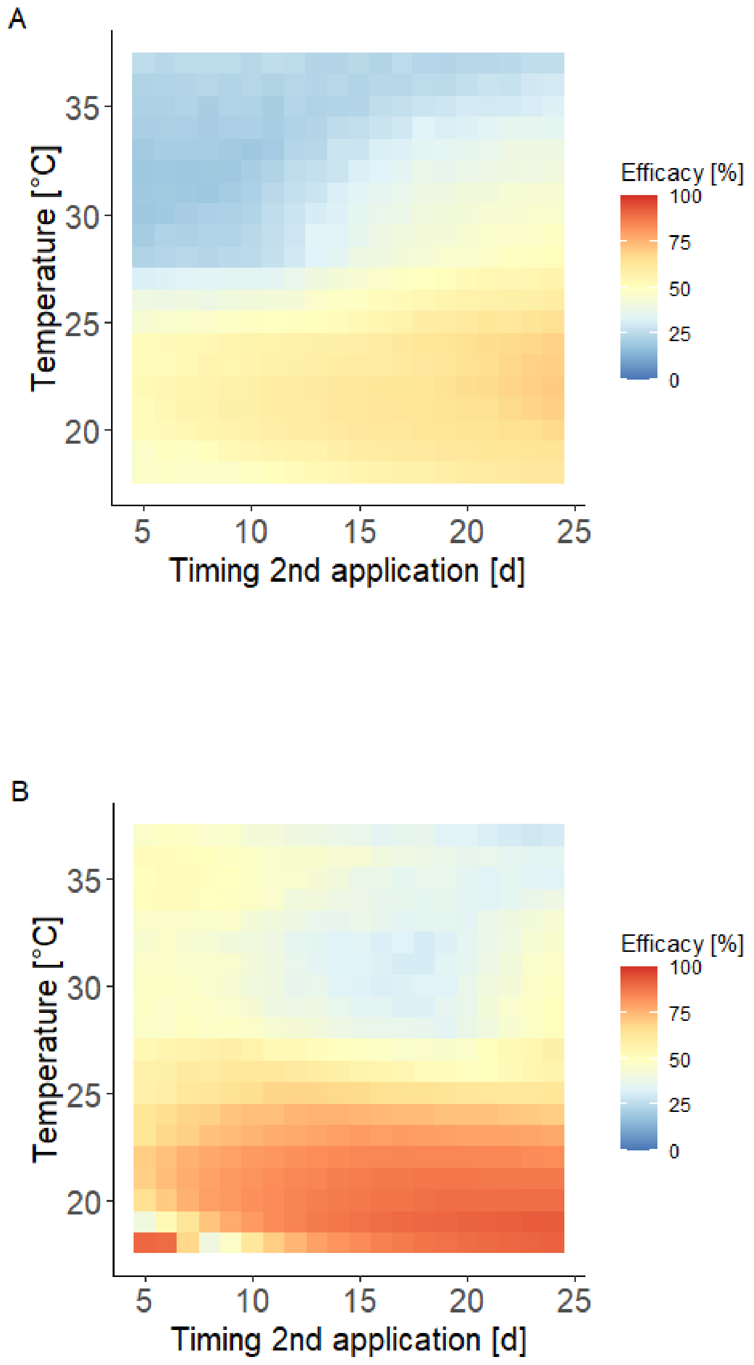

Figure S2.24: Predicted efficacy (shades) as function of ambient temperature and timing of a second treatment for A) the total population (including mature and immature stages) and B) nymphs only. Here, simulated efficacy was evaluated 14 days after the respective second application. Efficacy predictions are based on the median of 100 Monte-Carlo simulations for two applications of 12 g/ha for the initial population we assumed a **heterogeneous population** of initially 50 individuals (uniform distribution from egg to adult) and a continuous **high immigration rate** (at 20 adults per day) and a default emigration rate of 50%. See Figures S2.22-23 for an example simulation.

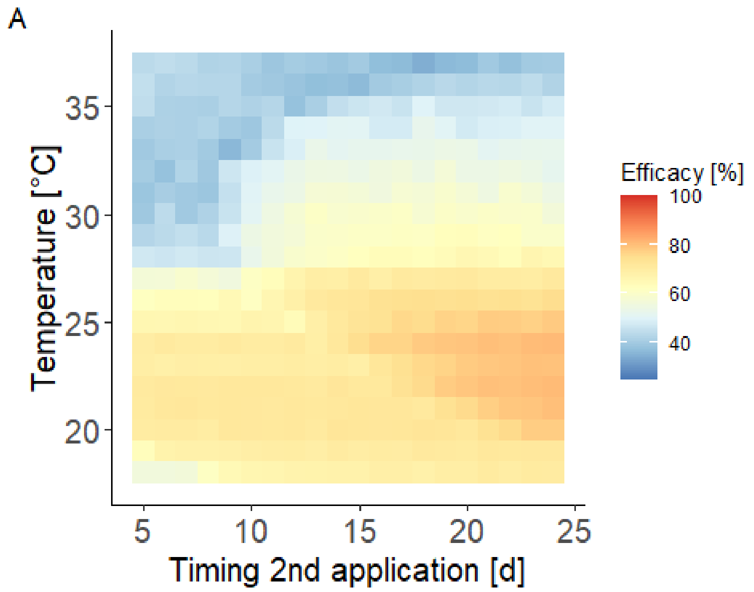

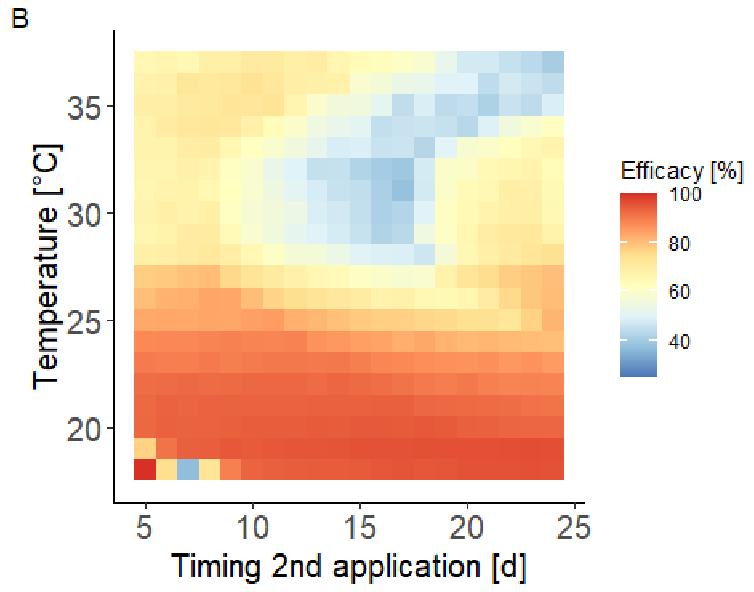

Figure S2.25: Predicted efficacy (shades) as function of ambient temperature and timing of a second treatment for A) the total population (including mature and immature stages) and B) nymphs only. Here, simulated efficacy was evaluated 14 days after the respective second application. Efficacy predictions are based on the median of 100 Monte-Carlo simulations for two applications of **24 g/ha** for the initial population we assumed a **more heterogeneous population** of initially 100 nymphs (structural length of 0.043 ± 0.01 cm) and a continuous **low immigration rate** (at 2 adults per day) and a default emigration rate of 50%. See Figures S2.20-21 for an example simulation.

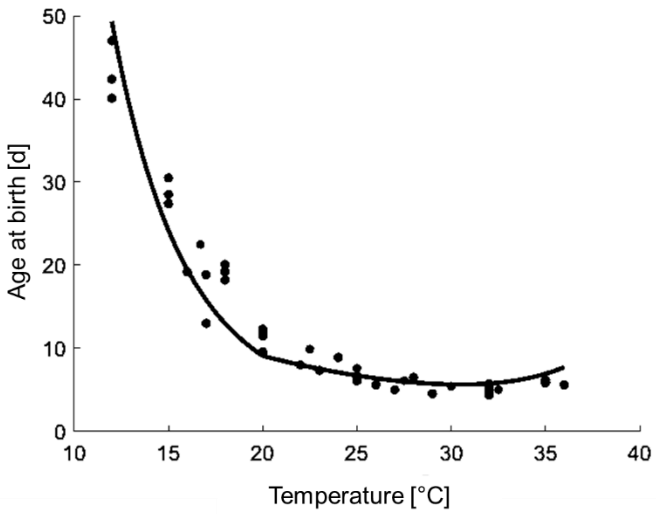

Figure S2.26: Egg development time (age at birth in DEB terms) as function of temperature. Dots and line represent data and model fit respectively. The figure was modified from the add-my-pet entry for *Bemisia tabaci* [3].

References

1. Brand, D.W., Cotton Growth and Developmental Responses to Multiple Environmental Stresses. Theses and Dissertations 1196. **2016**. Available online at: https://scholarsjunction.msstate.edu/td/1196
2. Naranjo, S. E.; Ellsworth, P. C. Mortality Dynamics and Population Regulation in *Bemisia tabaci*. *Entomol. Exp. Appl.* **2005**, *116*, 93–108.
3. Gergs, A. AmP *Bemisia tabaci*, Version 2024/03/19. **2024**. Available online at: https://www.bio.vu.nl/thb/deb/deblab/add_my_pet/entries_web/Bemisia_tabaci/Bemisia_tabaci_res.html
