# Supplementary material for: Optimizing Control Strategies for the Cotton Whitefly Bemisia tabaci: Insights from Individual-Based Modeling
Source: Environ Sci Technol. 2026 Jan 20;60(4):3036–45. doi: 10.1021/acs.est.5c13117 (PMC12874526; doi:10.1021/acs.est.5c13117)
Supplement: Supplementary file 3 [file es5c13117_si_003.zip › SI3_data and code/IBM executable/report_Bemisia_tabaci.html]

Bemisia\_tabaci


|  | | Bemisia tabaci | |  | date: 2024/11/28 |
| --- | --- | --- | --- | --- | --- |
| symbol | units | value | °C | func resp | description |
| model | - | abp | NA | NA | typified model |
| T\_typical | °C | 26 | NA | NA | typical body temperature |
| T\_A | K | 5805 | NA | NA | Arrhenius temperature |
| z | - | 0.3165 | NA | NA | zoom factor |
| F\_m | l/d.cm^2 | 6.5 | 20 | NA | {F\_m}, max spec searching rate |
| kap\_X | - | 0.8 | NA | NA | digestion efficiency of food to reserve |
| kap\_P | - | 0.1 | NA | NA | faecation efficiency of food to faeces |
| v | cm/d | 0.002947 | 20 | NA | energy conductance |
| kap | - | 0.9999 | NA | NA | allocation fraction to soma |
| kap\_R | - | 0.95 | NA | NA | reproduction efficiency |
| p\_M | J/d.cm^3 | 40.53 | 20 | NA | [p\_M], vol-spec somatic maint |
| p\_T | J/d.cm^2 | 0 | 20 | NA | {p\_T}, surf-spec somatic maint |
| k\_J | 1/d | 0.002 | 20 | NA | maturity maint rate coefficient |
| E\_G | J/cm^3 | 4423 | NA | NA | [E\_G], spec cost for structure |
| E\_Hb | J | 1.912e-07 | NA | NA | maturity at birth |
| E\_Hp | J | 6.826e-06 | NA | NA | maturity at puberty |
| h\_a | 1/d^2 | 0.1968 | 20 | NA | Weibull aging acceleration |
| s\_G | - | 0.0001 | NA | NA | Gompertz stress coefficient |
| d\_X | g/cm^3 | 0.17 | NA | NA | specific density of food |
| d\_V | g/cm^3 | 0.17 | NA | NA | specific density of structure |
| d\_E | g/cm^3 | 0.17 | NA | NA | specific density of reserve |
| d\_P | g/cm^3 | 0.17 | NA | NA | specific density of faeces |
| mu\_X | J/ mol | 525000 | NA | NA | chemical potential of food |
| mu\_V | J/ mol | 500000 | NA | NA | chemical potential of structure |
| mu\_E | J/ mol | 550000 | NA | NA | chemical potential of reserve |
| mu\_P | J/ mol | 480000 | NA | NA | chemical potential of faeces |
| mu\_C | J/ mol | 0 | NA | NA | chemical potential of CO2 |
| mu\_H | J/ mol | 0 | NA | NA | chemical potential of H2O |
| mu\_O | J/ mol | 0 | NA | NA | chemical potential of O2 |
| mu\_N | J/ mol | 48800 | NA | NA | chemical potential of N-waste |
| n\_CX | - | 1 | NA | NA | chem. index of carbon in food |
| n\_HX | - | 1.8 | NA | NA | chem. index of hydrogen in food |
| n\_OX | - | 0.5 | NA | NA | chem. index of oxygen in food |
| n\_NX | - | 0.15 | NA | NA | chem. index of nitrogen in food |
| n\_CV | - | 1 | NA | NA | chem. index of carbon in structure |
| n\_HV | - | 1.8 | NA | NA | chem. index of hydrogen in structure |
| n\_OV | - | 0.5 | NA | NA | chem. index of oxygen in structure |
| n\_NV | - | 0.15 | NA | NA | chem. index of nitrogen in structure |
| n\_CE | - | 1 | NA | NA | chem. index of carbon in reserve |
| n\_HE | - | 1.8 | NA | NA | chem. index of hydrogen in reserve |
| n\_OE | - | 0.5 | NA | NA | chem. index of oxygen in reserve |
| n\_NE | - | 0.15 | NA | NA | chem. index of nitrogen in reserve |
| n\_CP | - | 1 | NA | NA | chem. index of carbon in faeces |
| n\_HP | - | 1.8 | NA | NA | chem. index of hydrogen in faeces |
| n\_OP | - | 0.5 | NA | NA | chem. index of oxygen in faeces |
| n\_NP | - | 0.15 | NA | NA | chem. index of nitrogen in faeces |
| n\_CC | - | 1 | NA | NA | chem. index of carbon in CO2 |
| n\_HC | - | 0 | NA | NA | chem. index of hydrogen in CO2 |
| n\_OC | - | 2 | NA | NA | chem. index of oxygen in CO2 |
| n\_NC | - | 0 | NA | NA | chem. index of nitrogen in CO2 |
| n\_CH | - | 0 | NA | NA | chem. index of carbon in H2O |
| n\_HH | - | 2 | NA | NA | chem. index of hydrogen in H2O |
| n\_OH | - | 1 | NA | NA | chem. index of oxygen in H2O |
| n\_NH | - | 0 | NA | NA | chem. index of nitrogen in H2O |
| n\_CO | - | 0 | NA | NA | chem. index of carbon in O2 |
| n\_HO | - | 0 | NA | NA | chem. index of hydrogen in O2 |
| n\_OO | - | 2 | NA | NA | chem. index of oxygen in O2 |
| n\_NO | - | 0 | NA | NA | chem. index of nitrogen in O2 |
| n\_CN | - | 1 | NA | NA | chem. index of carbon in N-waste |
| n\_HN | - | 0.8 | NA | NA | chem. index of hydrogen in N-waste |
| n\_ON | - | 0.6 | NA | NA | chem. index of oxygen in N-waste |
| n\_NN | - | 0.8 | NA | NA | chem. index of nitrogen in N-waste |
| c\_T | - | 1.48761 | 26 | NA | Temperature Correction factor |
| p\_Am | J/d.cm^2 | 12.829 | 20 | NA | {p\_Am}, spec assimilation flux |
| w\_X | g/mol | 23.9 | NA | NA | molecular weight for water-free food |
| w\_V | g/mol | 23.9 | NA | NA | molecular weight for water-free structure |
| w\_E | g/mol | 23.9 | NA | NA | molecular weight for water-free reserve |
| w\_P | g/mol | 23.9 | NA | NA | molecular weight for water-free product (feaces) |
| M\_V | mol/cm^3 | 0.00711297 | NA | NA | [M\_V], volume-specific mass of structure |
| y\_V\_E | mol/mol | 0.884498 | NA | NA | yield of structure on reserve |
| y\_E\_V | mol/mol | 1.13059 | NA | NA | yield of reserve on structure |
| k\_M | 1/d | 0.00916346 | 20 | NA | somatic maintenance rate coefficient |
| k | - | 0.218258 | NA | NA | maintenance ratio |
| E\_m | J/cm^3 | 4353.25 | NA | NA | [E\_m], reserve capacity |
| m\_Em | mol/mol | 1.11276 | NA | NA | reserve capacity |
| g | - | 1.01612 | NA | NA | energy investment ratio |
| L\_m | cm | 0.3165 | NA | NA | maximum structural length |
| L\_T | cm | 0 | NA | NA | heating length (also applies to osmotic work) |
| l\_T | - | 0 | NA | NA | scaled heating length |
| ome | - | 1.11276 | NA | NA | \omega, contribution of ash free dry mass of reserve to total ash free dry biomass |
| J\_E\_Am | mol/d.cm^2 | 2.33255e-05 | 20 | NA | {J\_EAm}, max surface-spec assimilation flux |
| y\_E\_X | mol/mol | 0.763636 | NA | NA | yield of reserve on food |
| y\_X\_E | mol/mol | 1.30952 | NA | NA | yield of food on reserve |
| p\_Xm | J/d.cm^2 | 16.0363 | 20 | NA | {p\_Xm}, max spec feeding power |
| J\_X\_Am | mol/d.cm^2 | 3.05453e-05 | 20 | NA | {J\_XAm}, max surface-spec feeding flux |
| y\_P\_X | mol/mol | 0.109375 | NA | NA | yield of faeces on food |
| y\_X\_P | mol/mol | 9.14286 | NA | NA | yield of food on faeces |
| y\_P\_E | mol/mol | 0.143229 | NA | NA | yield of faeces on reserve |
| eta\_XA | mol/J | 2.38095e-06 | NA | NA | mass-power couplers for food on assimilation |
| eta\_PA | mol/J | 2.60417e-07 | NA | NA | mass-power couplers for product on assimlation |
| eta\_VG | mol/J | 1.60818e-06 | NA | NA | mass-power couplers for structure on growth |
| J\_E\_M | mol/d.cm^3 | 7.36909e-05 | 20 | NA | [J\_EM], vol-spec somatic maint costs |
| J\_E\_T | mol/d.cm^2 | 0 | 20 | NA | {J\_ET}, surface-spec somatic maint costs |
| j\_E\_M | mol/d.mol | 0.0103601 | 20 | NA | mass-spec somatic maint costs |
| j\_E\_J | mol/d.mol | 0.00226117 | 20 | NA | mass-spec maturity maint costs |
| kap\_G | - | 0.804089 | NA | NA | \kappa\_G, growth efficiency |
| E\_V | J/cm^3 | 3556.49 | NA | NA | [E\_V], volume-specific energy of structure |
| K | mol X/l | 4.69928e-06 | NA | NA | half-saturation coefficient |
| E\_Hb | J | 1.912e-07 | NA | NA | maturity level at birth |
| M\_Hb | mol | 3.47636e-13 | NA | NA | maturity level at birth |
| U\_Hb | cm^2.d | 1.49037e-08 | 20 | NA | scaled maturity level at birth |
| V\_Hb | cm^2.d | 0.000149037 | 20 | NA | scaled maturity level at birth |
| u\_Hb | - | 1.36335e-09 | NA | NA | scaled maturity level at birth |
| v\_Hb | - | 1.36335e-05 | NA | NA | scaled maturity level at birth |
| E\_Hp | J | 6.826e-06 | NA | NA | maturity level at puberty |
| M\_Hp | mol | 1.24109e-11 | NA | NA | maturity level at puberty |
| U\_Hp | cm^2.d | 5.32075e-07 | 20 | NA | scaled maturity level at puberty |
| V\_Hp | cm^2.d | 0.00532075 | 20 | NA | scaled maturity level at puberty |
| u\_Hp | - | 4.86726e-08 | NA | NA | scaled maturity level at puberty |
| v\_Hp | - | 0.000486726 | NA | NA | scaled maturity level at puberty |
| t\_E | d | 72.1947 | 26 | NA | maximum reserve residence time |
| t\_starve | d | 72.202 | 26 | 0 | maximum survival time when starved |
| E\_Hj | J | 6.816e-06 | NA | NA | maturity level at metamorphosis |
| M\_Hj | mol | -9.98759e-09 | NA | NA | maturity level at metamorphosis |
| U\_Hj | cm^2.d | 5.22075e-07 | 20 | NA | scaled maturity level at metamorphosis |
| V\_Hj | cm^2.d | 0.00532074 | 20 | NA | scaled maturity level at metamorphosis |
| u\_Hj | - | 3.86726e-08 | NA | NA | scaled maturity level at metamorphosis |
| v\_Hj | - | 0.000486716 | NA | NA | scaled maturity level at metamorphosis |
| s\_M | - | 3.27488 | NA | 1 | acceleration factor at f=1 |
| r\_j | 1/d | 0.282629 | 26 | 1 | exponential growth rate |
| r\_B | 1/d | 0.00229011 | 26 | 1 | von Bertalanffy growth rate |
| W\_dWm | g | 3.14445e-05 | NA | 1 | wet weight at maximum growth |
| dWm | g/d | 1.32205e-05 | 26 | 1 | maximum growth in wet weight |
| U\_E0 | cm^2.d | 0.000293661 | 20 | 1 | scaled initial reserve |
| E\_0 | J | 0.00376738 | NA | 1 | initial reserve |
| M\_E0 | mol | 6.84978e-09 | NA | 1 | initial reserve |
| Wd\_0 | g | 1.6371e-07 | NA | 1 | initial dry weight |
| Ww\_0 | g | 9.62999e-07 | NA | 1 | initial wet weight |
| l\_b | - | 0.0237317 | NA | 1 | scaled structural length at birth |
| L\_b | cm | 0.00751109 | NA | 1 | structural length at birth |
| M\_Vb | mol | 3.01412e-09 | NA | 1 | structural mass at birth |
| del\_Ub | - | 0.489647 | NA | 1 | fraction of reserve left at birth |
| Ww\_b | g | 8.95279e-07 | NA | 1 | wet weight at birth |
| Wd\_b | g | 1.52197e-07 | NA | 1 | dry weight at birth |
| E\_Wb | J | 0.00335174 | NA | 1 | energy content at birth |
| a\_b | d | 6.10556 | 26 | 1 | age at birth |
| g\_Hb | - | 1.03649 | NA | NA | energy divestment ratio at birth |
| l\_j | - | 0.077718 | NA | 1 | scaled structural length at metamorphosis |
| L\_j | cm | 0.0245977 | NA | 1 | structural length at metamorphosis |
| M\_Vj | mol | 1.05861e-07 | NA | 1 | structural mass at metamorphosis |
| M\_Ej | mol | 1.05861e-07 | NA | 1 | reserve mass at metamorphosis |
| Ww\_j | g | 3.14438e-05 | NA | 1 | wet weight at metamorphosis |
| Wd\_j | g | 5.34545e-06 | NA | 1 | dry weight at metamorphosis |
| E\_Wj | J | 0.117719 | NA | 1 | energy content at metamorphosis |
| a\_j | d | 18.6974 | 26 | 1 | age at metamorphosis |
| s\_Hbp | - | 0.0280105 | NA | NA | maturity ratio |
| s\_HLbp | - | 0.983802 | NA | 1 | maturity density ratio at f=1 |
| l\_p | - | 0.0777185 | NA | 1 | scaled structural length at puberty |
| L\_p | cm | 0.0245979 | NA | 1 | structural length at puberty |
| M\_Vp | mol | 1.05863e-07 | NA | 1 | structural mass at puberty |
| M\_Ep | mol | 1.178e-07 | NA | 1 | reserve mass at puberty |
| Ww\_p | g | 3.14445e-05 | NA | 1 | wet weight at puberty |
| Wd\_p | g | 5.34556e-06 | NA | 1 | dry weight at puberty |
| E\_Wp | J | 0.117722 | NA | 1 | energy content at puberty |
| a\_p | d | 18.6975 | 26 | 1 | age at puberty |
| g\_Hp | - | 1.05356 | NA | NA | energy divestment ratio at puberty |
| s\_s | - | 3.024e-10 | NA | 1 | supply stress |
| l\_i | - | 0.0777195 | NA | 1 | ultimate scaled structural length |
| L\_i | cm | 0.0245982 | NA | 1 | ultimate structural length |
| M\_Vi | mol | 1.05868e-07 | NA | 1 | ultimate structural mass |
| M\_Ei | mol | 1.17805e-07 | NA | 1 | ultimate reserve mass |
| Ww\_i | g | 3.14457e-05 | NA | 1 | ultimate wet weight |
| Wd\_i | g | 5.34577e-06 | NA | 1 | ultimate dry weight |
| E\_Wi | J | 0.117726 | NA | 1 | ultimate energy content |
| xi\_WE | kJ/ g | 22.0224 | NA | 1 | whole-body energy density of dry biomass (no reprod buffer) |
| del\_Wb | - | 0.0284706 | NA | 1 | birth weight as fraction of maximum weight |
| del\_Wp | - | 0.999961 | NA | 1 | puberty weight as fraction of maximum weight |
| del\_V | - | 0.473315 | NA | 1 | fraction of max weight that is structure |
| h\_W | 1/d | 0.348607 | 26 | 1 | Weibull ageing rate |
| h\_G | 1/d | 2.74e-08 | 26 | 1 | Gompertz aging rate |
| S\_b | - | 0.101003 | NA | 1 | survival probability at birth |
| S\_p | - | 1e-06 | NA | 1 | survival probability at puberty |
| a\_m | d | 6.20083 | 26 | 1 | life span |
| a\_99 | d | -2887.58 | 26 | 1 | age at length 0.99 \* L\_i |
| R\_i | 1/d | 9.30953 | 26 | 1 | ultimate reproduction rate |
| N\_i | # | -116.338 | NA | 1 | life time reproductive output |
| M\_E0\_min\_G | mol | 3.61315e-09 | NA | NA | egg mass whereby growth ceases at birth |
| M\_E0\_min\_R | mol | 3.57791e-09 | NA | NA | egg mass whereby maturation ceases at birth |
| eb\_min\_G | - | 0.0233924 | NA | NA | scaled reserve density whereby growth ceases at birth |
| eb\_min\_R | - | 0.00543531 | NA | NA | scaled reserve density whereby maturation ceases at birth |
| ep\_min | - | 0.0233841 | NA | NA | scaled reserve density whereby maturation and growth cease at puberty |
| sM\_min | - | 2.02484 | NA | NA | acceleration factor whereby maturation ceases at puberty |
| p\_Xb | J/d | 0.00134585 | 26 | 1 | food intake at birth |
| J\_Xb | mol/d | 2.56353e-09 | 26 | 1 | food intake at birth |
| F\_mb | l/d | 0.000545516 | 26 | 1 | max searching rate at birth |
| p\_Xp | J/d | 0.0144341 | 26 | 1 | food intake at puberty |
| J\_Xp | mol/d | 2.74934e-08 | 26 | 1 | food intake at puberty |
| F\_mp | l/d | 0.00585057 | 26 | 1 | max searching rate at puberty |
| p\_Xi | J/d | 0.0144344 | 26 | 1 | ultimate food intake |
| J\_Xi | mol/d | 2.74941e-08 | 26 | 1 | ultimate food intake |
| F\_mi | l/d | 0.00585072 | 26 | 1 | max ultimate searching rate |
| p\_Ab | J/d | 0.00107711 | 26 | 1 | assimilation at birth |
| p\_Cb | J/d | 0.000555542 | 26 | 1 | mobilisation at birth |
| p\_Sb | J/d | 2.55592e-05 | 26 | 1 | somatic maintenance at birth |
| p\_Jb | J/d | 5.69099e-10 | 26 | 1 | maturity maintenance at birth |
| p\_Gb | J/d | 0.000529927 | 26 | 1 | growth at birth |
| p\_Rb | J/d | 5.49851e-08 | 26 | 1 | maturation at birth |
| p\_Db | J/d | 2.56147e-05 | 26 | 1 | dissipation at birth |
| p\_Ap | J/d | 0.0378156 | 26 | 1 | assimilation at puberty |
| p\_Cp | J/d | 0.0195041 | 26 | 1 | mobilisation at puberty |
| p\_Sp | J/d | 0.000897345 | 26 | 1 | somatic maintenance at puberty |
| p\_Jp | J/d | 2.03082e-08 | 26 | 1 | maturity maintenance at puberty |
| p\_Gp | J/d | 0.0186048 | 26 | 1 | growth at puberty |
| p\_Rp | J/d | 1.93011e-06 | 26 | 1 | reproduction at puberty |
| p\_Dp | J/d | 0.000899296 | 26 | 1 | dissipation at puberty |
| p\_Ai | J/d | 0.0378166 | 26 | 1 | ultimate assimilation |
| p\_Ci | J/d | 0.0195046 | 26 | 1 | ultimate mobilisation |
| p\_Si | J/d | 0.00089738 | 26 | 1 | ultimate somatic maintenance |
| p\_Ji | J/d | 2.0309e-08 | 26 | 1 | ultimate maturity maintenance |
| p\_Gi | J/d | 0 | 26 | 1 | ultimate growth |
| p\_Ri | J/d | 0.0186072 | 26 | 1 | ultimate reproduction |
| p\_Di | J/d | 0.000897497 | 26 | 1 | ultimate dissipation |
| J\_Cb | mol/d | 3.92867e-10 | 26 | 1 | CO2 flux at birth |
| J\_Cp | mol/d | 1.37929e-08 | 26 | 1 | CO2 flux at puberty |
| J\_Ci | mol/d | 1.0616e-08 | 26 | 1 | ultimate CO2 flux |
| J\_Hb | mol/d | 4.21207e-10 | 26 | 1 | water flux at birth |
| J\_Hp | mol/d | 1.47879e-08 | 26 | 1 | water flux at puberty |
| J\_Hi | mol/d | 1.13818e-08 | 26 | 1 | ultimate water flux |
| J\_Ob | mol/d | 4.98639e-10 | 26 | 1 | O2 flux at birth |
| J\_Op | mol/d | 1.75064e-08 | 26 | 1 | O2 flux at puberty |
| J\_Oi | mol/d | 1.34741e-08 | 26 | 1 | ultimate O2 flux |
| J\_Nb | mol/d | 9.06616e-11 | 26 | 1 | N-waste flux at birth |
| J\_Np | mol/d | 3.18297e-09 | 26 | 1 | N-waste flux at puberty |
| J\_Ni | mol/d | 2.44984e-09 | 26 | 1 | ultimate N-waste flux |
| RQ\_b | mol C/mol O | 0.787879 | NA | 1 | resp quotient at birth |
| UQ\_b | mol N/mol O | 0.181818 | NA | 1 | urine quotient at birth |
| WQ\_b | mol H/mol O | 0.8 | NA | 1 | water quotient at birth |
| SDA\_b | mol O/mol X | 0.130957 | NA | 1 | specific dynamic action at birth |
| VO\_b | L/h.g | -0.000327387 | 26 | 1 | dioxygen use per gram max dry weight, <J\_OD> at birth |
| p\_Tb | J/d | 0.000264072 | 26 | 1 | total heat at birth |
| RQ\_p | mol C/mol O | 0.787879 | NA | 1 | resp quotient at puberty |
| UQ\_p | mol N/mol O | 0.181818 | NA | 1 | urine quotient at puberty |
| WQ\_p | mol H/mol O | 0.8 | NA | 1 | water quotient at puberty |
| SDA\_p | mol O/mol X | 0.130957 | NA | 1 | specific dynamic action at puberty |
| VO\_p | L/h.g | -0.000327256 | 26 | 1 | dioxygen use per gram max dry weight, <J\_OD> at puberty |
| p\_Tp | J/d | 0.00927114 | 26 | 1 | total heat at puberty |
| RQ\_i | mol C/mol O | 0.787879 | NA | 1 | ultimate resp quotient |
| UQ\_i | mol N/mol O | 0.181818 | NA | 1 | ultimate urine quotient |
| WQ\_i | mol H/mol O | 0.8 | NA | 1 | ultimate water quotient |
| SDA\_i | mol O/mol X | 0.130957 | NA | 1 | ultimate specific dynamic action |
| VO\_i | L/h.g | -0.000326589 | 26 | 1 | ultimate dioxygen use per gram max dry weight, <J\_OD> |
| p\_Ti | J/d | 0.00562457 | 26 | 1 | ultimate total heat |
