# Supplementary material for: Optimizing Control Strategies for the Cotton Whitefly Bemisia tabaci: Insights from Individual-Based Modeling
Source: Environ Sci Technol. 2026 Jan 20;60(4):3036–45. doi: 10.1021/acs.est.5c13117 (PMC12874526; doi:10.1021/acs.est.5c13117)
Supplement: Supplementary file 3 [file es5c13117_si_003.zip › SI3_data and code/IBM executable/run_DEBibm.nb.html]

Run IBM exe


Code 

- Show All Code
- Hide All Code
- Download Rmd

# Run IBM exe

Supplementary Information to “Optimizing control strategies for the
cotton whitefly Bemisia tabaci: Insights from individual-based
modelling” Andre Gergs, Angelika Weinhold, Elena Hettmann, Mariana
Durigan, Lokeshkumar Kadu, Jocelyn Kratchmer, Christian Marienhagen

This notebook is to document, run and evaluate IBM simulations. Steps
are as follow: 1. prepare settings for model simulation and parameter
input file parameters were derived from Andre Gergs. 2024. AmP Bemisia
tabaci, version 2024/03/19., at: https://www.bio.vu.nl/thb/deb/deblab/add\_my\_pet/entries\_web/Bemisia\_tabaci/Bemisia\_tabaci\_res.html

2. prepare environmental scenario input files
3. run IBM executable
4. read IBM output
5. do model statistics and plot output

## For model execution run all chunks top to bottom

Packages for the notebook


```
    knitr::opts_chunk$set(warning = FALSE, message = FALSE) 
    library(R.matlab)
    library (dplyr)
    library(processx)
    library(rvest)
```


1a. General model settings


```
     #--define simulation settings manually 
     MC_number=100    # number of Monte-Carlo repetitions, values > 1000 may lead to very long simulation times
     tmax<- 40 #maximum simulation time, d
     
     #Initial population
     inidist = 1    # 0/1 uniform initial distribution egg to adult (0) or normal ditribution stage and standard deviation (1)
     iniStage= 1      # mean stage for initial population
     iniSD=0.001       # coefficient of variation for body size in initial population, cm
     iniNo=50         # initial population size, #
     
     #adult migration
     immi_start_d=0  # start of adult imigration, d
     immi_end_d=tmax    # end of adult imigration, d
     immi_r=2        # adult imigration rate, #/d
     emi_start_d=1   # start of adult emigration, d
     emi_end_d=tmax   # end of adult emigration, d
     emi_r=0.1       # adult emigration rate, fraction/d, values 0...1; e.g. for 0.7, 70% of adults are randomly removed

     #overview table
     set_names = c("MC_number", "inidist", "iniStage", "iniSD", "iniNo", 
                   "immi_start_d", "immi_end_d", "immi_r", "emi_start_d", 
                   "emi_end_d", "emi_r", "tmax")
       set_values = c(MC_number,inidist, iniStage, iniSD, iniNo, 
                      immi_start_d, immi_end_d, immi_r, emi_start_d, 
                      emi_end_d, emi_r, tmax)
         set_description = c("number of Monte-Carlo repetitions","initial distribution, 0/1 (uniform/normal)", 
                             "mean stage for initial population (inidist = 1)", 
                             "coefficient of variation for body size in initial population (inidist = 1)", 
                             "initial population   size", "start of adult imigration", "end of adult imigration", 
                             "adult imigration rate", "start of adult emigration", "end of adult emigration", 
                             "adult emigration rate", "maximum simulation time")
         
    setting <- as.data.frame( cbind (set_names,set_values, set_description))
    names(setting) <- c("symbol", "value", "description")
    setting
```


1b. Create setting input file


```
 write.csv(setting, "set.csv", row.names=FALSE)
 print("- done -")
```


1c. Read DEB parameters from .mat (AmP) file


```
        myWD<-getwd()

        AmP<-readMat('results_Bemisia_tabaci.mat')
        mod   <- as.character( AmP$metaPar[rownames(AmP$metaPar)=="model"] ) #typified mdoel

  # primary DEB parameters
          z       <- as.numeric( AmP$par[rownames(AmP$par)=="z"])       # zoom factor
          F_m     <- as.numeric( AmP$par[rownames(AmP$par)=="F.m"])     # max spec searching rate
          kap_X   <- as.numeric( AmP$par[rownames(AmP$par)=="kap.X"])   # digestion efficiency of food to reserve
          kap_P   <- as.numeric( AmP$par[rownames(AmP$par)=="kap.P"])   # faecation efficiency of food to faeces
          v       <- as.numeric( AmP$par[rownames(AmP$par)=="v"])       # energy conductance
          del_M   <- as.numeric( AmP$par[rownames(AmP$par)=="del.M"])   # shape coefficient
          kappa   <- as.numeric( AmP$par[rownames(AmP$par)=="kap"])     # allocation fraction to soma
          kap_R   <- as.numeric( AmP$par[rownames(AmP$par)=="kap.R"])   # reproduction efficiency
          p_M     <- as.numeric( AmP$par[rownames(AmP$par)=="p.M"])     # volume specific somatic maintenance cost
          p_T     <- as.numeric( AmP$par[rownames(AmP$par)=="p.T"])     # surf-spec somatic maintenance cost
          kj      <- as.numeric( AmP$par[rownames(AmP$par)=="k.J"])     # maturity maintenance rate coefficient
          E_G     <- as.numeric( AmP$par[rownames(AmP$par)=="E.G"])     # volume-specific cost for structure
          E_Hb    <- as.numeric( AmP$par[rownames(AmP$par)=="E.Hb"])    # maturity at birth
          if(length(AmP$par[rownames(AmP$par)=="E.Hj"]) == 0) {E_Hj    <- NA #   check if parameter is available
            }else E_Hj    <- as.numeric( AmP$par[rownames(AmP$par)=="E.Hj"])    # maturity at metamorphosis
          E_Hp    <- as.numeric( AmP$par[rownames(AmP$par)=="E.Hp"])    # maturity at puberty
          ha      <- as.numeric( AmP$par[rownames(AmP$par)=="h.a"])     # Weibull aging acceleration
          sG      <- as.numeric( AmP$par[rownames(AmP$par)=="s.G"])     # Gompertz stress coefficient

  # compound parameters
          p_Am    <- z * p_M/ kappa     # surface area specific assimilation flux
          E_m     <- p_Am/v             # reserve capacity
          g       <- E_G/(kappa*E_m)    # energy investment ratio          
          km      <- p_M/E_G            # somatic maintenance rate coefficient  
          ubh     <- E_Hb/p_Am          # scaled maturity at birth
          uph     <- E_Hp/p_Am          # scaled maturity at puberty

  # temperature parameters
          T    <- 23+273.15
          T_ref <- as.numeric( AmP$par[rownames(AmP$par)=="T.ref"])  
          T_A   <- as.numeric(AmP$par[rownames(AmP$par)=="T.A"] )        #  Arrhenius temperature

    #list of parameter names and parameter values
          par_names = c("model", "z","F_m","kap_X", "kap_P", "v", "kappa", "kap_R", "p_M", "p_T", "kj", "E_G", "E_Hb", "E_Hj", "E_Hp", "ha", "sG", "T_A", "del_M")
          par_values = c(mod,    z, F_m,   kap_X,   kap_P,   v,   kappa,    kap_R,   p_M,   p_T,   kj,   E_G,   E_Hb,   E_Hj,   E_Hp,   ha,   sG,  T_A, del_M)
          
          par_description = c(
          "typified model",
          "zoom factor",
          "max. spec. searching rate",
          "digestion efficiency of food to reserve",
          "faecation efficiency of food to faeces",
          "energy conductance",
          "allocation fraction to soma",
          "reproduction efficiency",
          "vol.-spec. somatic maint",
          "surf.-spec. somatic maintenance cost",
          "maturity maint. rate coefficient",
          "spec. cost for structure",
          "maturity at birth",
          "maturity at metamorphosis",
          "maturity at puberty",
          "Weibull aging acceleration",
          "Gompertz stress coefficient",
          "Arrhenius temperature",
          "shape coefficient"
          )
  #Temperature related parameters
        if(length(AmP$par[rownames(AmP$par)=="T.AL"]) > 0) {          #   check if lower pars are available
           T_AL     <- as.numeric(AmP$par[rownames(AmP$par)=="T.AL"] )  #   lower Arrhenius temperature
           T_L  <- as.numeric(AmP$par[rownames(AmP$par)=="T.L"] )       #   lower boundary for Arrhenius temperature
            par_names = c(par_names, "T_AL", "T_L")
             par_values= c(par_values, T_AL, T_L )
               par_description = c(par_description, "lower Arrhenius temperature", "lower boundary for Arrhenius temperature")
        }
        
        if(length(AmP$par[rownames(AmP$par)=="T.AH"]) > 0) {          #   check if upper pars are available
          T_AH <-  as.numeric(AmP$par[rownames(AmP$par)=="T.AH"])     #     upper Arrhenius temperature
          T_H   <- as.numeric(AmP$par[rownames(AmP$par)=="T.H"])      #     upper boundary for Arrhenius temperature
           par_names = c(par_names, "T_AH", "T_H")
            par_values= c(par_values, T_AH, T_H )
             par_description = c(par_description, "upper Arrhenius temperature", "upper boundary for Arrhenius temperature")
        }

  #stresses for insect life stages following Dyar's Law 
      i=0
      repeat {
        i = i +1
        p = paste ("s",i, sep=".")
        if(length(AmP$par[rownames(AmP$par)==p]) == 0) {break
         } else {
           assign(paste ("s",i, sep="_") ,as.numeric(AmP$par[rownames(AmP$par)==p])) # stress at instar
            par_names = c(par_names, paste("s",i, sep="_"))
              par_values= c(par_values, as.numeric(AmP$par[rownames(AmP$par)==p]))
                par_description = c(par_description, paste ("stress at instar",i, sep=" "))
             }
      }
      stages = i # number of larval stages
       par_names = c(par_names,"stages")
        par_values= c(par_values, stages)
         par_description = c(par_description, "number of larval stages")
  
  #Create and save paamter table
      par_table <- as.data.frame( cbind (par_names,par_values, par_description))
      names(par_table) <- c("symbol", "value", "description")
     
      par_table
```


1d. Read implied properties from .html file (AmP) and define
additional parameters


```
# Read the Parameters and implied properties from report html file
 reportpage <- read_html("report_Bemisia_tabaci.html")
  table_node <- html_node(reportpage, "table") # Extract the table node and convert it to a data frame
    AmP2 <- html_table(table_node, fill = TRUE, header = FALSE)
      AmP2 <- AmP2  [3:nrow(AmP2 ),]
        colnames(AmP2 )<- c("symbol", "units", "value", "C", "func_resp", "description")
  
  E_0      <- as.numeric( AmP2$value[AmP2$symbol=="E_0"])     # J, initial reserve
  L_b      <- as.numeric( AmP2$value[AmP2$symbol=="L_b"])     # cm, structural length at birth at f=1
  L_p      <- as.numeric( AmP2$value[AmP2$symbol=="L_p"])     # cm, structural length at puberty at f=1
  f   <- 1        # -, scaled functional response
  
 
  # background hazard during larval development
     h_b    <-  0.0423
  
  #add parameters to vectors
  par_names=NULL; par_values=NULL;par_description=NULL
      par_names = c(par_names,"E_0", "L_b", "L_p", "f", "h_b")
        par_values= c(par_values, E_0, L_b, L_p, f, h_b)
           par_description = c(par_description, "initial reserve", "structural length at birth", "structural length at puberty", 
                               "scaled functional response", "background hazard during larval development")
  
  par_table2 <- as.data.frame( cbind (par_names,par_values, par_description))
  names(par_table2) <- c("symbol", "value", "description")
  par_table2
```


1e. Read implied properties from .html file (AmP) and define
additional parameters


```
# assign parameter values
kd <-   0.08503   #dominant rate constant
zb <-   0.003969    #threshold for effects
bb <-   9.482       #effect strength energy budget
kk <-   0.25        #strength lethal effects

#add parameters to vectors
  par_names=NULL; par_values=NULL;par_description=NULL
      par_names = c(par_names,"kd", "zb", "bb", "kk")
        par_values= c(par_values, kd, zb, bb, kk)
           par_description = c(par_description, "dominant rate constant", "threshold for effects", "effect strength energy budget", 
                               "strength lethal effects")
  
  par_table3 <- as.data.frame( cbind (par_names,par_values, par_description))
  names(par_table3) <- c("symbol", "value", "description")
  par_table3
```


1f. Create parameter input file


```
 par_tables<- rbind(par_table, par_table2, par_table3)
 write.csv(par_tables, "pars.csv", row.names=FALSE)
 print("- done -")
```


2a. Create temperature scenario


```
  #create constant temperature scenarios----------------------------------------
      times<-seq(1:tmax) #time vector
      scenarios<-1 #number of scenarios to be created
      
      temperatures <- matrix(data = times, nrow = length(times), ncol = scenarios+1) # results matrix
      for (x in 1:scenarios){ 
         for (t in 1:length(times)){
           #set constant temperature, alternatively, create your own trajectory
           temperatures [t,x+1] <- 26 
         }
        
        #plot temperature
         plot(temperatures [,1], temperatures [,x+1], type = "l",  lwd=3,  col = "lightblue4", ylab = "Temperature [C]", xlab = "Time [d]", 
              cex.axis=1.2, cex.lab =1.2, ylim = c(15,40), xlim=c(0,35),  bty="n"  )  #  
      }
      write.csv(temperatures, "temperature_scenarios.csv", row.names= FALSE)
```


2b. Create exposure scenario


```
  #scenario specifications
    timings = c(1, 10) #timing (d) of application(s), add timings as need
      treatments = c(24) #Application rate for each timing, g/ha
    
    GAP <- matrix(NaN,  nrow = length(timings), ncol = 3)
    colnames(GAP) <- c("Treatment, #", "Timing, d", "Rate, g/ha")

  #Parameters for temperature correction and half life (here: cotton) 
    T_ref   <-20  + 273.15 #reference temperature [C]
    T_A_cot <-3.336e+04    #Arrhenius temperature
    T_AH_cot <- 4.392e+04 # upper Arrhenius temperature
    T_H_cot <- 301.6     # upper boundary temperature
    k=0.042  #decay parameter related to product half life
    
  # run through temperature scenarios (number of temperature scenarios need to match the number of exposure scenarios)
    exposures <- matrix(data = NA, nrow = length(times), ncol = scenarios+1) # results matrix
    exposures [,1]<-times
      
    for (x in 1:scenarios){  
      
       #specify treatment series
            Treat_series     <-rep(c(0), length(times) )
            Treat_series [timings] <- treatments
            Conc <- Treat_series[1]
            exposures [1,x+1] <- Conc  
        
        #exposure for each temperature scenario
            for (t in 2:length(times)){ 
            T<-temperatures[t,x+1]+ 273.15  
              s_A = exp(T_A_cot/ T_ref - T_A_cot / T)  
                s_H_ratio = (1 + exp(T_AH_cot/ T_H_cot - T_AH_cot / T_ref)) / (1 + exp(T_AH_cot/ T_H_cot - T_AH_cot / T    ))
                  f_T = s_A * (T >= T_ref) * s_H_ratio
          
            Conc <- Conc * exp(-k * f_T) + Treat_series[t]                                 
            exposures [t,x+1] <- round (Conc,3)   
            }# loop time
       
        #plot exposures
        plot(exposures [,1], exposures [,x+1], type = "l",  lwd=3,  col = "darkorange2", ylab = "Concentration [g/ha]", xlab = "Time [d]", 
            main =  paste ("Scenario ", x), cex.axis=1, cex.lab =1,    bty="n", xlim=c(0,40), ylim = c(0,25) )  #
     }
        
    write.csv(exposures, "exposure_scenarios.csv", row.names= FALSE)
```


3. Execute the DEBibm executable


```
processx::run('DEBibm.exe')
print("- done -")
```


4 & 5a. Calculate means and quantiles; make figures comparing
total population sizes for untreated control and treatment.


```
MCmax<-paste ("MC_", MC_number, sep="" ) #maximum number of MC simulations for calculation of means and quantiles 

#--loop scenarios---------------------------------------------------------------------------
      for (x in 1:scenarios) {
      filename <- paste ("results/scenario_", x, ".csv", sep="" )
      output<- as.data.frame( read.csv(filename, header = TRUE))
      
      #--calculate mean and quantiles-------------------------------------------------
          output<-all_of(output %>%
          rowwise() %>%
          mutate( 
            mean_value = mean(c_across(MC_1:MCmax)),
            Q_1 = quantile(c_across(MC_1:MCmax), probs = c(0.025)),
            Q_2 = quantile(c_across(MC_1:MCmax), probs = c(0.5)),
            Q_3 = quantile(c_across(MC_1:MCmax), probs = c(0.975)),
           ))
      output_sum <-output[, colnames(output) %in% c("group","subpopulation","day", "mean_value", "Q_1", "Q_2", "Q_3")]
      
      #--quantiles separated by Control and treatment---------------------------------
        ctrl_sum <- output_sum[output_sum$group == "control", ] 
        treat_sum <- output_sum[output_sum$group == "treatment", ] 
      
      #--quantiles of total counts separated by Control and treatment-----------------
        ctrl_totals <- ctrl_sum[ctrl_sum$subpopulation== "total", ]  
        treat_totals <- treat_sum[treat_sum$subpopulation== "total", ] 
      
      #--plot population dynamics-----------------------------------------------------
        col_ctr_L<-"lightblue4"; col_ctr_P<-"lightblue3"; col_T_L <-"darkorange2"; col_T_P <- "orange"
        plot(ctrl_totals$day, ctrl_totals$mean_value, type = "l",  lwd=2, ylab = "Population size [#]", 
             xlab = "Time [d]", cex.axis=1, cex.lab =1,    bty="n", ylim=c(0,max(ctrl_totals$Q_3)) )  #
            polygon(c(ctrl_totals$day, rev(ctrl_totals$day)), c(ctrl_totals$Q_3 ,rev(ctrl_totals$Q_1)), border = NA , col = col_ctr_P)
              polygon(c(treat_totals$day, rev(treat_totals$day)), c(treat_totals$Q_3 ,rev(treat_totals$Q_1)), border = NA , col = col_T_P)
                lines(ctrl_totals$day, ctrl_totals$mean_value, type = "l",lwd=3, col = col_ctr_L)
                    lines(treat_totals$day, treat_totals$mean_value, type = "l",lwd=3, col = col_T_L) 
} #end loop scenarios
```


5b.Histograms for population demography based on means of Monte-Carlo
simulations


```
stages=4
#--plot settings
#choose plot colors according to number of stages
plot_col <-c("orange","lightblue4", "lightblue3","lightblue2","lightblue1","black")

#scale y-axis of treatment plot same as control?
ctrl_scale <- TRUE #TRUE/FALSE

#--loop scenarios---------------------------------------------------------------------------
  for (x in 1:scenarios) {
      filename <- paste ("results/scenario_", x, ".csv", sep="" )
      output<- as.data.frame( read.csv(filename, header = TRUE))

      #--calculate means
          output<-all_of(output %>%
          rowwise() %>%
          mutate(
            mean_value = mean(c_across(MC_1:MCmax)), ))
          output_sum <-output[, colnames(output) %in% c("group","subpopulation","day", "mean_value")]

      #--summarize data and make histograms
          par(mfrow = c(1, 2))
          for (i in 1:2) {
            #--quantiles for Control and treatment
                if (i==1) {
                    #filter for control data
                    instars_sum <- output_sum[output_sum$group == "control", ]
                    #main title for plot
                    title<- "Untreated control"
                    #search for maximum value for ylim
                    y_max <- signif( max(output_sum[output_sum$subpopulation == "total", 4]), digits = 1)

                } else {
                    #filter for treatment data
                    instars_sum <- output_sum[output_sum$group == "treatment", ]
                    #main title for plot
                    title<- "Treatment"

                    #search for maximum value for ylim in treatment
                    if (ctrl_scale == FALSE) y_max <- signif( max(output_sum[output_sum$group == "treatment", 4]), digits = 1)
                    }

            #--matrix for different stages
                day <- data.frame( day = c(1:tmax))
                df_instars<-as.data.frame (c(1:tmax))
                # eggs
                  L <-as.data.frame (instars_sum[instars_sum$subpopulation == "eggs", 4] )
                  df_instars<- cbind(df_instars,L)
                    colnames(df_instars)<-c("day", "eggs")
                # larval stages
                for (i in 1:stages) {
                    s<-paste ("instar",i, sep = "")
                    L <-as.data.frame (instars_sum[instars_sum$subpopulation == s, 4] )
                    colnames(L )<-s
                    df_instars<- cbind(df_instars,L)
                }
                # adults
                L <-as.data.frame (instars_sum[instars_sum$subpopulation == "adults", 4] )
                colnames(L )<-"adults"
                df_instars<- cbind(df_instars,L)

         #--histograms
              dta<-t(as.matrix(df_instars[,2:(stages+3)]))
                barplot(dta,
                main = title,
                xlab = "Time [d]",
                ylab = "Count [#]",
                cex.axis=1, cex.lab =1,
                ylim = c(0, y_max),
                #ylim = c(0, 1000),
                axes = TRUE,
                legend.text = rownames(dta),
                args.legend = list(x = "topleft", inset = c(0.05, 0)),
                col=plot_col,
                names=df_instars[,1]
                )
      } #loop control/treatment

}#loop scenarios
```


5c. Plot efficacy


```
 #--efficacy quantiles for total counts
    efficacy_totals<-treat_totals
      efficacy_totals$Q_1<-1-efficacy_totals$Q_1/ctrl_totals$Q_2
        efficacy_totals$Q_2<-1-efficacy_totals$Q_2/ctrl_totals$Q_2
          efficacy_totals$Q_3<-1-efficacy_totals$Q_3/ctrl_totals$Q_2
          
plot(efficacy_totals$day, efficacy_totals$Q_2, type = "l",  lwd=2, 
     ylab = "Efficacy [-]", main =  "Total efficacy", cex.main =1.2 ,
         xlab = "Time [d]" , cex.axis=1.2, cex.lab =1.2,    bty="n", ylim=c(0 ,1.05) )  #
            polygon(c(efficacy_totals$day, rev(efficacy_totals$day)), 
                    c(efficacy_totals$Q_3 ,rev(efficacy_totals$Q_1)), 
                      border = NA , col = "lightblue3") 
               lines(efficacy_totals$day, efficacy_totals$Q_2, type = "l",lwd=3, col = "lightblue4")
```

LS0tDQp0aXRsZTogIlJ1biBJQk0gZXhlIg0Kb3V0cHV0OiBodG1sX25vdGVib29rDQogIA0KZWRpdG9yX29wdGlvbnM6IA0KICBjaHVua19vdXRwdXRfdHlwZTogaW5saW5lDQogDQotLS0NClN1cHBsZW1lbnRhcnkgSW5mb3JtYXRpb24gdG8gDQoiT3B0aW1pemluZyBjb250cm9sIHN0cmF0ZWdpZXMgZm9yIHRoZSBjb3R0b24gd2hpdGVmbHkgQmVtaXNpYSB0YWJhY2k6IEluc2lnaHRzIGZyb20gaW5kaXZpZHVhbC1iYXNlZCBtb2RlbGxpbmciDQpBbmRyZSBHZXJncywgQW5nZWxpa2EgV2VpbmhvbGQsIEVsZW5hIEhldHRtYW5uLCBNYXJpYW5hIER1cmlnYW4sIExva2VzaGt1bWFyIEthZHUsIEpvY2VseW4gS3JhdGNobWVyLCBDaHJpc3RpYW4gTWFyaWVuaGFnZW4NCg0KDQpUaGlzIG5vdGVib29rIGlzIHRvIGRvY3VtZW50LCBydW4gYW5kIGV2YWx1YXRlIElCTSBzaW11bGF0aW9ucy4gDQpTdGVwcyBhcmUgYXMgZm9sbG93Og0KMS4gIHByZXBhcmUgc2V0dGluZ3MgZm9yIG1vZGVsIHNpbXVsYXRpb24gYW5kIHBhcmFtZXRlciBpbnB1dCBmaWxlDQogICAgcGFyYW1ldGVycyB3ZXJlIGRlcml2ZWQgZnJvbSBBbmRyZSBHZXJncy4gMjAyNC4gQW1QIEJlbWlzaWEgdGFiYWNpLCB2ZXJzaW9uIDIwMjQvMDMvMTkuLCBhdDoNCiAgICBodHRwczovL3d3dy5iaW8udnUubmwvdGhiL2RlYi9kZWJsYWIvYWRkX215X3BldC9lbnRyaWVzX3dlYi9CZW1pc2lhX3RhYmFjaS9CZW1pc2lhX3RhYmFjaV9yZXMuaHRtbA0KDQoyLiAgcHJlcGFyZSBlbnZpcm9ubWVudGFsIHNjZW5hcmlvIGlucHV0IGZpbGVzDQoNCjMuICBydW4gSUJNIGV4ZWN1dGFibGUNCg0KNC4gIHJlYWQgSUJNIG91dHB1dA0KDQo1LiAgZG8gbW9kZWwgc3RhdGlzdGljcyBhbmQgcGxvdCBvdXRwdXQNCg0KRm9yIG1vZGVsIGV4ZWN1dGlvbiBydW4gYWxsIGNodW5rcyB0b3AgdG8gYm90dG9tDQotLQ0KDQpQYWNrYWdlcyBmb3IgdGhlIG5vdGVib29rDQpgYGB7cn0NCiAgICBrbml0cjo6b3B0c19jaHVuayRzZXQod2FybmluZyA9IEZBTFNFLCBtZXNzYWdlID0gRkFMU0UpIA0KICAgIGxpYnJhcnkoUi5tYXRsYWIpDQogICAgbGlicmFyeSAoZHBseXIpDQogICAgbGlicmFyeShwcm9jZXNzeCkNCiAgICBsaWJyYXJ5KHJ2ZXN0KQ0KYGBgDQoNCg0KMWEuIEdlbmVyYWwgbW9kZWwgc2V0dGluZ3MNCg0KYGBge3J9DQogICAgICMtLWRlZmluZSBzaW11bGF0aW9uIHNldHRpbmdzIG1hbnVhbGx5IA0KICAgICBNQ19udW1iZXI9MTAwICAgICMgbnVtYmVyIG9mIE1vbnRlLUNhcmxvIHJlcGV0aXRpb25zLCB2YWx1ZXMgPiAxMDAwIG1heSBsZWFkIHRvIHZlcnkgbG9uZyBzaW11bGF0aW9uIHRpbWVzDQogICAgIHRtYXg8LSA0MCAjbWF4aW11bSBzaW11bGF0aW9uIHRpbWUsIGQNCiAgICAgDQogICAgICNJbml0aWFsIHBvcHVsYXRpb24NCiAgICAgaW5pZGlzdCA9IDEgICAgIyAwLzEgdW5pZm9ybSBpbml0aWFsIGRpc3RyaWJ1dGlvbiBlZ2cgdG8gYWR1bHQgKDApIG9yIG5vcm1hbCBkaXRyaWJ1dGlvbiBzdGFnZSBhbmQgc3RhbmRhcmQgZGV2aWF0aW9uICgxKQ0KICAgICBpbmlTdGFnZT0gMSAgICAgICMgbWVhbiBzdGFnZSBmb3IgaW5pdGlhbCBwb3B1bGF0aW9uDQogICAgIGluaVNEPTAuMDAxICAgICAgICMgY29lZmZpY2llbnQgb2YgdmFyaWF0aW9uIGZvciBib2R5IHNpemUgaW4gaW5pdGlhbCBwb3B1bGF0aW9uLCBjbQ0KICAgICBpbmlObz01MCAgICAgICAgICMgaW5pdGlhbCBwb3B1bGF0aW9uIHNpemUsICMNCiAgICAgDQogICAgICNhZHVsdCBtaWdyYXRpb24NCiAgICAgaW1taV9zdGFydF9kPTAgICMgc3RhcnQgb2YgYWR1bHQgaW1pZ3JhdGlvbiwgZA0KICAgICBpbW1pX2VuZF9kPXRtYXggICAgIyBlbmQgb2YgYWR1bHQgaW1pZ3JhdGlvbiwgZA0KICAgICBpbW1pX3I9MiAgICAgICAgIyBhZHVsdCBpbWlncmF0aW9uIHJhdGUsICMvZA0KICAgICBlbWlfc3RhcnRfZD0xICAgIyBzdGFydCBvZiBhZHVsdCBlbWlncmF0aW9uLCBkDQogICAgIGVtaV9lbmRfZD10bWF4ICAgIyBlbmQgb2YgYWR1bHQgZW1pZ3JhdGlvbiwgZA0KICAgICBlbWlfcj0wLjEgICAgICAgIyBhZHVsdCBlbWlncmF0aW9uIHJhdGUsIGZyYWN0aW9uL2QsIHZhbHVlcyAwLi4uMTsgZS5nLiBmb3IgMC43LCA3MCUgb2YgYWR1bHRzIGFyZSByYW5kb21seSByZW1vdmVkDQoNCiAgICAgI292ZXJ2aWV3IHRhYmxlDQogICAgIHNldF9uYW1lcyA9IGMoIk1DX251bWJlciIsICJpbmlkaXN0IiwgImluaVN0YWdlIiwgImluaVNEIiwgImluaU5vIiwgDQogICAgICAgICAgICAgICAgICAgImltbWlfc3RhcnRfZCIsICJpbW1pX2VuZF9kIiwgImltbWlfciIsICJlbWlfc3RhcnRfZCIsIA0KICAgICAgICAgICAgICAgICAgICJlbWlfZW5kX2QiLCAiZW1pX3IiLCAidG1heCIpDQogICAgICAgc2V0X3ZhbHVlcyA9IGMoTUNfbnVtYmVyLGluaWRpc3QsIGluaVN0YWdlLCBpbmlTRCwgaW5pTm8sIA0KICAgICAgICAgICAgICAgICAgICAgIGltbWlfc3RhcnRfZCwgaW1taV9lbmRfZCwgaW1taV9yLCBlbWlfc3RhcnRfZCwgDQogICAgICAgICAgICAgICAgICAgICAgZW1pX2VuZF9kLCBlbWlfciwgdG1heCkNCiAgICAgICAgIHNldF9kZXNjcmlwdGlvbiA9IGMoIm51bWJlciBvZiBNb250ZS1DYXJsbyByZXBldGl0aW9ucyIsImluaXRpYWwgZGlzdHJpYnV0aW9uLCAwLzEgKHVuaWZvcm0vbm9ybWFsKSIsIA0KICAgICAgICAgICAgICAgICAgICAgICAgICAgICAibWVhbiBzdGFnZSBmb3IgaW5pdGlhbCBwb3B1bGF0aW9uIChpbmlkaXN0ID0gMSkiLCANCiAgICAgICAgICAgICAgICAgICAgICAgICAgICAgImNvZWZmaWNpZW50IG9mIHZhcmlhdGlvbiBmb3IgYm9keSBzaXplIGluIGluaXRpYWwgcG9wdWxhdGlvbiAoaW5pZGlzdCA9IDEpIiwgDQogICAgICAgICAgICAgICAgICAgICAgICAgICAgICJpbml0aWFsIHBvcHVsYXRpb24gICBzaXplIiwgInN0YXJ0IG9mIGFkdWx0IGltaWdyYXRpb24iLCAiZW5kIG9mIGFkdWx0IGltaWdyYXRpb24iLCANCiAgICAgICAgICAgICAgICAgICAgICAgICAgICAgImFkdWx0IGltaWdyYXRpb24gcmF0ZSIsICJzdGFydCBvZiBhZHVsdCBlbWlncmF0aW9uIiwgImVuZCBvZiBhZHVsdCBlbWlncmF0aW9uIiwgDQogICAgICAgICAgICAgICAgICAgICAgICAgICAgICJhZHVsdCBlbWlncmF0aW9uIHJhdGUiLCAibWF4aW11bSBzaW11bGF0aW9uIHRpbWUiKQ0KICAgICAgICAgDQogICAgc2V0dGluZyA8LSBhcy5kYXRhLmZyYW1lKCBjYmluZCAoc2V0X25hbWVzLHNldF92YWx1ZXMsIHNldF9kZXNjcmlwdGlvbikpDQogICAgbmFtZXMoc2V0dGluZykgPC0gYygic3ltYm9sIiwgInZhbHVlIiwgImRlc2NyaXB0aW9uIikNCiAgICBzZXR0aW5nIA0KYGBgDQoNCg0KMWIuIENyZWF0ZSBzZXR0aW5nIGlucHV0IGZpbGUNCg0KYGBge3J9DQogd3JpdGUuY3N2KHNldHRpbmcsICJzZXQuY3N2Iiwgcm93Lm5hbWVzPUZBTFNFKQ0KIHByaW50KCItIGRvbmUgLSIpDQpgYGANCg0KDQoxYy4gUmVhZCBERUIgcGFyYW1ldGVycyBmcm9tIC5tYXQgKEFtUCkgZmlsZQ0KDQpgYGB7ciBlY2hvPVRSVUUsIHBhZ2VkLnByaW50PVRSVUV9DQogICAgICAgIG15V0Q8LWdldHdkKCkNCg0KICAgICAgICBBbVA8LXJlYWRNYXQoJ3Jlc3VsdHNfQmVtaXNpYV90YWJhY2kubWF0JykNCiAgICAgICAgbW9kICAgPC0gYXMuY2hhcmFjdGVyKCBBbVAkbWV0YVBhcltyb3duYW1lcyhBbVAkbWV0YVBhcik9PSJtb2RlbCJdICkgI3R5cGlmaWVkIG1kb2VsDQoNCiAgIyBwcmltYXJ5IERFQiBwYXJhbWV0ZXJzDQogICAgICAgICAgeiAgICAgICA8LSBhcy5udW1lcmljKCBBbVAkcGFyW3Jvd25hbWVzKEFtUCRwYXIpPT0ieiJdKSAgICAgICAjIHpvb20gZmFjdG9yDQogICAgICAgICAgRl9tICAgICA8LSBhcy5udW1lcmljKCBBbVAkcGFyW3Jvd25hbWVzKEFtUCRwYXIpPT0iRi5tIl0pICAgICAjIG1heCBzcGVjIHNlYXJjaGluZyByYXRlDQogICAgICAgICAga2FwX1ggICA8LSBhcy5udW1lcmljKCBBbVAkcGFyW3Jvd25hbWVzKEFtUCRwYXIpPT0ia2FwLlgiXSkgICAjIGRpZ2VzdGlvbiBlZmZpY2llbmN5IG9mIGZvb2QgdG8gcmVzZXJ2ZQ0KICAgICAgICAgIGthcF9QICAgPC0gYXMubnVtZXJpYyggQW1QJHBhcltyb3duYW1lcyhBbVAkcGFyKT09ImthcC5QIl0pICAgIyBmYWVjYXRpb24gZWZmaWNpZW5jeSBvZiBmb29kIHRvIGZhZWNlcw0KICAgICAgICAgIHYgICAgICAgPC0gYXMubnVtZXJpYyggQW1QJHBhcltyb3duYW1lcyhBbVAkcGFyKT09InYiXSkgICAgICAgIyBlbmVyZ3kgY29uZHVjdGFuY2UNCiAgICAgICAgICBkZWxfTSAgIDwtIGFzLm51bWVyaWMoIEFtUCRwYXJbcm93bmFtZXMoQW1QJHBhcik9PSJkZWwuTSJdKSAgICMgc2hhcGUgY29lZmZpY2llbnQNCiAgICAgICAgICBrYXBwYSAgIDwtIGFzLm51bWVyaWMoIEFtUCRwYXJbcm93bmFtZXMoQW1QJHBhcik9PSJrYXAiXSkgICAgICMgYWxsb2NhdGlvbiBmcmFjdGlvbiB0byBzb21hDQogICAgICAgICAga2FwX1IgICA8LSBhcy5udW1lcmljKCBBbVAkcGFyW3Jvd25hbWVzKEFtUCRwYXIpPT0ia2FwLlIiXSkgICAjIHJlcHJvZHVjdGlvbiBlZmZpY2llbmN5DQogICAgICAgICAgcF9NICAgICA8LSBhcy5udW1lcmljKCBBbVAkcGFyW3Jvd25hbWVzKEFtUCRwYXIpPT0icC5NIl0pICAgICAjIHZvbHVtZSBzcGVjaWZpYyBzb21hdGljIG1haW50ZW5hbmNlIGNvc3QNCiAgICAgICAgICBwX1QgICAgIDwtIGFzLm51bWVyaWMoIEFtUCRwYXJbcm93bmFtZXMoQW1QJHBhcik9PSJwLlQiXSkgICAgICMgc3VyZi1zcGVjIHNvbWF0aWMgbWFpbnRlbmFuY2UgY29zdA0KICAgICAgICAgIGtqICAgICAgPC0gYXMubnVtZXJpYyggQW1QJHBhcltyb3duYW1lcyhBbVAkcGFyKT09ImsuSiJdKSAgICAgIyBtYXR1cml0eSBtYWludGVuYW5jZSByYXRlIGNvZWZmaWNpZW50DQogICAgICAgICAgRV9HICAgICA8LSBhcy5udW1lcmljKCBBbVAkcGFyW3Jvd25hbWVzKEFtUCRwYXIpPT0iRS5HIl0pICAgICAjIHZvbHVtZS1zcGVjaWZpYyBjb3N0IGZvciBzdHJ1Y3R1cmUNCiAgICAgICAgICBFX0hiICAgIDwtIGFzLm51bWVyaWMoIEFtUCRwYXJbcm93bmFtZXMoQW1QJHBhcik9PSJFLkhiIl0pICAgICMgbWF0dXJpdHkgYXQgYmlydGgNCiAgICAgICAgICBpZihsZW5ndGgoQW1QJHBhcltyb3duYW1lcyhBbVAkcGFyKT09IkUuSGoiXSkgPT0gMCkge0VfSGogICAgPC0gTkEgIyAgIGNoZWNrIGlmIHBhcmFtZXRlciBpcyBhdmFpbGFibGUNCiAgICAgICAgICAgIH1lbHNlIEVfSGogICAgPC0gYXMubnVtZXJpYyggQW1QJHBhcltyb3duYW1lcyhBbVAkcGFyKT09IkUuSGoiXSkgICAgIyBtYXR1cml0eSBhdCBtZXRhbW9ycGhvc2lzDQogICAgICAgICAgRV9IcCAgICA8LSBhcy5udW1lcmljKCBBbVAkcGFyW3Jvd25hbWVzKEFtUCRwYXIpPT0iRS5IcCJdKSAgICAjIG1hdHVyaXR5IGF0IHB1YmVydHkNCiAgICAgICAgICBoYSAgICAgIDwtIGFzLm51bWVyaWMoIEFtUCRwYXJbcm93bmFtZXMoQW1QJHBhcik9PSJoLmEiXSkgICAgICMgV2VpYnVsbCBhZ2luZyBhY2NlbGVyYXRpb24NCiAgICAgICAgICBzRyAgICAgIDwtIGFzLm51bWVyaWMoIEFtUCRwYXJbcm93bmFtZXMoQW1QJHBhcik9PSJzLkciXSkgICAgICMgR29tcGVydHogc3RyZXNzIGNvZWZmaWNpZW50DQoNCiAgIyBjb21wb3VuZCBwYXJhbWV0ZXJzDQogICAgICAgICAgcF9BbSAgICA8LSB6ICogcF9NLyBrYXBwYSAgICAgIyBzdXJmYWNlIGFyZWEgc3BlY2lmaWMgYXNzaW1pbGF0aW9uIGZsdXgNCiAgICAgICAgICBFX20gICAgIDwtIHBfQW0vdiAgICAgICAgICAgICAjIHJlc2VydmUgY2FwYWNpdHkNCiAgICAgICAgICBnICAgICAgIDwtIEVfRy8oa2FwcGEqRV9tKSAgICAjIGVuZXJneSBpbnZlc3RtZW50IHJhdGlvICAgICAgICAgIA0KICAgICAgICAgIGttICAgICAgPC0gcF9NL0VfRyAgICAgICAgICAgICMgc29tYXRpYyBtYWludGVuYW5jZSByYXRlIGNvZWZmaWNpZW50ICANCiAgICAgICAgICB1YmggICAgIDwtIEVfSGIvcF9BbSAgICAgICAgICAjIHNjYWxlZCBtYXR1cml0eSBhdCBiaXJ0aA0KICAgICAgICAgIHVwaCAgICAgPC0gRV9IcC9wX0FtICAgICAgICAgICMgc2NhbGVkIG1hdHVyaXR5IGF0IHB1YmVydHkNCg0KICAjIHRlbXBlcmF0dXJlIHBhcmFtZXRlcnMNCiAgICAgICAgICBUICAgIDwtIDIzKzI3My4xNQ0KICAgICAgICAgIFRfcmVmIDwtIGFzLm51bWVyaWMoIEFtUCRwYXJbcm93bmFtZXMoQW1QJHBhcik9PSJULnJlZiJdKSAgDQogICAgICAgICAgVF9BIAk8LSBhcy5udW1lcmljKEFtUCRwYXJbcm93bmFtZXMoQW1QJHBhcik9PSJULkEiXSApCSAgICAgIyAJQXJyaGVuaXVzIHRlbXBlcmF0dXJlDQoNCg0KICAgICNsaXN0IG9mIHBhcmFtZXRlciBuYW1lcyBhbmQgcGFyYW1ldGVyIHZhbHVlcw0KICAgICAgICAgIHBhcl9uYW1lcyA9IGMoIm1vZGVsIiwgInoiLCJGX20iLCJrYXBfWCIsICJrYXBfUCIsICJ2IiwgImthcHBhIiwgImthcF9SIiwgInBfTSIsICJwX1QiLCAia2oiLCAiRV9HIiwgIkVfSGIiLCAiRV9IaiIsICJFX0hwIiwgImhhIiwgInNHIiwgIlRfQSIsICJkZWxfTSIpDQogICAgICAgICAgcGFyX3ZhbHVlcyA9IGMobW9kLCAgICB6LCBGX20sICAga2FwX1gsICAga2FwX1AsICAgdiwgICBrYXBwYSwgICAga2FwX1IsICAgcF9NLCAgIHBfVCwgICBraiwgICBFX0csICAgRV9IYiwgICBFX0hqLCAgIEVfSHAsICAgaGEsICAgc0csICBUX0EsIGRlbF9NKQ0KICAgICAgICAgIA0KICAgICAgICAgIHBhcl9kZXNjcmlwdGlvbiA9IGMoDQogICAgICAgICAgInR5cGlmaWVkIG1vZGVsIiwNCiAgICAgICAgICAiem9vbSBmYWN0b3IiLA0KICAgICAgICAgICJtYXguIHNwZWMuIHNlYXJjaGluZyByYXRlIiwNCiAgICAgICAgICAiZGlnZXN0aW9uIGVmZmljaWVuY3kgb2YgZm9vZCB0byByZXNlcnZlIiwNCiAgICAgICAgICAiZmFlY2F0aW9uIGVmZmljaWVuY3kgb2YgZm9vZCB0byBmYWVjZXMiLA0KICAgICAgICAgICJlbmVyZ3kgY29uZHVjdGFuY2UiLA0KICAgICAgICAgICJhbGxvY2F0aW9uIGZyYWN0aW9uIHRvIHNvbWEiLA0KICAgICAgICAgICJyZXByb2R1Y3Rpb24gZWZmaWNpZW5jeSIsDQogICAgICAgICAgInZvbC4tc3BlYy4gc29tYXRpYyBtYWludCIsDQogICAgICAgICAgInN1cmYuLXNwZWMuIHNvbWF0aWMgbWFpbnRlbmFuY2UgY29zdCIsDQogICAgICAgICAgIm1hdHVyaXR5IG1haW50LiByYXRlIGNvZWZmaWNpZW50IiwNCiAgICAgICAgICAic3BlYy4gY29zdCBmb3Igc3RydWN0dXJlIiwNCiAgICAgICAgICAibWF0dXJpdHkgYXQgYmlydGgiLA0KICAgICAgICAgICJtYXR1cml0eSBhdCBtZXRhbW9ycGhvc2lzIiwNCiAgICAgICAgICAibWF0dXJpdHkgYXQgcHViZXJ0eSIsDQogICAgICAgICAgIldlaWJ1bGwgYWdpbmcgYWNjZWxlcmF0aW9uIiwNCiAgICAgICAgICAiR29tcGVydHogc3RyZXNzIGNvZWZmaWNpZW50IiwNCiAgICAgICAgICAiQXJyaGVuaXVzIHRlbXBlcmF0dXJlIiwNCiAgICAgICAgICAic2hhcGUgY29lZmZpY2llbnQiDQogICAgICAgICAgKQ0KICAjVGVtcGVyYXR1cmUgcmVsYXRlZCBwYXJhbWV0ZXJzDQogICAgICAgIGlmKGxlbmd0aChBbVAkcGFyW3Jvd25hbWVzKEFtUCRwYXIpPT0iVC5BTCJdKSA+IDApIHsgICAgICAgICAgIyAgIGNoZWNrIGlmIGxvd2VyIHBhcnMgYXJlIGF2YWlsYWJsZQ0KICAgICAgICAgICBUX0FMIAk8LSBhcy5udW1lcmljKEFtUCRwYXJbcm93bmFtZXMoQW1QJHBhcik9PSJULkFMIl0gKQkjIAlsb3dlciBBcnJoZW5pdXMgdGVtcGVyYXR1cmUNCiAgICAgICAgICAgVF9MIAk8LSBhcy5udW1lcmljKEFtUCRwYXJbcm93bmFtZXMoQW1QJHBhcik9PSJULkwiXSApCSAgICAjIAlsb3dlciBib3VuZGFyeSBmb3IgQXJyaGVuaXVzIHRlbXBlcmF0dXJlDQogICAgICAgICAgICBwYXJfbmFtZXMgPSBjKHBhcl9uYW1lcywgIlRfQUwiLCAiVF9MIikNCiAgICAgICAgICAgICBwYXJfdmFsdWVzPSBjKHBhcl92YWx1ZXMsIFRfQUwsIFRfTCApDQogICAgICAgICAgICAgICBwYXJfZGVzY3JpcHRpb24gPSBjKHBhcl9kZXNjcmlwdGlvbiwgImxvd2VyIEFycmhlbml1cyB0ZW1wZXJhdHVyZSIsICJsb3dlciBib3VuZGFyeSBmb3IgQXJyaGVuaXVzIHRlbXBlcmF0dXJlIikNCiAgICAgICAgfQ0KICAgICAgICANCiAgICAgICAgaWYobGVuZ3RoKEFtUCRwYXJbcm93bmFtZXMoQW1QJHBhcik9PSJULkFIIl0pID4gMCkgeyAgICAgICAgICAjICAgY2hlY2sgaWYgdXBwZXIgcGFycyBhcmUgYXZhaWxhYmxlDQogICAgICAgICAgVF9BSCA8LSAgYXMubnVtZXJpYyhBbVAkcGFyW3Jvd25hbWVzKEFtUCRwYXIpPT0iVC5BSCJdKSAgICAgIyAJdXBwZXIgQXJyaGVuaXVzIHRlbXBlcmF0dXJlDQogICAgICAgICAgVF9IICAgPC0gYXMubnVtZXJpYyhBbVAkcGFyW3Jvd25hbWVzKEFtUCRwYXIpPT0iVC5IIl0pICAgICAgIyAJdXBwZXIgYm91bmRhcnkgZm9yIEFycmhlbml1cyB0ZW1wZXJhdHVyZQ0KICAgICAgICAgICBwYXJfbmFtZXMgPSBjKHBhcl9uYW1lcywgIlRfQUgiLCAiVF9IIikNCiAgICAgICAgICAgIHBhcl92YWx1ZXM9IGMocGFyX3ZhbHVlcywgVF9BSCwgVF9IICkNCiAgICAgICAgICAgICBwYXJfZGVzY3JpcHRpb24gPSBjKHBhcl9kZXNjcmlwdGlvbiwgInVwcGVyIEFycmhlbml1cyB0ZW1wZXJhdHVyZSIsICJ1cHBlciBib3VuZGFyeSBmb3IgQXJyaGVuaXVzIHRlbXBlcmF0dXJlIikNCiAgICAgICAgfQ0KDQogICNzdHJlc3NlcyBmb3IgaW5zZWN0IGxpZmUgc3RhZ2VzIGZvbGxvd2luZyBEeWFyJ3MgTGF3IA0KICAgICAgaT0wDQogICAgICByZXBlYXQgew0KICAgICAgICBpID0gaSArMQ0KICAgICAgICBwID0gcGFzdGUgKCJzIixpLCBzZXA9Ii4iKQ0KICAgICAgICBpZihsZW5ndGgoQW1QJHBhcltyb3duYW1lcyhBbVAkcGFyKT09cF0pID09IDApIHticmVhaw0KICAgICAgICAgfSBlbHNlIHsNCiAgICAgICAgICAgYXNzaWduKHBhc3RlICgicyIsaSwgc2VwPSJfIikgLGFzLm51bWVyaWMoQW1QJHBhcltyb3duYW1lcyhBbVAkcGFyKT09cF0pKSAjIHN0cmVzcyBhdCBpbnN0YXINCiAgICAgICAgICAgIHBhcl9uYW1lcyA9IGMocGFyX25hbWVzLCBwYXN0ZSgicyIsaSwgc2VwPSJfIikpDQogICAgICAgICAgICAgIHBhcl92YWx1ZXM9IGMocGFyX3ZhbHVlcywgYXMubnVtZXJpYyhBbVAkcGFyW3Jvd25hbWVzKEFtUCRwYXIpPT1wXSkpDQogICAgICAgICAgICAgICAgcGFyX2Rlc2NyaXB0aW9uID0gYyhwYXJfZGVzY3JpcHRpb24sIHBhc3RlICgic3RyZXNzIGF0IGluc3RhciIsaSwgc2VwPSIgIikpDQogICAgICAgICAgICAgfQ0KICAgICAgfQ0KICAgICAgc3RhZ2VzID0gaSAjIG51bWJlciBvZiBsYXJ2YWwgc3RhZ2VzDQogICAgICAgcGFyX25hbWVzID0gYyhwYXJfbmFtZXMsInN0YWdlcyIpDQogICAgICAgIHBhcl92YWx1ZXM9IGMocGFyX3ZhbHVlcywgc3RhZ2VzKQ0KICAgICAgICAgcGFyX2Rlc2NyaXB0aW9uID0gYyhwYXJfZGVzY3JpcHRpb24sICJudW1iZXIgb2YgbGFydmFsIHN0YWdlcyIpDQogIA0KICAjQ3JlYXRlIGFuZCBzYXZlIHBhYW10ZXIgdGFibGUNCiAgICAgIHBhcl90YWJsZSA8LSBhcy5kYXRhLmZyYW1lKCBjYmluZCAocGFyX25hbWVzLHBhcl92YWx1ZXMsIHBhcl9kZXNjcmlwdGlvbikpDQogICAgICBuYW1lcyhwYXJfdGFibGUpIDwtIGMoInN5bWJvbCIsICJ2YWx1ZSIsICJkZXNjcmlwdGlvbiIpDQogICAgIA0KICAgICAgcGFyX3RhYmxlDQoNCmBgYA0KDQoNCjFkLiBSZWFkIGltcGxpZWQgcHJvcGVydGllcyBmcm9tIC5odG1sIGZpbGUgKEFtUCkgYW5kIGRlZmluZSBhZGRpdGlvbmFsIHBhcmFtZXRlcnMgDQoNCmBgYHtyfQ0KIyBSZWFkIHRoZSBQYXJhbWV0ZXJzIGFuZCBpbXBsaWVkIHByb3BlcnRpZXMgZnJvbSByZXBvcnQgaHRtbCBmaWxlDQogcmVwb3J0cGFnZSA8LSByZWFkX2h0bWwoInJlcG9ydF9CZW1pc2lhX3RhYmFjaS5odG1sIikNCiAgdGFibGVfbm9kZSA8LSBodG1sX25vZGUocmVwb3J0cGFnZSwgInRhYmxlIikgIyBFeHRyYWN0IHRoZSB0YWJsZSBub2RlIGFuZCBjb252ZXJ0IGl0IHRvIGEgZGF0YSBmcmFtZQ0KICAgIEFtUDIgPC0gaHRtbF90YWJsZSh0YWJsZV9ub2RlLCBmaWxsID0gVFJVRSwgaGVhZGVyID0gRkFMU0UpDQogICAgICBBbVAyIDwtIEFtUDIgIFszOm5yb3coQW1QMiApLF0NCiAgICAgICAgY29sbmFtZXMoQW1QMiApPC0gYygic3ltYm9sIiwgInVuaXRzIiwgInZhbHVlIiwgIkMiLCAiZnVuY19yZXNwIiwgImRlc2NyaXB0aW9uIikNCiAgDQogIEVfMCAgICAgIDwtIGFzLm51bWVyaWMoIEFtUDIkdmFsdWVbQW1QMiRzeW1ib2w9PSJFXzAiXSkgICAgICMgSiwgaW5pdGlhbCByZXNlcnZlDQogIExfYiAgICAgIDwtIGFzLm51bWVyaWMoIEFtUDIkdmFsdWVbQW1QMiRzeW1ib2w9PSJMX2IiXSkgICAgICMgY20sIHN0cnVjdHVyYWwgbGVuZ3RoIGF0IGJpcnRoIGF0IGY9MQ0KICBMX3AgICAgICA8LSBhcy5udW1lcmljKCBBbVAyJHZhbHVlW0FtUDIkc3ltYm9sPT0iTF9wIl0pICAgICAjIGNtLCBzdHJ1Y3R1cmFsIGxlbmd0aCBhdCBwdWJlcnR5IGF0IGY9MQ0KICBmICAgPC0gMSAgICAgICAgIyAtLCBzY2FsZWQgZnVuY3Rpb25hbCByZXNwb25zZQ0KICANCiANCiAgIyBiYWNrZ3JvdW5kIGhhemFyZCBkdXJpbmcgbGFydmFsIGRldmVsb3BtZW50DQogICAgIGhfYiAgICA8LSAgMC4wNDIzDQogIA0KICAjYWRkIHBhcmFtZXRlcnMgdG8gdmVjdG9ycw0KICBwYXJfbmFtZXM9TlVMTDsgcGFyX3ZhbHVlcz1OVUxMO3Bhcl9kZXNjcmlwdGlvbj1OVUxMDQogICAgICBwYXJfbmFtZXMgPSBjKHBhcl9uYW1lcywiRV8wIiwgIkxfYiIsICJMX3AiLCAiZiIsICJoX2IiKQ0KICAgICAgICBwYXJfdmFsdWVzPSBjKHBhcl92YWx1ZXMsIEVfMCwgTF9iLCBMX3AsIGYsIGhfYikNCiAgICAgICAgICAgcGFyX2Rlc2NyaXB0aW9uID0gYyhwYXJfZGVzY3JpcHRpb24sICJpbml0aWFsIHJlc2VydmUiLCAic3RydWN0dXJhbCBsZW5ndGggYXQgYmlydGgiLCAic3RydWN0dXJhbCBsZW5ndGggYXQgcHViZXJ0eSIsIA0KICAgICAgICAgICAgICAgICAgICAgICAgICAgICAgICJzY2FsZWQgZnVuY3Rpb25hbCByZXNwb25zZSIsICJiYWNrZ3JvdW5kIGhhemFyZCBkdXJpbmcgbGFydmFsIGRldmVsb3BtZW50IikNCiAgDQogIHBhcl90YWJsZTIgPC0gYXMuZGF0YS5mcmFtZSggY2JpbmQgKHBhcl9uYW1lcyxwYXJfdmFsdWVzLCBwYXJfZGVzY3JpcHRpb24pKQ0KICBuYW1lcyhwYXJfdGFibGUyKSA8LSBjKCJzeW1ib2wiLCAidmFsdWUiLCAiZGVzY3JpcHRpb24iKQ0KICBwYXJfdGFibGUyIA0KYGBgDQoNCg0KMWUuIFJlYWQgaW1wbGllZCBwcm9wZXJ0aWVzIGZyb20gLmh0bWwgZmlsZSAoQW1QKSBhbmQgZGVmaW5lIGFkZGl0aW9uYWwgcGFyYW1ldGVycyANCg0KYGBge3J9DQojIGFzc2lnbiBwYXJhbWV0ZXIgdmFsdWVzDQprZCA8LQkwLjA4NTAzCSAgI2RvbWluYW50IHJhdGUgY29uc3RhbnQNCnpiIDwtCTAuMDAzOTY5CSN0aHJlc2hvbGQgZm9yIGVmZmVjdHMNCmJiIDwtCTkuNDgyCSAgICAjZWZmZWN0IHN0cmVuZ3RoIGVuZXJneSBidWRnZXQNCmtrIDwtCTAuMjUJICAgICNzdHJlbmd0aCBsZXRoYWwgZWZmZWN0cw0KDQojYWRkIHBhcmFtZXRlcnMgdG8gdmVjdG9ycw0KICBwYXJfbmFtZXM9TlVMTDsgcGFyX3ZhbHVlcz1OVUxMO3Bhcl9kZXNjcmlwdGlvbj1OVUxMDQogICAgICBwYXJfbmFtZXMgPSBjKHBhcl9uYW1lcywia2QiLCAiemIiLCAiYmIiLCAia2siKQ0KICAgICAgICBwYXJfdmFsdWVzPSBjKHBhcl92YWx1ZXMsIGtkLCB6YiwgYmIsIGtrKQ0KICAgICAgICAgICBwYXJfZGVzY3JpcHRpb24gPSBjKHBhcl9kZXNjcmlwdGlvbiwgImRvbWluYW50IHJhdGUgY29uc3RhbnQiLCAidGhyZXNob2xkIGZvciBlZmZlY3RzIiwgImVmZmVjdCBzdHJlbmd0aCBlbmVyZ3kgYnVkZ2V0IiwgDQogICAgICAgICAgICAgICAgICAgICAgICAgICAgICAgInN0cmVuZ3RoIGxldGhhbCBlZmZlY3RzIikNCiAgDQogIHBhcl90YWJsZTMgPC0gYXMuZGF0YS5mcmFtZSggY2JpbmQgKHBhcl9uYW1lcyxwYXJfdmFsdWVzLCBwYXJfZGVzY3JpcHRpb24pKQ0KICBuYW1lcyhwYXJfdGFibGUzKSA8LSBjKCJzeW1ib2wiLCAidmFsdWUiLCAiZGVzY3JpcHRpb24iKQ0KICBwYXJfdGFibGUzIA0KDQpgYGANCg0KDQoxZi4gQ3JlYXRlIHBhcmFtZXRlciBpbnB1dCBmaWxlDQoNCmBgYHtyfQ0KIHBhcl90YWJsZXM8LSByYmluZChwYXJfdGFibGUsIHBhcl90YWJsZTIsIHBhcl90YWJsZTMpDQogd3JpdGUuY3N2KHBhcl90YWJsZXMsICJwYXJzLmNzdiIsIHJvdy5uYW1lcz1GQUxTRSkNCiBwcmludCgiLSBkb25lIC0iKQ0KYGBgDQoNCg0KMmEuIENyZWF0ZSB0ZW1wZXJhdHVyZSBzY2VuYXJpbw0KDQpgYGB7ciBmaWcuYWxpZ249LCBmaWcuaGVpZ2h0PTUsIGZpZy53aWR0aD01LCBmaWcuc2hvdz0iaG9sZCIsIG91dC53aWR0aD0iNTAlIiwgcGFnZWQucHJpbnQ9VFJVRX0NCiAgI2NyZWF0ZSBjb25zdGFudCB0ZW1wZXJhdHVyZSBzY2VuYXJpb3MtLS0tLS0tLS0tLS0tLS0tLS0tLS0tLS0tLS0tLS0tLS0tLS0tLS0tDQogICAgICB0aW1lczwtc2VxKDE6dG1heCkgI3RpbWUgdmVjdG9yDQogICAgICBzY2VuYXJpb3M8LTEgI251bWJlciBvZiBzY2VuYXJpb3MgdG8gYmUgY3JlYXRlZA0KICAgICAgDQogICAgICB0ZW1wZXJhdHVyZXMgPC0gbWF0cml4KGRhdGEgPSB0aW1lcywgbnJvdyA9IGxlbmd0aCh0aW1lcyksIG5jb2wgPSBzY2VuYXJpb3MrMSkgIyByZXN1bHRzIG1hdHJpeA0KICAgICAgZm9yICh4IGluIDE6c2NlbmFyaW9zKXsgDQogICAgICAgICBmb3IgKHQgaW4gMTpsZW5ndGgodGltZXMpKXsNCiAgICAgICAgICAgI3NldCBjb25zdGFudCB0ZW1wZXJhdHVyZSwgYWx0ZXJuYXRpdmVseSwgY3JlYXRlIHlvdXIgb3duIHRyYWplY3RvcnkNCiAgICAgICAgICAgdGVtcGVyYXR1cmVzIFt0LHgrMV0gPC0gMjYgDQogICAgICAgICB9DQogICAgICAgIA0KICAgICAgICAjcGxvdCB0ZW1wZXJhdHVyZQ0KICAgICAgICAgcGxvdCh0ZW1wZXJhdHVyZXMgWywxXSwgdGVtcGVyYXR1cmVzIFsseCsxXSwgdHlwZSA9ICJsIiwgIGx3ZD0zLCAgY29sID0gImxpZ2h0Ymx1ZTQiLCB5bGFiID0gIlRlbXBlcmF0dXJlIFtDXSIsIHhsYWIgPSAiVGltZSBbZF0iLCANCiAgICAgICAgICAgICAgY2V4LmF4aXM9MS4yLCBjZXgubGFiID0xLjIsIHlsaW0gPSBjKDE1LDQwKSwgeGxpbT1jKDAsMzUpLCAgYnR5PSJuIiAgKSAgIyAgDQogICAgICB9DQogICAgICB3cml0ZS5jc3YodGVtcGVyYXR1cmVzLCAidGVtcGVyYXR1cmVfc2NlbmFyaW9zLmNzdiIsIHJvdy5uYW1lcz0gRkFMU0UpIA0KDQpgYGANCg0KDQoyYi4gQ3JlYXRlIGV4cG9zdXJlIHNjZW5hcmlvDQoNCmBgYHtyIGZpZy5hbGlnbj0sIGZpZy5oZWlnaHQ9NSwgZmlnLndpZHRoPTUsfQ0KICAjc2NlbmFyaW8gc3BlY2lmaWNhdGlvbnMNCiAgICB0aW1pbmdzID0gYygxLCAxMCkgI3RpbWluZyAoZCkgb2YgYXBwbGljYXRpb24ocyksIGFkZCB0aW1pbmdzIGFzIG5lZWQNCiAgICAgIHRyZWF0bWVudHMgPSBjKDI0KSAjQXBwbGljYXRpb24gcmF0ZSBmb3IgZWFjaCB0aW1pbmcsIGcvaGENCiAgICANCiAgICBHQVAgPC0gbWF0cml4KE5hTiwgIG5yb3cgPSBsZW5ndGgodGltaW5ncyksIG5jb2wgPSAzKQ0KICAgIGNvbG5hbWVzKEdBUCkgPC0gYygiVHJlYXRtZW50LCAjIiwgIlRpbWluZywgZCIsICJSYXRlLCBnL2hhIikNCg0KICAjUGFyYW1ldGVycyBmb3IgdGVtcGVyYXR1cmUgY29ycmVjdGlvbiBhbmQgaGFsZiBsaWZlIChoZXJlOiBjb3R0b24pIA0KICAgIFRfcmVmICAgPC0yMCAgKyAyNzMuMTUgI3JlZmVyZW5jZSB0ZW1wZXJhdHVyZSBbQ10NCiAgICBUX0FfY290IDwtMy4zMzZlKzA0ICAgICNBcnJoZW5pdXMgdGVtcGVyYXR1cmUNCiAgICBUX0FIX2NvdCA8LSA0LjM5MmUrMDQgIyB1cHBlciBBcnJoZW5pdXMgdGVtcGVyYXR1cmUNCiAgICBUX0hfY290IDwtIDMwMS42ICAgICAjIHVwcGVyIGJvdW5kYXJ5IHRlbXBlcmF0dXJlDQogICAgaz0wLjA0MiAgI2RlY2F5IHBhcmFtZXRlciByZWxhdGVkIHRvIHByb2R1Y3QgaGFsZiBsaWZlDQogICAgDQogICMgcnVuIHRocm91Z2ggdGVtcGVyYXR1cmUgc2NlbmFyaW9zIChudW1iZXIgb2YgdGVtcGVyYXR1cmUgc2NlbmFyaW9zIG5lZWQgdG8gbWF0Y2ggdGhlIG51bWJlciBvZiBleHBvc3VyZSBzY2VuYXJpb3MpDQogICAgZXhwb3N1cmVzIDwtIG1hdHJpeChkYXRhID0gTkEsIG5yb3cgPSBsZW5ndGgodGltZXMpLCBuY29sID0gc2NlbmFyaW9zKzEpICMgcmVzdWx0cyBtYXRyaXgNCiAgICBleHBvc3VyZXMgWywxXTwtdGltZXMNCiAgICAgIA0KICAgIGZvciAoeCBpbiAxOnNjZW5hcmlvcyl7ICANCiAgICAgIA0KICAgICAgICNzcGVjaWZ5IHRyZWF0bWVudCBzZXJpZXMNCiAgICAgICAgICAgIFRyZWF0X3NlcmllcyAgICAgPC1yZXAoYygwKSwgbGVuZ3RoKHRpbWVzKSApDQogICAgICAgICAgICBUcmVhdF9zZXJpZXMgW3RpbWluZ3NdIDwtIHRyZWF0bWVudHMNCiAgICAgICAgICAgIENvbmMgPC0gVHJlYXRfc2VyaWVzWzFdDQogICAgICAgICAgICBleHBvc3VyZXMgWzEseCsxXSA8LSBDb25jICANCiAgICAgICAgDQogICAgICAgICNleHBvc3VyZSBmb3IgZWFjaCB0ZW1wZXJhdHVyZSBzY2VuYXJpbw0KICAgICAgICAgICAgZm9yICh0IGluIDI6bGVuZ3RoKHRpbWVzKSl7IA0KICAgICAgICAgICAgVDwtdGVtcGVyYXR1cmVzW3QseCsxXSsgMjczLjE1ICANCiAgICAgICAgICAgICAgc19BID0gZXhwKFRfQV9jb3QvIFRfcmVmIC0gVF9BX2NvdCAvIFQpICANCiAgICAgICAgICAgICAgICBzX0hfcmF0aW8gPSAoMSArIGV4cChUX0FIX2NvdC8gVF9IX2NvdCAtIFRfQUhfY290IC8gVF9yZWYpKSAvICgxICsgZXhwKFRfQUhfY290LyBUX0hfY290IC0gVF9BSF9jb3QgLyBUICAgICkpDQogICAgICAgICAgICAgICAgICBmX1QgPSBzX0EgKiAoVCA+PSBUX3JlZikgKiBzX0hfcmF0aW8NCiAgICAgICAgICANCiAgICAgICAgICAgIENvbmMgPC0gQ29uYyAqIGV4cCgtayAqIGZfVCkgKyBUcmVhdF9zZXJpZXNbdF0gICAgICAgICAgICAgICAgICAgICAgICAgICAgICAgICANCiAgICAgICAgICAgIGV4cG9zdXJlcyBbdCx4KzFdIDwtIHJvdW5kIChDb25jLDMpICAgDQogICAgICAgICAgICB9IyBsb29wIHRpbWUNCiAgICAgICANCiAgICAgICAgI3Bsb3QgZXhwb3N1cmVzDQogICAgICAgIHBsb3QoZXhwb3N1cmVzIFssMV0sIGV4cG9zdXJlcyBbLHgrMV0sIHR5cGUgPSAibCIsICBsd2Q9MywgIGNvbCA9ICJkYXJrb3JhbmdlMiIsIHlsYWIgPSAiQ29uY2VudHJhdGlvbiBbZy9oYV0iLCB4bGFiID0gIlRpbWUgW2RdIiwgDQogICAgICAgICAgICBtYWluID0gIHBhc3RlICgiU2NlbmFyaW8gIiwgeCksIGNleC5heGlzPTEsIGNleC5sYWIgPTEsICAgIGJ0eT0ibiIsIHhsaW09YygwLDQwKSwgeWxpbSA9IGMoMCwyNSkgKSAgIw0KICAgICB9DQogICAgICAgIA0KICAgIHdyaXRlLmNzdihleHBvc3VyZXMsICJleHBvc3VyZV9zY2VuYXJpb3MuY3N2Iiwgcm93Lm5hbWVzPSBGQUxTRSkgDQoNCmBgYA0KDQoNCjMuIEV4ZWN1dGUgdGhlIERFQmlibSBleGVjdXRhYmxlDQoNCmBgYHtyfQ0KcHJvY2Vzc3g6OnJ1bignREVCaWJtLmV4ZScpDQpwcmludCgiLSBkb25lIC0iKQ0KYGBgDQoNCg0KNCAmIDVhLiBDYWxjdWxhdGUgbWVhbnMgYW5kIHF1YW50aWxlczsgbWFrZSBmaWd1cmVzIGNvbXBhcmluZyB0b3RhbCBwb3B1bGF0aW9uIHNpemVzIGZvciB1bnRyZWF0ZWQgY29udHJvbCBhbmQgdHJlYXRtZW50Lg0KDQpgYGB7ciBlY2hvPVRSVUUsIGZpZy5hbGlnbj0sIGZpZy5oZWlnaHQ9NSwgZmlnLndpZHRoPTUsIGZpZy5zaG93PSJob2xkIiwgb3V0LndpZHRoPSI1MCUiLCBwYWdlZC5wcmludD1UUlVFfQ0KTUNtYXg8LXBhc3RlICgiTUNfIiwgTUNfbnVtYmVyLCBzZXA9IiIgKSAjbWF4aW11bSBudW1iZXIgb2YgTUMgc2ltdWxhdGlvbnMgZm9yIGNhbGN1bGF0aW9uIG9mIG1lYW5zIGFuZCBxdWFudGlsZXMgDQoNCiMtLWxvb3Agc2NlbmFyaW9zLS0tLS0tLS0tLS0tLS0tLS0tLS0tLS0tLS0tLS0tLS0tLS0tLS0tLS0tLS0tLS0tLS0tLS0tLS0tLS0tLS0tLS0tLS0tLS0tLS0tDQogICAgICBmb3IgKHggaW4gMTpzY2VuYXJpb3MpIHsNCiAgICAgIGZpbGVuYW1lIDwtIHBhc3RlICgicmVzdWx0cy9zY2VuYXJpb18iLCB4LCAiLmNzdiIsIHNlcD0iIiApDQogICAgICBvdXRwdXQ8LSBhcy5kYXRhLmZyYW1lKCByZWFkLmNzdihmaWxlbmFtZSwgaGVhZGVyID0gVFJVRSkpDQogICAgICANCiAgICAgICMtLWNhbGN1bGF0ZSBtZWFuIGFuZCBxdWFudGlsZXMtLS0tLS0tLS0tLS0tLS0tLS0tLS0tLS0tLS0tLS0tLS0tLS0tLS0tLS0tLS0tLS0tDQogICAgICAgICAgb3V0cHV0PC1hbGxfb2Yob3V0cHV0ICU+JQ0KICAgICAgICAgIHJvd3dpc2UoKSAlPiUNCiAgICAgICAgICBtdXRhdGUoIA0KICAgICAgICAgICAgbWVhbl92YWx1ZSA9IG1lYW4oY19hY3Jvc3MoTUNfMTpNQ21heCkpLA0KICAgICAgICAgICAgUV8xID0gcXVhbnRpbGUoY19hY3Jvc3MoTUNfMTpNQ21heCksIHByb2JzID0gYygwLjAyNSkpLA0KICAgICAgICAgICAgUV8yID0gcXVhbnRpbGUoY19hY3Jvc3MoTUNfMTpNQ21heCksIHByb2JzID0gYygwLjUpKSwNCiAgICAgICAgICAgIFFfMyA9IHF1YW50aWxlKGNfYWNyb3NzKE1DXzE6TUNtYXgpLCBwcm9icyA9IGMoMC45NzUpKSwNCiAgICAgICAgICAgKSkNCiAgICAgIG91dHB1dF9zdW0gPC1vdXRwdXRbLCBjb2xuYW1lcyhvdXRwdXQpICVpbiUgYygiZ3JvdXAiLCJzdWJwb3B1bGF0aW9uIiwiZGF5IiwgIm1lYW5fdmFsdWUiLCAiUV8xIiwgIlFfMiIsICJRXzMiKV0NCiAgICAgIA0KICAgICAgIy0tcXVhbnRpbGVzIHNlcGFyYXRlZCBieSBDb250cm9sIGFuZCB0cmVhdG1lbnQtLS0tLS0tLS0tLS0tLS0tLS0tLS0tLS0tLS0tLS0tLS0NCiAgICAgICAgY3RybF9zdW0gPC0gb3V0cHV0X3N1bVtvdXRwdXRfc3VtJGdyb3VwID09ICJjb250cm9sIiwgXSANCiAgICAgICAgdHJlYXRfc3VtIDwtIG91dHB1dF9zdW1bb3V0cHV0X3N1bSRncm91cCA9PSAidHJlYXRtZW50IiwgXSANCiAgICAgIA0KICAgICAgIy0tcXVhbnRpbGVzIG9mIHRvdGFsIGNvdW50cyBzZXBhcmF0ZWQgYnkgQ29udHJvbCBhbmQgdHJlYXRtZW50LS0tLS0tLS0tLS0tLS0tLS0NCiAgICAgICAgY3RybF90b3RhbHMgPC0gY3RybF9zdW1bY3RybF9zdW0kc3VicG9wdWxhdGlvbj09ICJ0b3RhbCIsIF0gIA0KICAgICAgICB0cmVhdF90b3RhbHMgPC0gdHJlYXRfc3VtW3RyZWF0X3N1bSRzdWJwb3B1bGF0aW9uPT0gInRvdGFsIiwgXSANCiAgICAgIA0KICAgICAgIy0tcGxvdCBwb3B1bGF0aW9uIGR5bmFtaWNzLS0tLS0tLS0tLS0tLS0tLS0tLS0tLS0tLS0tLS0tLS0tLS0tLS0tLS0tLS0tLS0tLS0tLS0NCiAgICAgICAgY29sX2N0cl9MPC0ibGlnaHRibHVlNCI7IGNvbF9jdHJfUDwtImxpZ2h0Ymx1ZTMiOyBjb2xfVF9MIDwtImRhcmtvcmFuZ2UyIjsgY29sX1RfUCA8LSAib3JhbmdlIg0KICAgICAgICBwbG90KGN0cmxfdG90YWxzJGRheSwgY3RybF90b3RhbHMkbWVhbl92YWx1ZSwgdHlwZSA9ICJsIiwgIGx3ZD0yLCB5bGFiID0gIlBvcHVsYXRpb24gc2l6ZSBbI10iLCANCiAgICAgICAgICAgICB4bGFiID0gIlRpbWUgW2RdIiwgY2V4LmF4aXM9MSwgY2V4LmxhYiA9MSwgICAgYnR5PSJuIiwgeWxpbT1jKDAsbWF4KGN0cmxfdG90YWxzJFFfMykpICkgICMNCiAgICAgICAgICAgIHBvbHlnb24oYyhjdHJsX3RvdGFscyRkYXksIHJldihjdHJsX3RvdGFscyRkYXkpKSwgYyhjdHJsX3RvdGFscyRRXzMgLHJldihjdHJsX3RvdGFscyRRXzEpKSwgYm9yZGVyID0gTkEgLCBjb2wgPSBjb2xfY3RyX1ApDQogICAgICAgICAgICAgIHBvbHlnb24oYyh0cmVhdF90b3RhbHMkZGF5LCByZXYodHJlYXRfdG90YWxzJGRheSkpLCBjKHRyZWF0X3RvdGFscyRRXzMgLHJldih0cmVhdF90b3RhbHMkUV8xKSksIGJvcmRlciA9IE5BICwgY29sID0gY29sX1RfUCkNCiAgICAgICAgICAgICAgICBsaW5lcyhjdHJsX3RvdGFscyRkYXksIGN0cmxfdG90YWxzJG1lYW5fdmFsdWUsIHR5cGUgPSAibCIsbHdkPTMsIGNvbCA9IGNvbF9jdHJfTCkNCiAgICAgICAgICAgICAgICAgICAgbGluZXModHJlYXRfdG90YWxzJGRheSwgdHJlYXRfdG90YWxzJG1lYW5fdmFsdWUsIHR5cGUgPSAibCIsbHdkPTMsIGNvbCA9IGNvbF9UX0wpIA0KfSAjZW5kIGxvb3Agc2NlbmFyaW9zICAgICAgICAgICAgICANCg0KYGBgDQoNCg0KNWIuSGlzdG9ncmFtcyBmb3IgcG9wdWxhdGlvbiBkZW1vZ3JhcGh5IGJhc2VkIG9uIG1lYW5zIG9mIE1vbnRlLUNhcmxvIHNpbXVsYXRpb25zDQoNCmBgYHtyICwgZmlnLmhlaWdodD01LCBmaWcud2lkdGg9MTB9DQpzdGFnZXM9NA0KIy0tcGxvdCBzZXR0aW5ncw0KI2Nob29zZSBwbG90IGNvbG9ycyBhY2NvcmRpbmcgdG8gbnVtYmVyIG9mIHN0YWdlcw0KcGxvdF9jb2wgPC1jKCJvcmFuZ2UiLCJsaWdodGJsdWU0IiwgImxpZ2h0Ymx1ZTMiLCJsaWdodGJsdWUyIiwibGlnaHRibHVlMSIsImJsYWNrIikNCg0KI3NjYWxlIHktYXhpcyBvZiB0cmVhdG1lbnQgcGxvdCBzYW1lIGFzIGNvbnRyb2w/DQpjdHJsX3NjYWxlIDwtIFRSVUUgI1RSVUUvRkFMU0UNCg0KIy0tbG9vcCBzY2VuYXJpb3MtLS0tLS0tLS0tLS0tLS0tLS0tLS0tLS0tLS0tLS0tLS0tLS0tLS0tLS0tLS0tLS0tLS0tLS0tLS0tLS0tLS0tLS0tLS0tLS0tLS0NCiAgZm9yICh4IGluIDE6c2NlbmFyaW9zKSB7DQogICAgICBmaWxlbmFtZSA8LSBwYXN0ZSAoInJlc3VsdHMvc2NlbmFyaW9fIiwgeCwgIi5jc3YiLCBzZXA9IiIgKQ0KICAgICAgb3V0cHV0PC0gYXMuZGF0YS5mcmFtZSggcmVhZC5jc3YoZmlsZW5hbWUsIGhlYWRlciA9IFRSVUUpKQ0KDQogICAgICAjLS1jYWxjdWxhdGUgbWVhbnMNCiAgICAgICAgICBvdXRwdXQ8LWFsbF9vZihvdXRwdXQgJT4lDQogICAgICAgICAgcm93d2lzZSgpICU+JQ0KICAgICAgICAgIG11dGF0ZSgNCiAgICAgICAgICAgIG1lYW5fdmFsdWUgPSBtZWFuKGNfYWNyb3NzKE1DXzE6TUNtYXgpKSwgKSkNCiAgICAgICAgICBvdXRwdXRfc3VtIDwtb3V0cHV0WywgY29sbmFtZXMob3V0cHV0KSAlaW4lIGMoImdyb3VwIiwic3VicG9wdWxhdGlvbiIsImRheSIsICJtZWFuX3ZhbHVlIildDQoNCg0KICAgICAgIy0tc3VtbWFyaXplIGRhdGEgYW5kIG1ha2UgaGlzdG9ncmFtcw0KICAgICAgICAgIHBhcihtZnJvdyA9IGMoMSwgMikpDQogICAgICAgICAgZm9yIChpIGluIDE6Mikgew0KICAgICAgICAgICAgIy0tcXVhbnRpbGVzIGZvciBDb250cm9sIGFuZCB0cmVhdG1lbnQNCiAgICAgICAgICAgICAgICBpZiAoaT09MSkgew0KICAgICAgICAgICAgICAgICAgICAjZmlsdGVyIGZvciBjb250cm9sIGRhdGENCiAgICAgICAgICAgICAgICAgICAgaW5zdGFyc19zdW0gPC0gb3V0cHV0X3N1bVtvdXRwdXRfc3VtJGdyb3VwID09ICJjb250cm9sIiwgXQ0KICAgICAgICAgICAgICAgICAgICAjbWFpbiB0aXRsZSBmb3IgcGxvdA0KICAgICAgICAgICAgICAgICAgICB0aXRsZTwtICJVbnRyZWF0ZWQgY29udHJvbCINCiAgICAgICAgICAgICAgICAgICAgI3NlYXJjaCBmb3IgbWF4aW11bSB2YWx1ZSBmb3IgeWxpbQ0KICAgICAgICAgICAgICAgICAgICB5X21heCA8LSBzaWduaWYoIG1heChvdXRwdXRfc3VtW291dHB1dF9zdW0kc3VicG9wdWxhdGlvbiA9PSAidG90YWwiLCA0XSksIGRpZ2l0cyA9IDEpDQoNCiAgICAgICAgICAgICAgICB9IGVsc2Ugew0KICAgICAgICAgICAgICAgICAgICAjZmlsdGVyIGZvciB0cmVhdG1lbnQgZGF0YQ0KICAgICAgICAgICAgICAgICAgICBpbnN0YXJzX3N1bSA8LSBvdXRwdXRfc3VtW291dHB1dF9zdW0kZ3JvdXAgPT0gInRyZWF0bWVudCIsIF0NCiAgICAgICAgICAgICAgICAgICAgI21haW4gdGl0bGUgZm9yIHBsb3QNCiAgICAgICAgICAgICAgICAgICAgdGl0bGU8LSAiVHJlYXRtZW50Ig0KDQogICAgICAgICAgICAgICAgICAgICNzZWFyY2ggZm9yIG1heGltdW0gdmFsdWUgZm9yIHlsaW0gaW4gdHJlYXRtZW50DQogICAgICAgICAgICAgICAgICAgIGlmIChjdHJsX3NjYWxlID09IEZBTFNFKSB5X21heCA8LSBzaWduaWYoIG1heChvdXRwdXRfc3VtW291dHB1dF9zdW0kZ3JvdXAgPT0gInRyZWF0bWVudCIsIDRdKSwgZGlnaXRzID0gMSkNCiAgICAgICAgICAgICAgICAgICAgfQ0KDQogICAgICAgICAgICAjLS1tYXRyaXggZm9yIGRpZmZlcmVudCBzdGFnZXMNCiAgICAgICAgICAgICAgICBkYXkgPC0gZGF0YS5mcmFtZSggZGF5ID0gYygxOnRtYXgpKQ0KICAgICAgICAgICAgICAgIGRmX2luc3RhcnM8LWFzLmRhdGEuZnJhbWUgKGMoMTp0bWF4KSkNCiAgICAgICAgICAgICAgICAjIGVnZ3MNCiAgICAgICAgICAgICAgICAgIEwgPC1hcy5kYXRhLmZyYW1lIChpbnN0YXJzX3N1bVtpbnN0YXJzX3N1bSRzdWJwb3B1bGF0aW9uID09ICJlZ2dzIiwgNF0gKQ0KICAgICAgICAgICAgICAgICAgZGZfaW5zdGFyczwtIGNiaW5kKGRmX2luc3RhcnMsTCkNCiAgICAgICAgICAgICAgICAgICAgY29sbmFtZXMoZGZfaW5zdGFycyk8LWMoImRheSIsICJlZ2dzIikNCiAgICAgICAgICAgICAgICAjIGxhcnZhbCBzdGFnZXMNCiAgICAgICAgICAgICAgICBmb3IgKGkgaW4gMTpzdGFnZXMpIHsNCiAgICAgICAgICAgICAgICAgICAgczwtcGFzdGUgKCJpbnN0YXIiLGksIHNlcCA9ICIiKQ0KICAgICAgICAgICAgICAgICAgICBMIDwtYXMuZGF0YS5mcmFtZSAoaW5zdGFyc19zdW1baW5zdGFyc19zdW0kc3VicG9wdWxhdGlvbiA9PSBzLCA0XSApDQogICAgICAgICAgICAgICAgICAgIGNvbG5hbWVzKEwgKTwtcw0KICAgICAgICAgICAgICAgICAgICBkZl9pbnN0YXJzPC0gY2JpbmQoZGZfaW5zdGFycyxMKQ0KICAgICAgICAgICAgICAgIH0NCiAgICAgICAgICAgICAgICAjIGFkdWx0cw0KICAgICAgICAgICAgICAgIEwgPC1hcy5kYXRhLmZyYW1lIChpbnN0YXJzX3N1bVtpbnN0YXJzX3N1bSRzdWJwb3B1bGF0aW9uID09ICJhZHVsdHMiLCA0XSApDQogICAgICAgICAgICAgICAgY29sbmFtZXMoTCApPC0iYWR1bHRzIg0KICAgICAgICAgICAgICAgIGRmX2luc3RhcnM8LSBjYmluZChkZl9pbnN0YXJzLEwpDQoNCg0KICAgICAgICAgIy0taGlzdG9ncmFtcw0KICAgICAgICAgICAgICBkdGE8LXQoYXMubWF0cml4KGRmX2luc3RhcnNbLDI6KHN0YWdlcyszKV0pKQ0KICAgICAgICAgICAgICAgIGJhcnBsb3QoZHRhLA0KICAgICAgICAgICAgICAgIG1haW4gPSB0aXRsZSwNCiAgICAgICAgICAgICAgICB4bGFiID0gIlRpbWUgW2RdIiwNCiAgICAgICAgICAgICAgICB5bGFiID0gIkNvdW50IFsjXSIsDQogICAgICAgICAgICAgICAgY2V4LmF4aXM9MSwgY2V4LmxhYiA9MSwNCiAgICAgICAgICAgICAgICB5bGltID0gYygwLCB5X21heCksDQogICAgICAgICAgICAgICAgI3lsaW0gPSBjKDAsIDEwMDApLA0KICAgICAgICAgICAgICAgIGF4ZXMgPSBUUlVFLA0KICAgICAgICAgICAgICAgIGxlZ2VuZC50ZXh0ID0gcm93bmFtZXMoZHRhKSwNCiAgICAgICAgICAgICAgICBhcmdzLmxlZ2VuZCA9IGxpc3QoeCA9ICJ0b3BsZWZ0IiwgaW5zZXQgPSBjKDAuMDUsIDApKSwNCiAgICAgICAgICAgICAgICBjb2w9cGxvdF9jb2wsDQogICAgICAgICAgICAgICAgbmFtZXM9ZGZfaW5zdGFyc1ssMV0NCiAgICAgICAgICAgICAgICApDQogICAgICB9ICNsb29wIGNvbnRyb2wvdHJlYXRtZW50DQoNCn0jbG9vcCBzY2VuYXJpb3MNCg0KYGBgDQoNCg0KNWMuIFBsb3QgZWZmaWNhY3kNCg0KYGBge3IgZmlnLmhlaWdodD01LCBmaWcud2lkdGg9NX0NCiAjLS1lZmZpY2FjeSBxdWFudGlsZXMgZm9yIHRvdGFsIGNvdW50cw0KICAgIGVmZmljYWN5X3RvdGFsczwtdHJlYXRfdG90YWxzDQogICAgICBlZmZpY2FjeV90b3RhbHMkUV8xPC0xLWVmZmljYWN5X3RvdGFscyRRXzEvY3RybF90b3RhbHMkUV8yDQogICAgICAgIGVmZmljYWN5X3RvdGFscyRRXzI8LTEtZWZmaWNhY3lfdG90YWxzJFFfMi9jdHJsX3RvdGFscyRRXzINCiAgICAgICAgICBlZmZpY2FjeV90b3RhbHMkUV8zPC0xLWVmZmljYWN5X3RvdGFscyRRXzMvY3RybF90b3RhbHMkUV8yDQogICAgICAgICAgDQpwbG90KGVmZmljYWN5X3RvdGFscyRkYXksIGVmZmljYWN5X3RvdGFscyRRXzIsIHR5cGUgPSAibCIsICBsd2Q9MiwgDQogICAgIHlsYWIgPSAiRWZmaWNhY3kgWy1dIiwgbWFpbiA9ICAiVG90YWwgZWZmaWNhY3kiLCBjZXgubWFpbiA9MS4yICwNCiAgICAgICAgIHhsYWIgPSAiVGltZSBbZF0iICwgY2V4LmF4aXM9MS4yLCBjZXgubGFiID0xLjIsICAgIGJ0eT0ibiIsIHlsaW09YygwICwxLjA1KSApICAjDQogICAgICAgICAgICBwb2x5Z29uKGMoZWZmaWNhY3lfdG90YWxzJGRheSwgcmV2KGVmZmljYWN5X3RvdGFscyRkYXkpKSwgDQogICAgICAgICAgICAgICAgICAgIGMoZWZmaWNhY3lfdG90YWxzJFFfMyAscmV2KGVmZmljYWN5X3RvdGFscyRRXzEpKSwgDQogICAgICAgICAgICAgICAgICAgICAgYm9yZGVyID0gTkEgLCBjb2wgPSAibGlnaHRibHVlMyIpIA0KICAgICAgICAgICAgICAgbGluZXMoZWZmaWNhY3lfdG90YWxzJGRheSwgZWZmaWNhY3lfdG90YWxzJFFfMiwgdHlwZSA9ICJsIixsd2Q9MywgY29sID0gImxpZ2h0Ymx1ZTQiKQ0KYGBgDQo=
